# Climate Shapes Elevational Gradients in Concentration, Composition, and Functional Diversity of Willow Metabolomes

**DOI:** 10.1111/pce.70775

**Published:** 2026-07-31

**Authors:** Sofian A. Renoult, Priscila Mezzomo, Jing V. Leong, Pavel Sebek, Shristee Panthee, Jaroslav Kukla, Petr Nguyen, Martin Moos, Dale L. Forrister, Natascha D. Wagner, Elvira Hörandl, Petr Koutecký, Brian E. Sedio, Jan Frouz, Nicole M. van Dam, Martin Volf

**Affiliations:** ^1^ Biology Centre CAS Institute of Entomology Ceske Budejovice Czechia; ^2^ Faculty of Science University of South Bohemia Ceske Budejovice Czechia; ^3^ Leibniz Institute for Horticultural Sciences (IGZ) e.V. Großbeeren Branderburg Germany; ^4^ Institute of Biodiversity, Ecology and Evolution (IBEE) Friedrich Schiller University Jena Thuringia Germany; ^5^ Institute for Environmental Studies Charles University Praha Czechia; ^6^ Faculty of Agriculture and Technology University of South Bohemia Ceske Budejovice Czechia; ^7^ Department of Integrative Biology University of Texas at Austin Austin Texas USA; ^8^ Department of Systematics, Biodiversity and Evolution of Plants (with Herbarium) University of Göttingen Göttingen Lower Saxony Germany; ^9^ Smithsonian Tropical Research Institute Balboa Ancon Republic of Panama; ^10^ Biology Centre CAS Institute of Soil Biology and Biogeochemistry Ceske Budejovice Czechia

**Keywords:** Altitudinal gradients, chemical defences, flavonoids, salicinoids, *Salix*, specialized metabolites

## Abstract

Plants produce broad spectra of metabolites comprising primary metabolites involved in core physiological processes and specialized metabolites mediating responses to biotic and abiotic stress. Understanding how these various components of the metabolome respond to different environmental pressures can help us to understand drivers of phytochemical diversity and its role in plant ecology and evolution. We investigated how climatic stress, soil resources, and herbivory influence various measures of phytochemical diversity in four willow species along an elevational gradient (950–2250 m a.s.l.), focusing on flavonoids, salicinoids, and primary metabolites. Harsher climates increased metabolite concentrations, particularly salicinoids, but reduced flavonoid richness and overall structural α‐diversity. Similar responses in primary metabolites suggest that these shifts extend beyond defence chemistry. Chemical properties showed the strongest shifts in flavonoids and salicinoids and weaker changes in primary metabolites. β‐diversity generally increased with elevation and was most strongly associated with climate, while soil nutrients contributed to shifts in β‐diversity of salicinoids and primary metabolites. Willow species showed weak genetic differentiation across elevation, suggesting limited population divergence. Together, these results indicate that elevational shifts in willow chemistry involve broad but uneven reorganization across the metabolome, with different metabolite groups and measures of diversity plastically responding to different drivers.

## Summary statement

Climatic stress reshaped willow metabolomes along elevation, but these responses extended beyond specialised defence chemistry to primary metabolites. Soil conditions further modified β‐diversity, and weak genetic differentiation suggests these patterns do not reflect strong population divergence.

## 1 Introduction

Plants produce a wide diversity of metabolites that help them persist across contrasting environments (Bennett and Wallsgrove 1994; Kessler and Kalske 2018). These include primary metabolites that are mainly involved in basic physiological processes and specialized (secondary) metabolites that often serve defensive or protective functions (Taiz and Zeiger 2014; Erb and Kliebenstein 2020). Variation in phytochemical diversity and chemical strategies plays a key role in plant responses to both biotic and abiotic stress (Coley et al. 1985; Endara et al. 2023; Volf et al. 2023). This is particularly relevant along elevational gradients, where plants experience steep clines in temperature, resource availability, and biotic interactions (Defossez et al. 2018; Moreira et al. 2018). Recent advances in untargeted metabolomics now allow us to quantify multiple measures of phytochemical diversity, including metabolite concentration, α‐diversity within plants (e.g., metabolite richness), and β‐diversity among plant species or individuals, while also accounting for structural relatedness among metabolites (Sedio 2017; Volf et al. 2023). Such approaches enable us to not only assess whether plant chemistry changes along environmental gradients, but also which measures of phytochemical diversity respond most strongly to different sources of stress (Walker et al. 2022; Volf et al. 2024).

The Resource Availability Hypothesis predicts that plants growing in resource‐limited environments allocate more energy to defence in order to protect tissues that are costly to replace (Coley et al. 1985; Sun et al. 2026). This can explain why plants at higher elevations, where low temperatures and short growing seasons restrict nutrient cycling and compensatory regrowth, tend to invest more in physical and chemical defences (Sundqvist et al. 2013; Moreira et al. 2018). Consistent with this hypothesis, many studies have reported increased concentrations of defensive metabolites at higher elevations, despite a general elevational decrease in herbivory (Dostálek et al. 2016; Volf et al. 2020; Bakhtiari et al. 2021; Volf et al. 2022). However, these patterns are mechanistically difficult to interpret because increasing elevation integrates several environmental changes. In addition to lower temperatures, shorter growing seasons, and changes in moisture regime, high‐elevation plants often experience shifts in soil nutrient availability, all of which may affect plant chemistry (Defossez et al. 2018; Moreira et al. 2018; Volf et al. 2023; Kumari and Kumar 2024). As a result, it often remains unclear whether elevational shifts in phytochemical diversity primarily reflect climatic stress, soil‐mediated resource limitation, decrease in herbivory or all these factors.

This mechanistic uncertainty is reflected in broader literature, where elevational trends in plant chemistry are often not uniform across study systems, metabolite groups, or measures of phytochemical diversity (Moreira et al. 2018; Volf et al. 2020). In some cases, plants at higher elevations show increased concentrations or richness of defensive metabolites (Moreira et al. 2018; Volf et al. 2020; Nomoto et al. 2025b), whereas in others, these traits show hump‐shaped responses or decline under particularly extreme conditions (Pellissier et al. 2014; Sam et al. 2019; Volf et al. 2022). Such contrasting patterns suggest that different aspects of plant chemistry may respond to different environmental drivers and constraints (Kumari and Kumar 2024). They also indicate that broad elevational trends alone are often insufficient to explain why plant metabolomes shift across habitats.

A better mechanistic understanding is especially needed because different measures of phytochemical diversity may reflect different ecological functions. For example, a richer defensive chemistry may provide defence against a broader range of herbivores (Whitehead et al. 2021). In contrast, patterns in β‐diversity may reflect the extent to which co‐occurring plants converge on similar chemical strategies under shared environmental filtering. Conversely, the plants can diverge in their chemistry due to differences in resource use or partition their chemical niches in response to biotic interactions with insect herbivores (Becerra 2007; Kergunteuil et al. 2018; Henderson et al. 2025). However, changes in metabolite concentration or diversity do not necessarily reveal whether plants shift towards compounds with different transport properties, bioactivity, light‐absorbing capacity, or other functionally relevant features of metabolome composition. Recent findings show that a relatively small number of chemical properties related to metabolite structure can describe a substantial part of functional variation in plant metabolomes (Walker et al. 2023). Properties such as molecular weight, hydrophobicity, topological polar surface area, or the size of the largest conjugated π‐system may be linked to metabolite membrane transport, light absorption, and bioactivity, and may therefore provide a more functionally grounded perspective on elevational shifts in plant chemistry (Ertl et al. 2000; Lovering et al. 2009; Willighagen et al. 2017).

Willows (*Salix* L.) are woody plants that represent an ideal system for studying clines in phytochemical diversity along elevational gradients. Many species have broad elevational ranges and form diverse assemblages (Wagner et al. 2021). Their chemical defences are based primarily on diverse phenolics, which makes it possible to compare elevational responses in metabolite groups with partly contrasting ecological functions (Volf et al. 2022). Flavonoids in particular can contribute to protection against abiotic stress as well as herbivores, whereas salicinoids play key roles in plant–herbivore interactions by deterring generalists but also serving as feeding or oviposition cues for specialists (Ren et al. 2026; Tegelberg and Julkunen‐Tiitto 2001; Hjältén et al. 2007; Volf et al. 2015). Previous studies in willows have identified elevational shifts in metabolite concentration and diversity, but the mechanisms underlying these patterns remain poorly understood (Volf et al. 2022, 2023). In particular, it remains unclear to what extent these patterns reflect climatic stress, soil‐mediated resource limitation, broader changes in plant metabolic strategies, or differences in genetic structure across elevation.

In this study, we quantified the chemical, structural, and functional diversity of metabolites in four abundant *Salix* species growing along an elevational gradient in the Austrian Alps. We asked how multiple measures of phytochemical diversity vary with elevation and whether these shifts are more closely associated with climatic conditions, soil conditions, or herbivory. We further examined whether elevational metabolomic shifts extend to both primary and specialized metabolites, as well as to functionally relevant chemical properties. Previous studies have primarily interpreted elevational shifts in plant chemistry through changes in defence and protection against abiotic stress (e.g., Pellissier et al. 2016; Moreira et al. 2018; Volf et al. 2022). Analysing primary metabolites allows us to determine whether these patterns reflect shifts in defence alone or broader reorganization of the metabolome in response to stress. In addition, we explored genetic differentiation among populations across elevation to assess whether the observed patterns are more consistent with local adaptation or phenotypic plasticity. First, we hypothesized that metabolite concentration would increase with elevation, particularly under harsher climatic conditions and poorer soils, reflecting stronger investment in defence under stressful conditions. In contrast, we expected metabolite richness and structural α‐diversity to decline with elevation, especially in flavonoids, as stressful environments may favour a narrower set of compounds with high functional relevance. Second, we hypothesized that chemical properties of metabolites would respond to different stressors more specifically than metabolite concentration, richness, or structural α‐diversity. Third, we hypothesized that different measures of chemical β‐diversity would respond to elevation in a metabolite‐specific manner, with defence‐related chemistry being particularly sensitive to climatic stress and nutrient limitation.

## 2 Materials and Methods

### 2.1 Sampling Sites and Plant Identification

We conducted our study in July–August 2023 in Mölltal and Ködnitz valleys SE and S of Großglockner, Austria. In the two valleys, we selected 21 sites representing an elevational gradient spanning from 950 m to 2250 m a.s.l. (Table S1). We focused our sampling on four willow species that were particularly common along the gradient and together covered its full elevational range: *Salix appendiculata* Vill., *S. caprea* L., *S. mielichhoferi* Saut. and *S. myrsinifolia* Salisb. *S. appendiculata* and *S. caprea* are closely related diploid species and often hybridize. *Salix mielichhoferi* and *S. myrsinifolia* are hexaploid sister species that frequently form morphologically transitional forms (Hörandl et al. 2012; Wagner et al. 2023). Within each species pair, one species was more typical of lower elevations (*S. caprea, S. myrsinifolia*), whereas the other extended further towards higher elevations (*S. appendiculata, S. mielichhoferi*). This sampling design allowed us to represent the gradient with both diploid and hexaploid willows and to reduce the risk that elevational patterns would be confounded with ploidy alone. We studied *S. mielichhoferi* and *S. myrsinifolia* in both Mölltal and Ködnitz valleys, while we limited our study of *S. appendiculata* and *S. caprea* to the Mölltal valley for logistic reasons.

At each site, we mapped the occurrence of the focal plant species that we identified in the field based on the leaf, twig, and flower morphology, following Hörandl et al. (2012). If present and accessible, we then sampled up to five individuals per species, their potential hybrids, or morphologically transitional forms at each site, resulting in a total of 157 plants sampled. We recorded the GPS location and elevation for each plant individual (Table S1). The plants were at least 5 m apart. We collected vouchers from all the plants that were later examined by three of the co‐authors (EH, NDW and PK) to improve our field identifications. To further improve our identifications, we used RepeatExplorer2 (Novák et al. 2020) and analysed species‐specific repetitive sequences using low‐coverage genomic data. We also used these data to infer whether plants from different elevations showed clear genetic structure and formed separate groups when considering their repetitive sequences (see Appendix S1 and Figure 1 for details).

**Figure 1:**
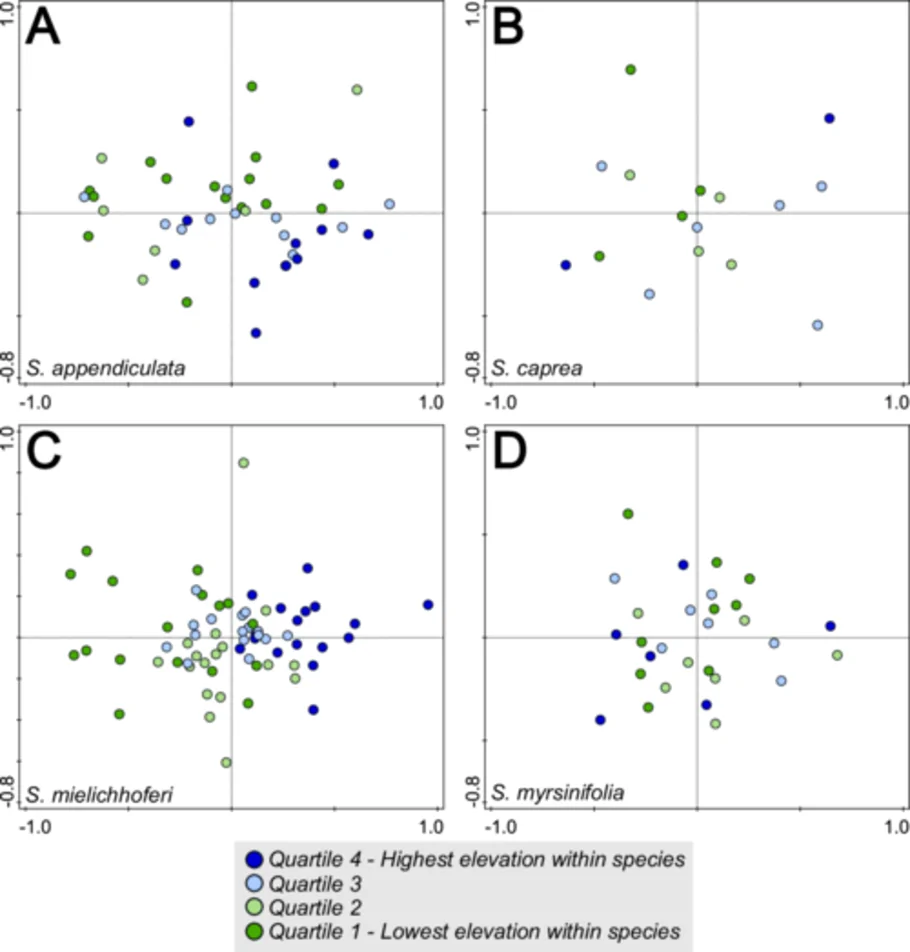
Variation in standardized read numbers for detected repetitive sequences clusters among individuals of *Salix appendiculata* (A), *Salix caprea* (B), *Salix mielichhoferi* (C) and *Salix myrsinifolia* (D), separated in quartiles based on elevation, visualized with principal component analysis (PCAs) using the valley as covariate.

We collected 20 freshly matured undamaged leaves from each plant and stored them in bags with silica gel in the field. On the same day, we transferred the leaves in liquid nitrogen where they were stored for the rest of our field sampling. We used these leaves for molecular analyses detailed in Appendix S1 and metabolomics analyses detailed below.

We haphazardly collected additional 50 freshly matured, fully expanded leaves of similar age from each individual to quantify herbivory damage. We photographed the leaves on a white background and quantified chewing damage in ImageJ 1.50i (Abràmoff et al. 2004). Other types of herbivory damage were scarce. For each plant, herbivory was calculated across the full set of 50 leaves as the percentage of leaf area removed by insect herbivores, with original leaf outlines reconstructed manually where necessary.

### 2.2 Metabolomics Analyses

We performed untargeted metabolomics to analyse the chemical profiles of the studied plants. Small organic molecules were extracted from ca. 10 mg (± 0.01 mg) of freeze‐dried and homogenized material using 1.8 mL methanol/water (90:10, v/v) solvent. The samples were extracted overnight at 4°C and 300 rpm, centrifuged at 14,000 rpm for 30 min. The supernatant was removed and filtered through a 0.2 μm PVDF mini‐spin filter (HPST, Prague, Czech Republic) at 7000 rpm, 4°C, and 5 min for analysis using UHPLC‐HRMS. Metabolic extracts were separated using a Thermo Fisher Scientific (Thermo Fisher Scientific, San Jose, CA, USA) Ultimate 3000 ultra‐high performance liquid chromatography (UHPLC) system with a Kinetex C18 column (100 mm × 3 mm i.d., 1.7 µm, Phenomenex, Torrance, CA, USA) the flow rate of 400 μL/min, the injection volume of 5 μL, column temperature of 25°C and mobile phase gradient: 0 min, 95% A; 2 min, 95% A; 27 min, 0% A; 35 min, 0% A; 37 min, 95% A; 40.0 min, 95% A, where A was (0.1% v/v formic acid in water) and B (0.1% v/v formic acid in acetonitrile). Separation of metabolites by UHPLC was followed by heated electrospray ionization (HESI) in positive mode using full MS scan and data‐dependent acquisition of MS^2^ (dd‐MS^2^) on a Thermo Fisher Scientific Q Exactive hybrid quadrupole‐orbitrap mass spectrometer. We collected an MS1 full scan (100–1500 *m/z*) at a resolution of 70,000. Automatic gain control target values were 3e6 for full MS scan and 1e5 for dd‐MS^2^. Maximum ion injection times were 100 ms for full MS scan, and 50 ms for dd‐MS^2^. For dd‐MS2, we set the isolation window to 4 *m/z* and normalized collision energy at 30. Source ionization parameters were as follows: 3.3 kV spray voltage, 350°C capillary temperature, sheath gas at 50 au, aux gas at 20 au, spare gas at 0 au, probe temperature 300°C and S‐Lens level at 60 au (Renoult et al. 2025).

The adjusted data were processed for peak detection, peak alignment, and peak filtering using MZmine 3 (Schmid et al. 2023). We used the same parameters as Sedio et al. (2021) except for setting MS1 noise threshold to 50,000 ion count and MS2 noise threshold to 1000 ion count. Our target compounds are eluted in the first 20 min of LC‐MS analysis, hence we filtered MS‐scans from this range only. Compounds that occurred in blanks, such as contaminants and industrial surfactants, were removed from further analyses. Using the MZmine 3 output, we inferred molecular formulae using SIRIUS (Dührkop et al. 2019), predicted structures using CSI:FingerID (Dührkop et al. 2015), and classified the metabolites using CANOPUS (Dührkop et al. 2021). Using this information, we created four datasets including (i) all detected metabolites, (ii) flavonoids, (iii) salicinoids plus their derivatives, and (iv) primary metabolites (including carbohydrates and fatty acids). We manually checked the classification of salicinoids based on the predicted structures, represented as text strings using the Simplified Molecular Input Line Entry System (SMILES).

For each of the four datasets, we quantified several measures of phytochemical diversity and metabolite properties. We first quantified metabolite concentrations as peak areas/mg of dry sample weight. We then quantified two measures of phytochemical α‐diversity: richness and α‐structural diversity. We quantified metabolite richness as the number of metabolites per sample. We calculated structural α‐diversity of metabolites in a sample as Functional Hill Diversity using the “chemodiv” R package with *q* = 1 (Petrén et al. 2023). Briefly, we used predicted SMILES and the function “compDis” to calculate dissimilarity among individual metabolites, which we then used to quantify the structural diversity of metabolites in the samples using the function “calcDiv” (Table S2).

Second, we quantified five chemical properties of the metabolites which are representative of five major measures of functional variation in plant metabolomes (Walker et al. 2023). To do that we used the predicted SMILES to query the Chemistry Development Kit (Willighagen et al. 2017) using the R package “rcdk” (Guha 2007) and calculated (i) the number of atoms in the largest conjugated π‐system (nAtomP), which is high in pigments and light‐absorbing molecules; (ii) hydrophobicity measured as the log‐ratio of solubility in octanol versus water (ALogP) (Ghose and Crippen 1986); (iii) the topological polar surface area, the sum of the surface area of polar atoms in Ångstroms (TopoPSA) (Ertl et al. 2000), which is negatively correlated with passive transport through cell membranes; (iv) the fraction of carbon atoms with sp_3_ electron orbits (i.e., only single bonds) to the total number of carbon atoms in the molecule (Fsp3), which is positively correlated with melting point, solubility, and the likelihood of a compound to exhibit bioactivity in pharmaceutical assays (Lovering et al. 2009); and (v) molecular weight (MW) in Daltons (see Table S3 for more detail). We used the chemical properties and concentrations of individual metabolites in our samples to calculate Community Weighted Means (CWMs) calculated in “BAT” package 2.10.0 (Cardoso et al. 2025) to quantify these five properties at a sample level (Table S4).

Lastly, we quantified three different measures of chemical β‐diversity between all plant individuals as dissimilarity matrices. First, we calculated simple chemical β‐diversity based on the presence and concentration of metabolites using Bray–Curtis dissimilarity in R package “vegan” (Oksanen et al. 2019). Second, we calculated structural chemical β‐diversity between the samples with Generalized UniFrac and “sampDis” functions, using the output generated in the “chemodiv” R‐package (Petrén et al. 2023) when calculating structural α‐diversity. Finally, we calculated functional chemical β‐diversity between the samples based on the CWMs of the five chemical properties using Bray–Curtis index. Each of these three approaches generated distance matrices that we used in subsequent statistical analyses.

### 2.3 Climatic Data Extraction

We extracted climatic variables from the CHELSA dataset (version 2.1; Karger et al. 2017; http://chelsa-climate.org), using data from the directory *GLOBAL/climatologies/1981‐2010/bio*. For each plant individual, climatic values were obtained based on its GPS coordinates in QGIS Desktop 3.30.0 (https://www.qgis.org/). We focused on nine variables with documented strong influence on plant physiology and distribution: the annual precipitation amount (bio12), annual range of air temperature (bio7), frost change frequency (fcf), growing season length TREELIM (gsl), mean annual air temperature (bio1), mean monthly climate moisture index (cmi_mean), number of growing degree days (ngd0), precipitation seasonality (bio15), and snow cover days (scd) (Tables S5, S6).

### 2.4 Soil Sampling and Analysis

We took two samples from opposite sides of the stem of each plant. We removed the organic layer and used Kopecky rings (5 × 5 cm) to take samples from 0 to 5 cm of the topsoil. We did not take samples from deeper layers as this was not feasible at most of the sites due to the shallow rocky soils. We mixed the two soil samples collected for each individual and dried them at 60°C for 24 h. We then homogenised the samples before passing them through an analytical sieve with 2 mm mesh. We used 20 g of the processed soil to analyse soil chemistry and other properties. We extracted total phosphorus using a mineralization method with two repetitions, and available phosphorus using the Mehlich 3 solution. We took all subsequent measurements using the Agilent ICP‐OES 5900 (Santa Clara, USA). For pH and conductivity, we extracted the soil sample using deionized water and measured it using the pH meter Mettler Toledo Seven‐Compact and conductivity meter Mettler Toledo Seven2Go (Schwerzenbach, Switzerland). For determination of total carbon and total nitrogen, we finely ground our samples using a ball mill (Retsch MM400), weighed them into tin capsules (Elemental Microanalysis, 8 × 5 mm) and analysed them on elemental analyser with a thermal conductivity detector (Flash Smart, Thermo Fisher Scientific, Bremen, Germany) (Table S7).

### 2.5 Statistical Analysis

First, we used Principal Component Analysis (PCA) to visualize the genetic variation in each species and to explore whether plants growing at different elevations form distinct groups based on their repetitive sequences (see Appendix S1). We then used partial Redundancy Analysis (pRDA) to test if the variation was explained by elevation. We used plant individuals as samples, elevation as explanatory variable, the standardized reads of the repeat clusters as response variables, and valley (Mölltal or Ködnitz) as covariate. We tested the significance of elevation with a Monte‐Carlo test with 9999 permutations in Canoco 5 (ter Braak and Smilauer 2012).

Before testing our hypotheses, we also derived composite variables that summarized the climatic and soil conditions measured for each plant. To achieve this, we performed two PCAs in Canoco 5 (ter Braak and Smilauer 2012). In these analyses, we used plant individuals as samples, while either the climatic or soil variables were included as response variables. We then extracted each plant's score on the first PCA axis to generate synthetic variables representing overall climatic or soil conditions (Figure S1). We inverted the axes so that higher values corresponded to harsher environments (i.e., high‐elevation climates and low nutrient availability) ensuring consistency with other analyses where higher values of elevation and herbivory likewise denoted more stressful conditions (Table S8). We explored the correlation between elevation, climate, soil conditions, and herbivory using Pearson correlation coefficients. Additionally, we examined how variation in climate, soil, and herbivory experienced by individual plants changed with elevation. Individuals were assigned to 200‐m elevation bands (900–1099 m, 1100–1299 m, etc.). For each elevation band, we calculated the variance of residuals from linear models including species and valley as covariates, thereby accounting for their effects. Residual variances were log‐transformed, and linear models were used to test for trends in variance along elevation.

To test our first hypothesis, we used partial Redundancy Analysis (pRDA) to explore how changes in concentration, richness, and structural α‐diversity of metabolites correlate with elevation, climate, soil conditions, and herbivory experienced by the plants. We used plant individuals as samples, log‐transformed concentration, richness, and structural α‐diversity of their metabolites as response variables, and valley (Mölltal or Ködnitz) and plant species as covariates. We used climate (PCA axis 1), soil conditions (PCA axis 1), and herbivory damage (percentage of damaged leaf area) as explanatory variables and tested their effect on the chemistry variables with a Monte‐Carlo test with 9,999 permutations in Canoco 5 (ter Braak and Smilauer 2012). Elevation (m a.s.l.) was used as supplementary variable, not constraining the ordination space. We then performed variance partitioning to quantify the amount of variation in the chemistry data uniquely explained by each of the selected variables. To test our second hypothesis, we used the same approach to explore how elevation, climate, soil conditions, and herbivory correlate with chemical properties representing functional chemical traits of individual plant samples.

To test our third hypothesis, we first examined how simple, functional, and structural chemical β‐diversity vary with elevation. Individuals were assigned to 200‐m elevation bands (900–1099, 1100–1299 m, etc.). For each plant, we calculated the mean pairwise dissimilarity to its neighbours within the same elevation band for each of the three measures of chemical β‐diversity. We then used linear models (LMs) to test for correlations between elevation and mean pairwise distances, including species identity and valley as covariates in the null model. We compared null models with models including elevation using AIC. To assess potential non‐linear relationships, we fitted generalized additive models (GAMs) with the “mgcv” R‐package (Wood 2011), using the same response variables, predictors, and covariates as in the LMs. We evaluated monotonic trends in the GAMs with the Jonckheere–Terpstra trend test implemented in the “clinfun” R‐package (Seshan 2023) and compared GAMs with and without elevation effects using the significance of smooth terms. All LM and GAM p‐values were corrected for multiple testing using the false discovery rate (FDR) (Benjamini and Hochberg 1995). To determine whether elevational trends reflected intraspecific variation, we repeated the LM and GAM analyses using only pairwise distances among conspecific individuals.

To further explore which factors drive changes in chemical β‐diversity along elevational gradients, we tested how the three types of chemical β‐diversity correlate with climate, soil, and herbivory. For each explanatory variable (climate PC1, soil PC1, herbivory), plants were divided into quartiles, and individuals from the bottom and top quartiles were extracted. We calculated the mean pairwise dissimilarities for each plant relative to others in the same quartile. We then used analysis of covariance (ANCOVA) to test whether mean pairwise dissimilarities among plants within a quartile differed between the bottom and top quartiles, including plant species identity and valley as covariates. Resulting p‐values were corrected for multiple testing using FDR.

## 3 Results

### 3.1 Plants and Background Data

Out of the 157 plants we analysed, we identified 45 as *S. appendiculata*, 16 as *S. caprea*, 67 as *S. mielichhoferi*, and 29 as *S. myrsinifolia*. Three individuals were identified as tentative *S. appendiculata × S. caprea* hybrids and were excluded from our further analyses (see Appendix S1). Most species did not form distinct elevational groups, as individuals from different elevations were intermixed based on their repetitive sequences (Figure 1). The clearest elevational separation was observed in *S. mielichhoferi*, where individuals from the lowest elevations grouped largely apart from those from the highest elevations. This was confirmed with RDA where elevation explained 2.27% of the adjusted variation of repeat cluster reads for *S. appendiculata* (pseudo‐*F* = 2.0, *p* = 0.004), < 0.01% of the adjusted variation for *S. caprea* (pseudo‐*F* = 0.8, *p* = 0.903), 6.17% of the adjusted variation for *S. mielichhoferi* (pseudo‐*F* = 5.3, *p* < 0.001) and 0.46% of the variation for *S. myrsinifolia* (pseudo‐*F* = 1.1, *p* = 0.147).

We detected a total of 1478 unique *m*/*z* features (which we refer to as metabolites), out of which 1084 had their SMILES predicted. Among these, 68 were classified as flavonoids, 77 as salicinoids and their analogues and 81 as primary metabolites.

Pearson correlation coefficients were 0.96 between climate conditions and elevation, 0.13 between soil conditions and elevation, and 0.43 between herbivory and elevation. When testing whether inter‐individual variation in climate, soil, and herbivory changed along the elevational gradient using linear models, we found a significant increase in herbivory variation with elevation (*F* = 22.86, *p* = 0.005). In contrast, variation in climate (*F* = 0.39, *p* = 0.562) and soil (*F* = 5.72, *p* = 0.062) did not show significant elevational trends (Figure S2).

### 3.2 Concentration, Richness, and Structural α‐Diversity

When testing our first hypothesis on metabolite concentration richness and structural α‐diversity, the full pRDA model including the effects of climate, soil conditions, and herbivory explained 5.82% of the adjusted variation (pseudo‐*F* = 4.1, *p* < 0.001). Harsher climate and soil conditions were negatively correlated with richness of flavonoids and structural α‐diversity of all metabolites, flavonoids and primary metabolites but positively correlated with the concentration of all metabolites, salicinoids and primary metabolites. Herbivory was negatively correlated with the concentration of flavonoids. The rest of the chemical traits showed only limited correlations with the explanatory variables (Figure 2). When we performed variance partitioning, we recovered significant unique effects of climate (pseudo‐*F* = 8.8, *p* < 0.001). Unique effects of soil conditions (pseudo‐*F* = 0.2, *p* = 0.994) and herbivory (pseudo‐*F* = 2.1, *p* = 0.632) were not significant.

**Figure 2:**
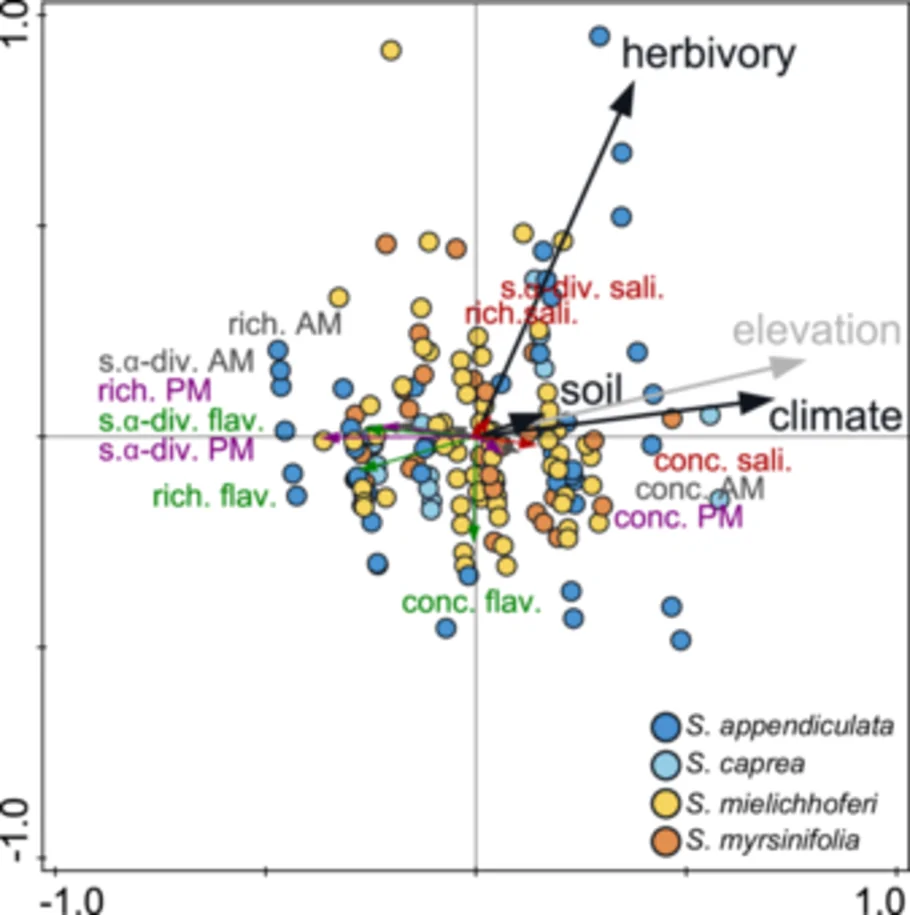
Variation in concentration, richness and structural α‐diversity of metabolites among studied *Salix* plants as explained with climatic conditions, soil parameters and herbivory and analysed with partial Redundancy Analysis (pRDA) using valley (Mölltal or Ködnitz) and *Salix* species as covariates and elevation as supplementary variable (grey). Climatic conditions, soil parameters and herbivory account for 7.68% of the variation (5.82% adjusted, pseudo‐*F* = 4.1, *p* < 0.001). Plant individuals appear as circles and explanatory variables appear as black arrows. [Color figure can be viewed at wileyonlinelibrary.com]

### 3.3 Chemical Properties

When testing our second hypothesis that chemical properties of metabolites will respond strongly and specifically to climate conditions, soil conditions, and herbivory, the full pRDA model including the effects of climate, soil and herbivory explained 5.71% of the adjusted variation in metabolite chemical properties (pseudo‐*F* = 4.1, *p* < 0.001). Climate was negatively correlated with conjugated π‐system (nAtomP), hydrophobicity (AlogP) and proportion of sp3‐hybridized carbon atoms in the molecule (Fsp3) of salicinoids, molecular weight (MW), topological polar surface area (TopoPSA) and AlogP of flavonoids, and Fsp3 of all metabolites. Additionally, climate was positively correlated with AlogP of all metabolites, herbivory was positively correlated with nAtomP of flavonoids, and soil was positively correlated with Fsp3 of flavonoids (Figure 3). Chemical properties of primary metabolites generally showed weaker correlations with the three explanatory variables than those of flavonoids or salicinoids. When we performed variance partitioning, we recovered significant unique effects of climate (pseudo‐*F* = 6.8, *p* < 0.001). Unique effects of soil conditions (pseudo‐*F* = 0.5, *p* = 0.851) and herbivory (pseudo‐*F* = 1.1, *p* = 0.333) were not significant.

**Figure 3:**
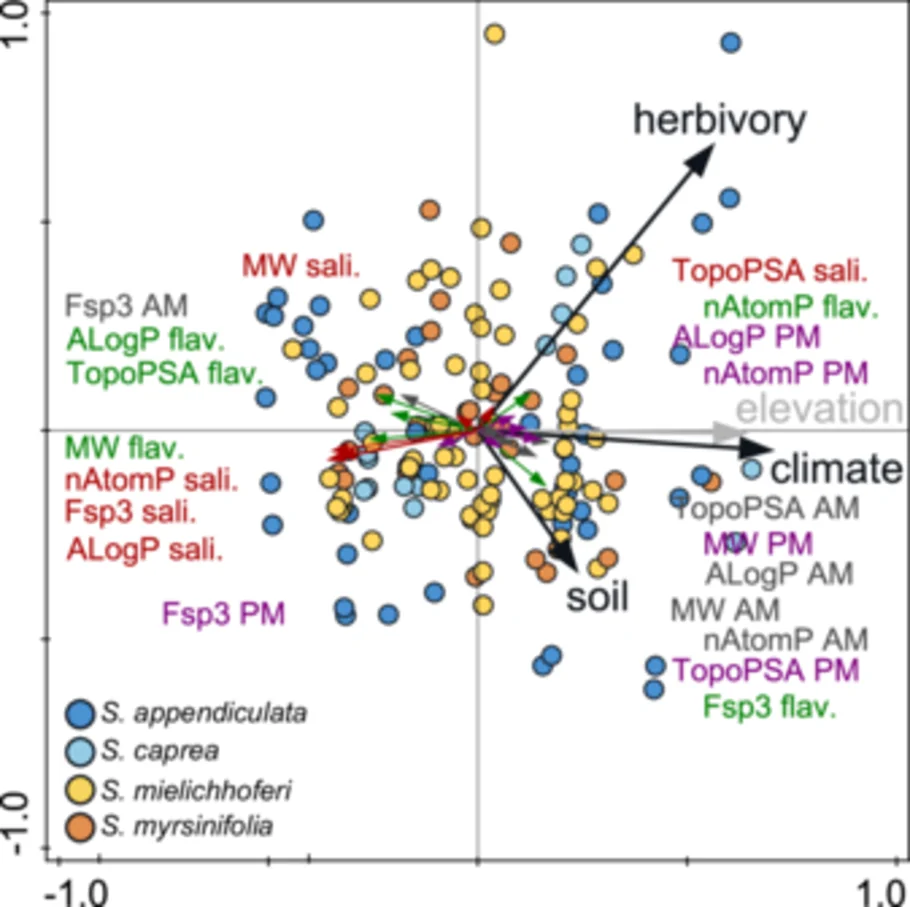
Variation in chemical proprieties among studied *Salix* plants as explained with climatic conditions, soil parameters and herbivory and analysed with partial Redundancy Analysis (pRDA) using valley (Mölltal or Ködnitz) and *Salix* species as covariates and elevation as supplementary variable (grey). Climatic conditions, soil parameters and herbivory account for 7.57% of the variation (5.71% adjusted, pseudo‐*F* = 4.1, *p* < 0.001). Plant individuals appear as circles and explanatory variables appear as black arrows. [Color figure can be viewed at wileyonlinelibrary.com]

### 3.4 Chemical β‐Diversity

When testing our third hypothesis on changes in β‐diversity, most of its measures across all four metabolites datasets showed a linear increase with elevation except for functional and structural β‐diversity of salicinoids (Figure 4). The intraspecific β‐diversity showed similar but weaker trends, with functional β‐diversity of all metabolites and structural β‐diversity of flavonoids being no longer significantly correlated with elevation (Figure 5). The significant trends identified by the LMs were generally confirmed by the GAMs, which captured these patterns, sometimes in more complex nonlinear forms. Moreover, the GAMs revealed additional significant trends in both functional (*F*_(4.56,147)_ = 3.04, *p* = 0.007) and structural (*F*_(5.46,147)_ = 5.72, *p* < 0.001) β‐diversity of salicinoids, which were lowest at middle of our gradient (Figure 4). At the intraspecific level, GAM revealed similar significant trends also in the case of functional β‐diversity of salicinoids (*F*_(4.1,147)_ = 2.62, *p* = 0.021), structural β‐diversity of flavonoids (*F*_(5.71,145)_ = 5.56, *p* < 0.001) and structural β‐diversity of salicinoids (*F*_(4.94,146)_ = 3.92, *p* < 0.001) (Figure 5 and Table S9).

**Figure 4:**
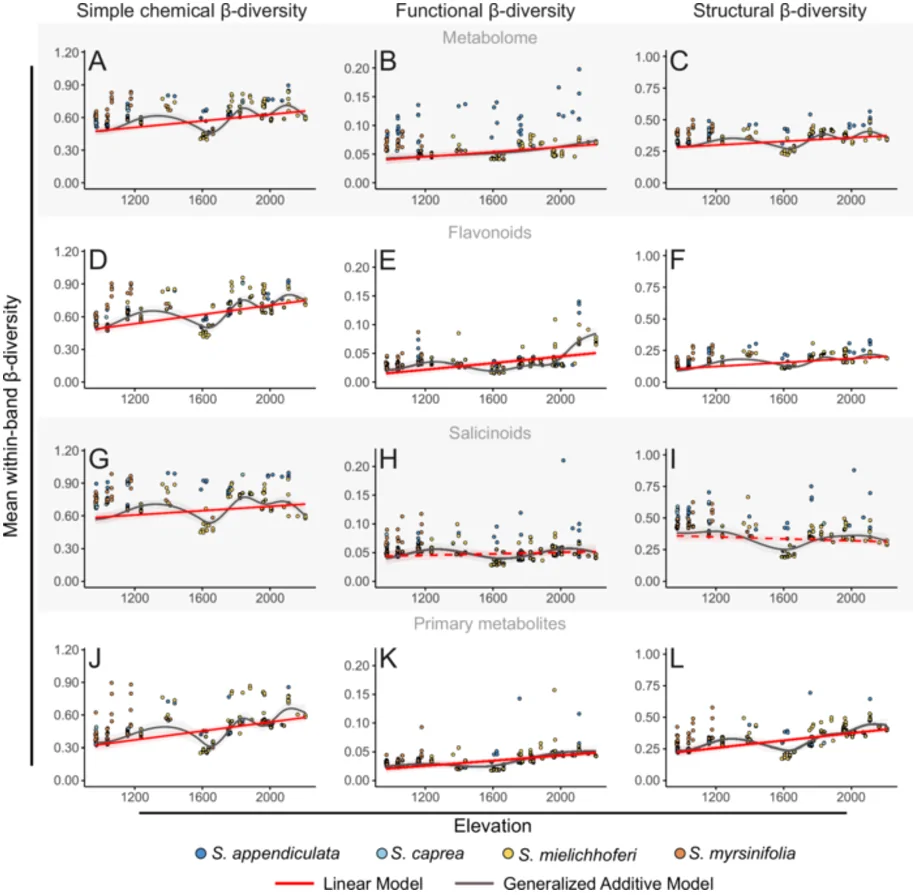
Correlation between elevation, simple β‐diversity of all metabolites (A), flavonoids (D), salicinoids (G) and primary metabolites (J), functional β‐diversity of all metabolites (B), flavonoids (E), salicinoids (H) and primary metabolites (K), and structural β‐diversity of all metabolites (C), flavonoids (F), salicinoids (I) and primary metabolites (L). Relationships were fitted using Linear Models (LMs, red) and Generalized Additive Models (GAMs, black). Significant trends are shown with solid lines, while non‐significant relationships are represented by dashed lines. Symbol colours indicate *Salix* species. [Color figure can be viewed at wileyonlinelibrary.com]

**Figure 5:**
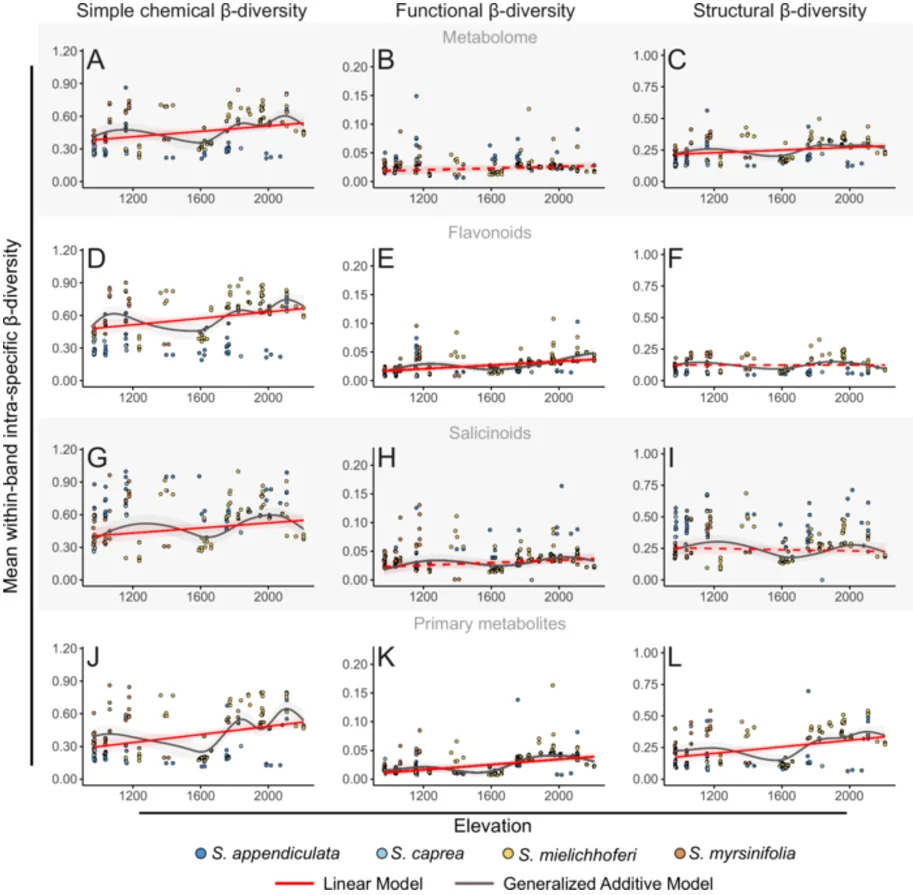
Correlation between elevation, intraspecific simple β‐diversity of all metabolites (A), flavonoids (D), salicinoids (G) and primary metabolites (J), intraspecific functional β‐diversity of all metabolites (B), flavonoids (E), salicinoids (H) and primary metabolites (K) and intraspecific structural β‐diversity of all metabolites (C), flavonoids (F), salicinoids (I) and primary metabolites (L). Relationships were fitted using Linear Models (LMs, red) and Generalized Additive Models (GAMs, black). Significant trends are shown with solid lines, while non‐significant relationships are represented by dashed lines. Symbol colours indicate *Salix* species. [Color figure can be viewed at wileyonlinelibrary.com]

The ANCOVA comparing individuals in the bottom and top quartiles based on climatic conditions, soil conditions, and herbivory revealed that plants exposed to harsher climatic conditions generally exhibited higher chemical β‐diversity. The two exceptions were the functional β‐diversity of all metabolites, which was lower in harsher climates (*F*_(1,75)_ = 86.37, *p* < 0.001), and structural β‐diversity of salicinoids, which showed no significant difference (*F*_(1,75)_ = 0.97, *p* = 0.328) (Figure 6). Plants growing in soils with lower total nutrient content, reduced conductivity, and higher pH demonstrated significantly lower simple (*F*_(1,74)_ = 11.58, *p* = 0.002), functional (*F*_(1,74)_ = 9.92, *p* = 0.004), and structural β‐diversity (*F*_(1,74)_ = 8.00, *p* = 0.010) of salicinoids, lower functional β‐diversity of primary metabolites (*F*_(1,74)_ = 9.68, *p* = 0.003) and higher simple (*F*_(1,74)_ = 21.36, *p* < 0.001) and structural β‐diversity (*F*_(1,74)_ = 5.21, *p* = 0.025) of primary metabolites compared to plants in more nutrient‐rich soils (Figure 7). Plants experiencing the most herbivory showed higher functional β‐diversity of all metabolites (*F*_(1,74)_ = 6.95, *p* = 0.010), lower structural β‐diversity of salicinoids (*F*_(1,74)_ = 13.90, *p* < 0.001) and higher functional β‐diversity of primary metabolites (*F*_(1,74) _= 5.02, *p* = 0.028) (Figure 8). Other quartile comparisons were not statistically significant (Figures 6, 7, 8, full ANCOVA results in Table S10).

**Figure 6:**
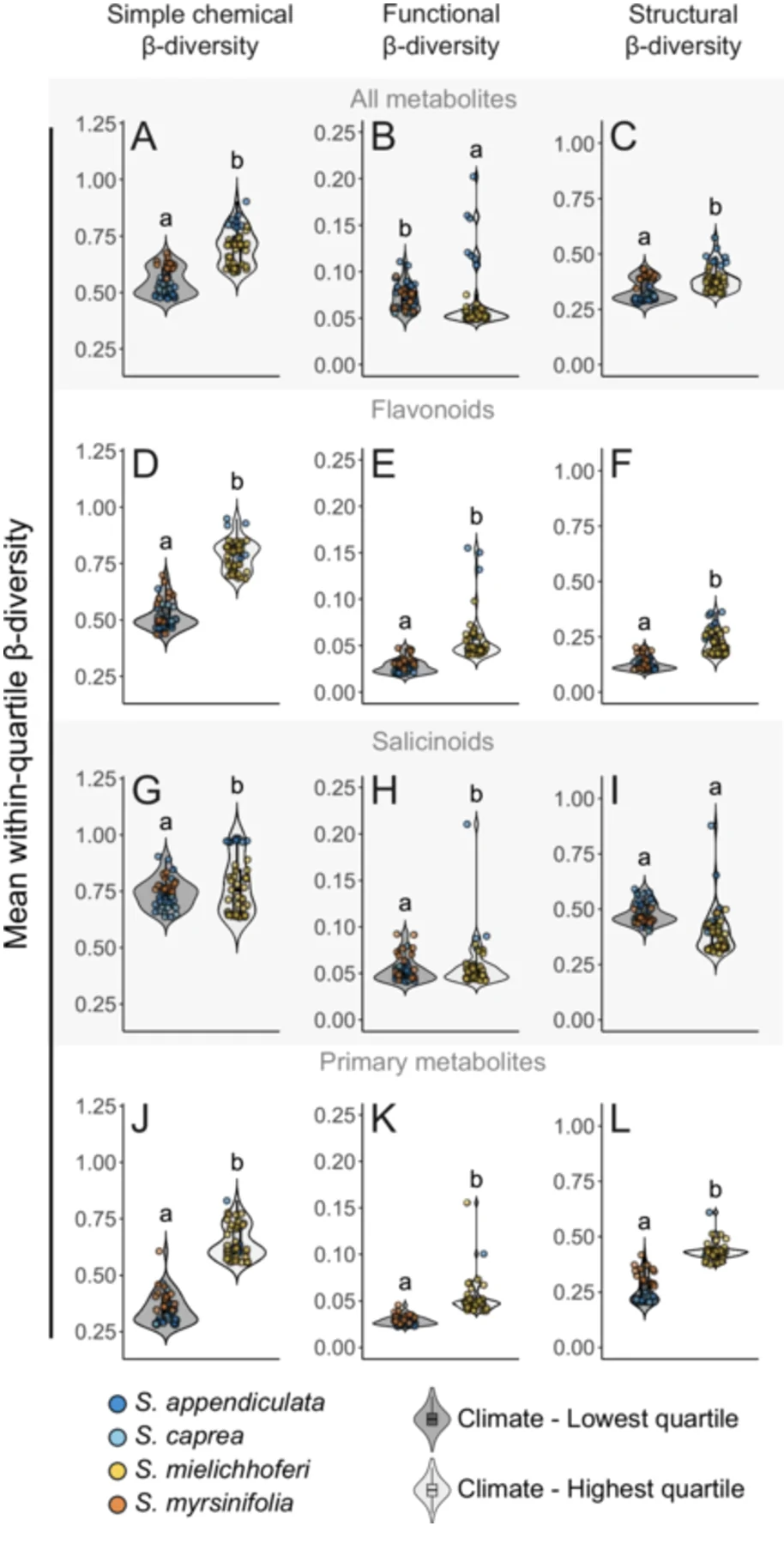
Comparison of simple β‐diversity of all metabolites (A), flavonoids (D), salicinoids (G) and primary metabolites (J), functional β‐diversity of all metabolites (B), flavonoids (E), salicinoids (H) and primary metabolites (K), and structural β‐diversity of all metabolites (C), flavonoids (F), salicinoids (I) and primary metabolites (L) between individuals from the lowest (mild) and highest (harsh) quartiles of climatic conditions (PCA axis 1). The letters indicate significantly different quartiles for mean β‐diversity. Individual tree means are shown as dots. The violins show the distribution of individual mean β‐diversity within each quartile, and the boxplots summarize this distribution, with horizontal lines marking the medians. Symbol colours indicate *Salix* species. [Color figure can be viewed at wileyonlinelibrary.com]

**Figure 7:**
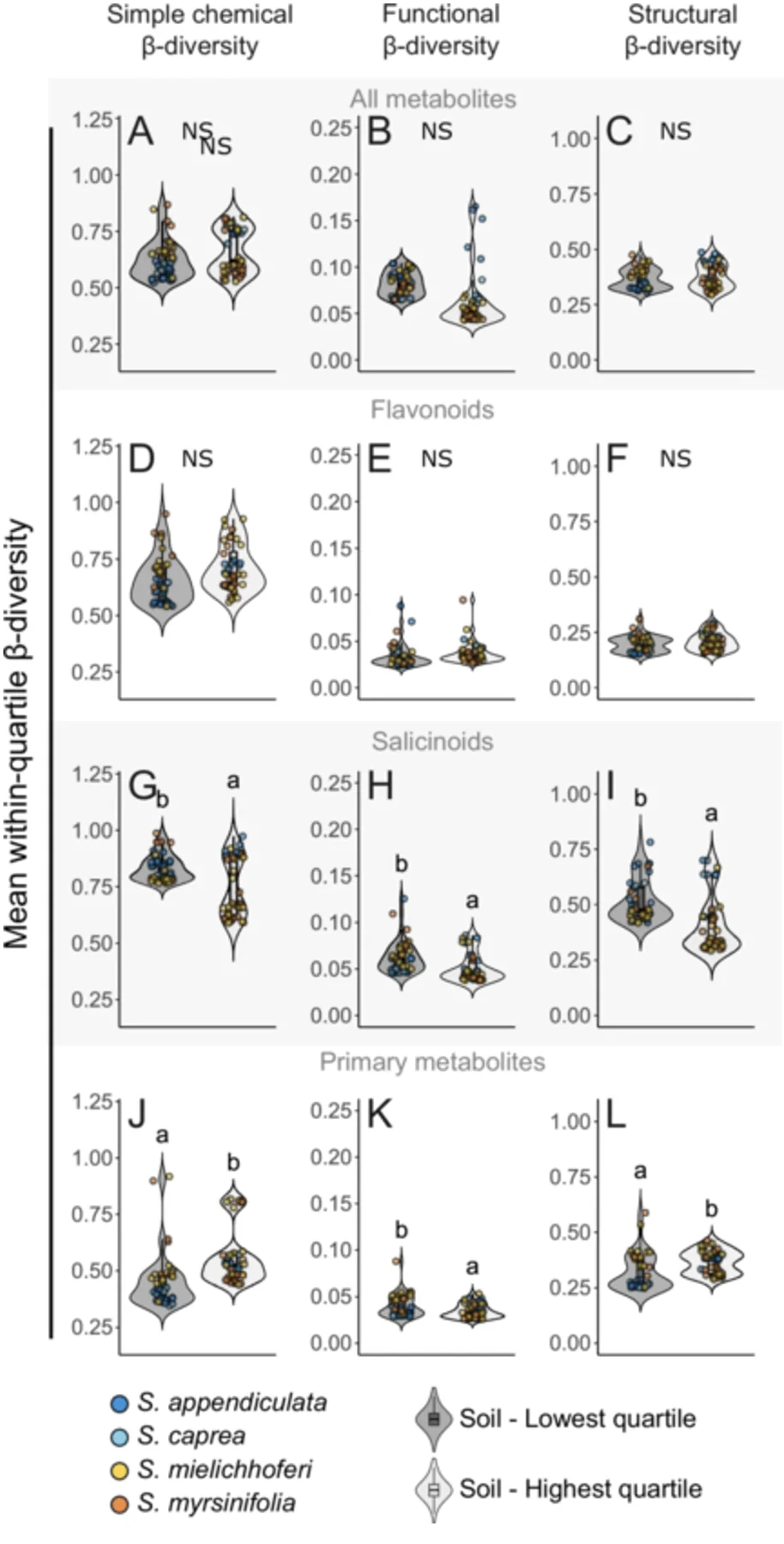
Comparison of simple β‐diversity of all metabolites (A), flavonoids (D), salicinoids (G) and primary metabolites (J), functional β‐diversity of all metabolites (B), flavonoids (E), salicinoids (H) and primary metabolites (K), and structural β‐diversity of all metabolites (C), flavonoids (F), salicinoids (I) and primary metabolites (L) between individuals from the lowest (rich) and highest (poor) quartiles of soil conditions (PCA axis 1). The letters indicate significantly different quartiles for mean β‐diversity. Individual tree means are shown as dots. The violins show the distribution of individual mean β‐diversity within each quartile, and the boxplots summarize this distribution, with horizontal lines marking the medians. Symbol colours indicate *Salix* species. [Color figure can be viewed at wileyonlinelibrary.com]

**Figure 8:**
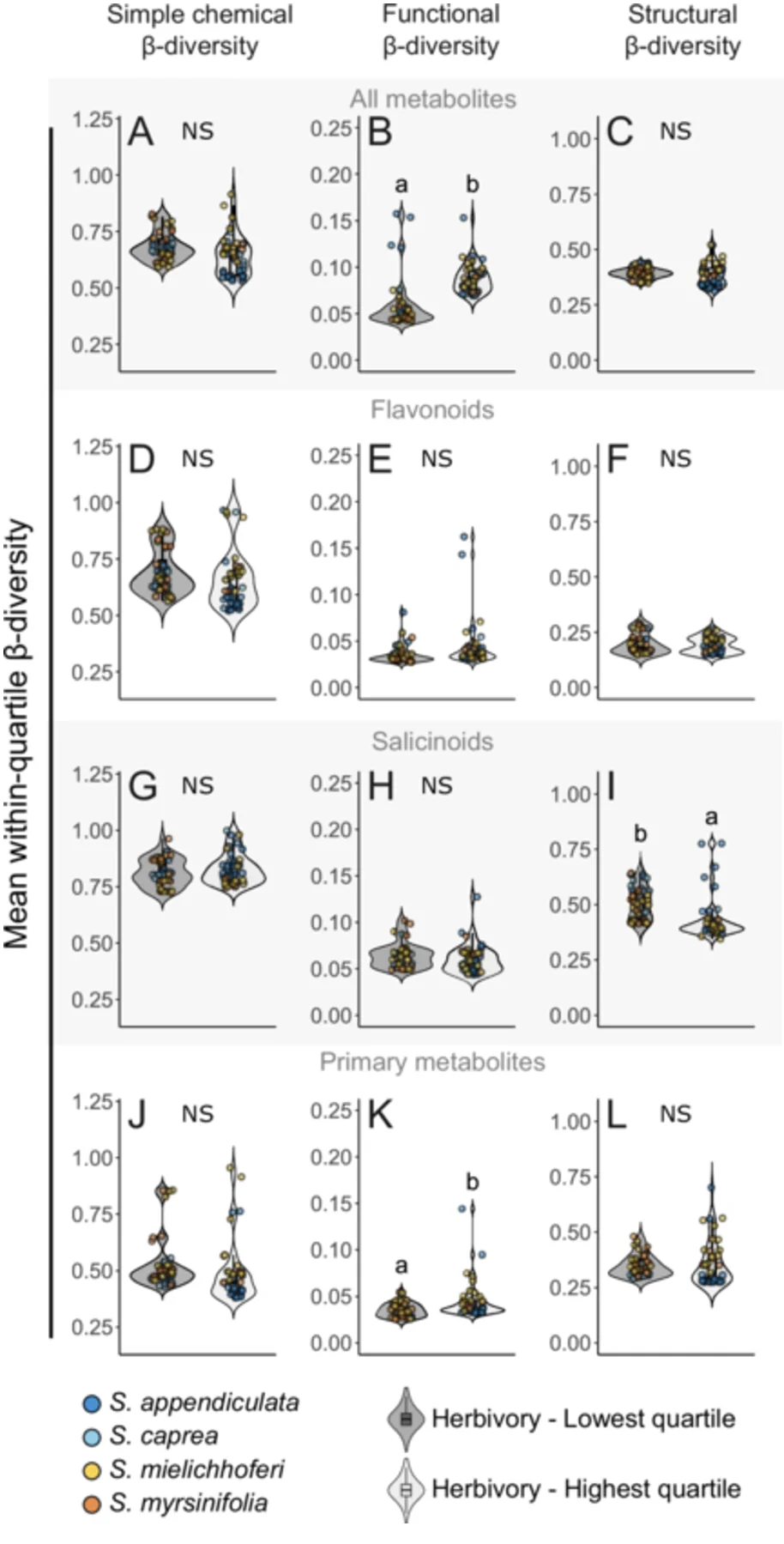
Comparison of simple β‐diversity of all metabolites (A), flavonoids (D), salicinoids (G) and primary metabolites (J), functional β‐diversity of all metabolites (B), flavonoids (E), salicinoids (H) and primary metabolites (K), and structural β‐diversity of all metabolites (C), flavonoids (F), salicinoids (I) and primary metabolites (L) between individuals from the lowest and highest quartiles of herbivory (%). The letters indicate significantly different quartiles for mean β‐diversity. Individual tree means are shown as dots. The violins show the distribution of individual mean β‐diversity within each quartile, and the boxplots summarize this distribution, with horizontal lines marking the medians. Symbol colours indicate *Salix* species. [Color figure can be viewed at wileyonlinelibrary.com]

## 4 Discussion

In this study, we examined how multiple measures of willow metabolomes change along an elevational gradient by considering climatic conditions, soil conditions, herbivory, primary chemistry, and defensive metabolites and their functionally relevant chemical properties. This broader framework allowed us to move beyond the defence‐centred interpretation of elevational metabolomic shifts and to show that elevational shifts in willow chemistry are not restricted to defence‐related metabolites but extend across broader components of the metabolome. Given the weak genetic structure we recorded, these elevational changes appear to be more consistent with environmentally associated responses than with pronounced genetic differentiation among populations across elevation.

Plants growing at higher elevations and in harsher climatic conditions showed higher metabolite concentrations, especially of salicinoids, but lower flavonoid richness and lower structural α‐diversity of the overall metabolome. Considered together, these patterns suggest a shift towards stronger accumulation of a narrower set of compounds under stressful conditions. Such responses may be particularly pronounced for flavonoids, which are often linked to antioxidant protection and UV screening under environmental stress (Tegelberg and Julkunen‐Tiitto 2001; Zhao et al. 2025). Overall, these results are broadly consistent with the Resource Availability Hypothesis (Coley et al. 1985) and with previous studies that interpreted elevational increases in metabolite concentrations mainly in terms of defence and protection against abiotic stress (Defossez et al. 2018; Moreira et al. 2018; Sam et al. 2019; Volf et al. 2022). In our study, herbivory damage increased with elevation, which may partly reinforce this interpretation, but its overall association with metabolite concentration and α‐diversity remained weak.

At the same time, the recovered trends in primary metabolites indicate that these patterns are not restricted to specialized defence chemistry. Similar coupled shifts in primary and specialized metabolites have been reported along elevational gradients in lodgepole pine, and broader metabolic reorganization has also been documented in other alpine plants exposed to environmental change (Mullin et al. 2021; Wu et al. 2025). More generally, changes in primary metabolism under stressful conditions may reflect physiological adjustment linked to carbon allocation, resource limitation, and stress acclimation rather than defence alone (Pandey et al. 2019; Xin et al. 2019). This interpretation does not exclude an adaptive role of specialized metabolites in defence, but it indicates that elevational metabolomic shifts should not be understood solely as changes in defensive investment. Rather, they likely reflect coordinated adjustments across multiple components of the metabolome, some of which may support defence directly, while others are linked more broadly to physiological acclimation and resource allocation.

The most pronounced changes in chemical properties occurred in flavonoids and salicinoids whereas responses in the whole metabolome and in primary metabolites were generally weaker. This pattern may indicate that chemical properties are more responsive in specialized metabolites linked to stress responses than in broader components of the metabolome, possibly because primary metabolism needs to remain comparatively stable to maintain metabolic homeostasis and essential cellular functions (Obata and Fernie 2012). It is also consistent with the view that high‐altitude responses involve coordinated, but not uniform, changes across different functional levels of plant organisation, from metabolites to broader physiological traits (Walker et al. 2023; Kumari and Kumar 2024; Chadwick et al. 2025). Among the strongest patterns, flavonoids at higher elevations and in harsher climates tended to have lower molecular weight, while both flavonoids and salicinoids showed lower hydrophobicity under harsher climatic conditions. Some of these shifts may be functionally relevant for abiotic stress responses, including protection from UV radiation or water balance, but direct assays will be needed to test these interpretations (Tegelberg and Julkunen‐Tiitto 2001; Haghpanah et al. 2024). At the same time, our results remain correlative and do not demonstrate that chemical properties provide a more robust functional signal than other measures of phytochemical diversity. Nevertheless, they identify chemical properties as a promising axis for future work linking metabolome structure to ecological function, particularly for testing whether they can help distinguish specialized stress‐related shifts from broader metabolomic changes.

Previous studies have suggested that harsh abiotic conditions and limited resources should filter plant communities toward greater chemical similarity, whereas plants in more resource‐rich environments may show greater chemical variation (Defossez et al. 2021; Volf et al. 2023; Chadwick et al. 2025; Nomoto et al. 2025a; Nomoto et al. 2025b). In our study, support for this expectation was clearest in salicinoids, whose simple, structural, and functional β‐diversity were all lower in nutrient‐poorer soils. Herbivory damage showed much weaker effects with structural β‐diversity of salicinoids even decreasing in plants experiencing higher herbivory. These results further highlight the importance of soil resource conditions for shaping structural variation in willow specialized metabolites, consistent with evidence from lowland species where structural variation in metabolomes was associated with soil nitrogen (Renoult et al. 2025). This interpretation is further supported by the higher simple and structural β‐diversity of primary metabolites in nutrient‐poor soils, which may indicate that resource limitation is also associated with broader changes in metabolic regulation, potentially involving shifts in carbon–nutrient balance and related physiological processes (Pandey et al. 2019; Xin et al. 2019).

Contrary to our expectations, most measures of chemical β‐diversity increased rather than decreased along the elevational gradient, and this trend was often also detectable at the intraspecific level. This result differs from studies conducted at broader community and macroevolutionary scales, where harsher abiotic conditions were associated with lower chemical β‐diversity (Volf et al. 2023; Chadwick et al. 2025; Nomoto et al. 2025a), but is in line with our earlier analysis of alpine willows at the intraspecific level, which also revealed increasing chemical β‐diversity with elevation (Volf et al. 2022). Together, these comparisons suggest that the direction of elevational trends in chemical β‐diversity may depend on the ecological scale considered. One possible explanation is that increasing chemical β‐diversity corresponds to the elevational increase in variability of herbivory damage recorded in the present study. More variable and less predictable herbivory may favour greater variation in defensive chemistry among plants and, in some cases, may be more important than absolute herbivory levels (Pearse et al. 2018; Wetzel et al. 2023). In addition, although our metabolomic analyses were performed on intact leaves, differences in systemic induced responses among plants experiencing different herbivory levels may also contribute to the local‐scale patterns observed here. At the same time, because increased β‐diversity was also evident in primary metabolites, this pattern is unlikely to reflect variation in defence chemistry alone, similarly to the case of concentration and α‐diversity. It may instead suggest a broader association between harsher climatic conditions and greater variation in plant metabolic responses, consistent with the stronger predictive power of climate than of other variables in our models. This pattern could partly reflect greater stochasticity in metabolic responses under climatic stress, although the underlying mechanisms remain unresolved (Zandalinas et al. 2022). However, these explanations remain speculative and should be treated as hypotheses for future work rather than as conclusions from the present data.

Most species in our study did not form pronounced elevational groups based on their repetitive sequences although willow species can show clear genetic differentiation across broader geographic areas (Kosiński et al. 2019; Wagner et al. 2021; Pittet et al. 2025a; Pittet et al. 2025b). At the local scale studied here, the weak elevational structure likely reflects the high dispersal ability of willows and their capacity to rapidly colonize newly suitable habitats (Boulanger‐Lapointe et al. 2014; Gramlich et al. 2016). This may maintain gene flow across the gradient and limit the development of strong elevational genetic structure. The observed metabolomic shifts are therefore unlikely to be explained primarily by strong genetic divergence among populations in most of the studied species and may instead reflect phenotypic plasticity, although this interpretation should remain cautious because it is based on indirect evidence. A meta‐analysis of Salicaceae found both environmentally and genetically determined variation in chemical defence compounds, and substantial plasticity across sites (Barker et al. 2019). Our results differ from studies on other alpine plants, including herbaceous species, that have reported clearer signatures of local adaptation along elevational gradients (de Villemereuil et al. 2018; Bakhtiari et al. 2019). The partial separation of low‐ and high‐elevation individuals in *S. mielichhoferi* indicates that genetic differentiation may still contribute to elevational variation in some taxa, but this pattern was relatively weak and absent in the other species. Reciprocal transplant experiments combined with genomic analyses are needed to test more directly to what extent elevational shifts in willow chemistry reflect phenotypic plasticity versus local adaptation. Given projected climatic changes by the end of the century, resulting in warmer conditions, longer growing seasons, and reduced snow cover (Karger et al. 2017), such plasticity would likely facilitate the persistence of these willows across the studied gradient, especially in species whose current elevational ranges already exceed the magnitude of the projected climatic shift. At the same time, high‐elevation specialists may be more vulnerable to climate change and may also be threatened by habitat transformation associated with the upward encroachment of related *Salix* shrubs from lower elevations, including some of those studied here (Cannone et al. 2022).

In conclusion, our study extends previous work on elevational phytochemical variation in willows by showing that these patterns cannot be interpreted only through changes in specialized defence chemistry. We show that elevational shifts in willow metabolomes reflect a broader reorganization of plant metabolism under climatic and edaphic stress. Climate emerged as the main correlate of increasing metabolite concentrations and declining α‐diversity, whereas soil conditions helped explain variation in β‐diversity, particularly in salicinoids and primary metabolites. Chemical properties further revealed that the strongest stress‐associated shifts were concentrated in specialized metabolites rather than across the metabolome as a whole, highlighting their potential for linking metabolomic traits to ecological function (Walker et al. 2023). While our results suggest weak genetic differences between willow populations from different elevations, future experimental work is needed to better disentangle the relative roles of adaptive evolution and phenotypic plasticity in shaping willow metabolomes along elevational gradients. Furthermore, evidence from polyploid systems indicates that whole‐genome duplications can substantially alter plant chemical diversity, and many polyploid lineages are associated with environmentally harsh or fluctuating habitats (Van de Peer et al. 2021; Tossi et al. 2022). Because our study included only two diploid and two polyploid species, the dataset was insufficient for robust statistical comparisons on the ploidy level. Experimental studies combined with predictive modelling will be essential for forecasting how changing climate regimes will shape plant metabolomes and the ecological interactions they mediate, and for identifying the chemical and genetic traits that enable plants to persist under future environmental conditions.

## Conflicts of Interest

The authors declare no conflicts of interest.

## Supporting information


Supporting File 1

Supporting File 2

## Data Availability

All the supplementary files and all the data to support the findings of this study are openly available on Zenodo at https://doi.org/10.5281/zenodo.21792016).

## References

[pce70775-bib-0001] Abràmoff, M. D. , P. J. Magalhães , and S. J. Ram . 2004. “Image Processing With ImageJ.” Biophotonics International 11: 36–42.

[pce70775-bib-0002] Bakhtiari, M. , L. Formenti , V. Caggìa , G. Glauser , and S. Rasmann . 2019. “Variable Effects on Growth and Defense Traits for Plant Ecotypic Differentiation and Phenotypic Plasticity Along Elevation Gradients.” Ecology and Evolution 9: 3740–3755. 10.1002/ece3.4999 PMC6468067 31015963

[pce70775-bib-0003] Bakhtiari, M. , G. Glauser , E. Defossez , and S. Rasmann . 2021. “Ecological Convergence of Secondary Phytochemicals Along Elevational Gradients.” New Phytologist 229: 1755–1767. 10.1111/nph.16966 32981048

[pce70775-bib-0004] Barker, H. L. , L. M. Holeski , and R. L. Lindroth . 2019. “Independent and Interactive Effects of Plant Genotype and Environment on Plant Traits and Insect Herbivore Performance: A Meta‐Analysis With Salicaceae.” Functional Ecology 33: 422–435.

[pce70775-bib-0005] Becerra, J. X. 2007. “The Impact of Herbivore‐Plant Coevolution on Plant Community Structure.” Proceedings of the National Academy of Sciences 104: 7483–7488. 10.1073/pnas.0608253104 PMC1855276 17456606

[pce70775-bib-0006] Benjamini, Y. , and Y. Hochberg . 1995. “Controlling the False Discovery Rate: A Practical and Powerful Approach to Multiple Testing.” Journal of the Royal Statistical Society Series B: Statistical Methodology 57: 289–300.

[pce70775-bib-0007] Bennett, R. N. , and R. M. Wallsgrove . 1994. “Secondary Metabolites in Plant Defence Mechanisms.” New Phytologist 127: 617–633. 10.1111/j.1469-8137.1994.tb02968.x 33874382

[pce70775-bib-0008] Boulanger‐Lapointe, N. , E. Lévesque , S. Boudreau , G. H. R. Henry , and N. M. Schmidt . 2014. “Population Structure and Dynamics of Arctic Willow (*Salix arctica*) in the High Arctic.” Journal of Biogeography 41: 1967–1978.

[pce70775-bib-0009] ter Braak, C. J. , and P. Smilauer . 2012. Canoco Reference Manual and User's Guide: Software for Ordination (version 5.0). Microcomputer power.

[pce70775-bib-0010] Cannone, N. , M. Guglielmin , C. Casiraghi , and F. Malfasi . 2022. “Salix Shrub Encroachment Along a 1000 m Elevation Gradient Triggers a Major Ecosystem Change in the European Alps.” Ecography 2022: e06007.

[pce70775-bib-0011] Cardoso, P. , S. Mammola , F. Rigal and J. C. Carvalho . 2025. “BAT: Biodiversity Assessment Tools.” R package version 2.10.0. https://CRAN.R-project.org/package=BAT.

[pce70775-bib-0012] Chadwick, S. E. , D. Henderson , D. L. Forrister , et al. 2025. “Chemical Properties of Foliar Metabolomes Represent a Key Axis of Functional Trait Variation in Forests of the Tropical Andes.” Proceedings of the Royal Society B: Biological Sciences 293: 20251721. 10.1098/rspb.2025.1721 41558779

[pce70775-bib-0013] Coley, P. D. , J. P. Bryant , and F. S. Chapin . 1985. “Resource Availability and Plant Antiherbivore Defense.” Science 230: 895–899. 10.1126/science.230.4728.895 17739203

[pce70775-bib-0014] Defossez, E. , L. Pellissier , and S. Rasmann . 2018. “The Unfolding of Plant Growth Form‐Defence Syndromes Along Elevation Gradients.” Ecology Letters 21: 609–618. 10.1111/ele.12926 29484833

[pce70775-bib-0015] Defossez, E. , C. Pitteloud , P. Descombes , et al. 2021. “Spatial and Evolutionary Predictability of Phytochemical Diversity.” Proceedings of the National Academy of Sciences 118: e2013344118. 10.1073/pnas.2013344118 PMC7826413 33431671

[pce70775-bib-0016] Dostálek, T. , M. B. Rokaya , P. Maršík , et al. 2016. “Trade‐Off Among Different Anti‐Herbivore Defence Strategies Along an Altitudinal Gradient.” AoB Plants 8: plw026. 10.1093/aobpla/plw026 PMC4940502 27169609

[pce70775-bib-0017] Dührkop, K. , M. Fleischauer , M. Ludwig , et al. 2019. “SIRIUS 4: A Rapid Tool for Turning Tandem Mass Spectra Into Metabolite Structure Information.” Nature Methods 16: 299–302. 10.1038/s41592-019-0344-8 30886413

[pce70775-bib-0018] Dührkop, K. , L.‐F. Nothias , M. Fleischauer , et al. 2021. “Systematic Classification of Unknown Metabolites Using High‐Resolution Fragmentation Mass Spectra.” Nature Biotechnology 39: 462–471. 10.1038/s41587-020-0740-8 33230292

[pce70775-bib-0019] Dührkop, K. , H. Shen , M. Meusel , J. Rousu , and S. Böcker,. 2015. “Searching Molecular Structure Databases With Tandem Mass Spectra Using CSI:FingerID.” Proceedings of the National Academy of Sciences 112: 12580–12585. 10.1073/pnas.1509788112 PMC4611636 26392543

[pce70775-bib-0020] Endara, M.‐J. , D. L. Forrister , and P. D. Coley . 2023. “The Evolutionary Ecology of Plant Chemical Defenses: From Molecules to Communities.” Annual Review of Ecology, Evolution, and Systematics 54: 107–127.

[pce70775-bib-0021] Erb, M. , and D. J. Kliebenstein . 2020. “Plant Secondary Metabolites as Defenses, Regulators, and Primary Metabolites: The Blurred Functional Trichotomy.” Plant Physiology 184: 39–52. 10.1104/pp.20.00433 PMC7479915 32636341

[pce70775-bib-0022] Ertl, P. , B. Rohde , and P. Selzer . 2000. “Fast Calculation of Molecular Polar Surface Area as a Sum of Fragment‐Based Contributions and Its Application to the Prediction of Drug Transport Properties.” Journal of Medicinal Chemistry 43: 3714–3717. 10.1021/jm000942e 11020286

[pce70775-bib-0023] Ghose, A. K. , and G. M. Crippen . 1986. “Atomic Physicochemical Parameters for Three‐Dimensional Structure‐Directed Quantitative Structure‐Activity Relationships I. Partition Coefficients as a Measure of Hydrophobicity.” Journal of Computational Chemistry 7: 565–577.

[pce70775-bib-0024] Gramlich, S. , P. Sagmeister , S. Dullinger , F. Hadacek , and E. Hörandl . 2016. “Evolution In Situ: Hybrid Origin and Establishment of willows (*Salix L.*) on Alpine Glacier Forefields.” Heredity 116: 531–541. 10.1038/hdy.2016.14 PMC4868266 26980342

[pce70775-bib-0025] Guha, R. 2007. “Chemical Informatics Functionality in R.” Journal of Statistical Software 18: 1–16.

[pce70775-bib-0026] Haghpanah, M. , S. Hashemipetroudi , A. Arzani , and F. Araniti . 2024. “Drought Tolerance in Plants: Physiological and Molecular Responses.” Plants 13: 2962. 10.3390/plants13212962 PMC11548289 39519881

[pce70775-bib-0027] Henderson, D. , J. S. Tello , L. Cayola , et al. 2025. “Testing the Role of Biotic Interactions in Shaping Elevational Diversity Gradients: An Ecological Metabolomics Approach.” Ecology 106: e70069. 10.1002/ecy.70069 40207495

[pce70775-bib-0028] Hjältén, J. , L. Niemi , A. Wennström , L. Ericson , H. Roininen , and R. Julkunen‐Tiitto . 2007. “Variable Responses of Natural Enemies to *Salix triandra* Phenotypes With Different Secondary Chemistry.” Oikos 116: 751–758.

[pce70775-bib-0029] Hörandl, E. , F. Florineth , and F. Hadacek, 2012. *Weiden in Österreich und angrenzenden Gebieten*. Eigenverlag des Institutes für Ingineurbiologie und Landschaftsbau, Department für Bautechnik und Naturgefahren, Univesität für Bodenkultur Wien, Wien.

[pce70775-bib-0030] Karger, D. N. , O. Conrad , J. Böhner , et al. 2017. “Climatologies at High Resolution for the Earth's Land Surface Areas.” Scientific Data 4: 170122. 10.1038/sdata.2017.122 PMC5584396 28872642

[pce70775-bib-0031] Kergunteuil, A. , P. Descombes , G. Glauser , L. Pellissier , and S. Rasmann . 2018. “Plant Physical and Chemical Defence Variation Along Elevation Gradients: A Functional Trait‐Based Approach.” Oecologia 187: 561–571. 10.1007/s00442-018-4162-y 29774426

[pce70775-bib-0032] Kessler, A. , and A. Kalske . 2018. “Plant Secondary Metabolite Diversity and Species Interactions.” Annual Review of Ecology, Evolution, and Systematics 49: 115–138.

[pce70775-bib-0033] Kosiński, P. , K. Sękiewicz , Ł. Walas , A. Boratyński , and M. Dering . 2019. “Spatial Genetic Structure of the Endemic Alpine Plant Salix Serpillifolia: Genetic Swamping on Nunataks Due to Secondary Colonization?” Alpine Botany 129, no. 2: 107–121.

[pce70775-bib-0034] Kumari, M. , and R. Kumar . 2024. “Functional Trait Correlation Network and Proteomic Analysis Reveal Multifactorial Adaptation Mechanisms to a Climatic Gradient Associated With High Altitude in the Himalayan Region.” Plant, Cell & Environment 47: 1556–1574. 10.1111/pce.14830 38268334

[pce70775-bib-0035] Lovering, F. , J. Bikker , and C. Humblet . 2009. “Escape From Flatland: Increasing Saturation as An Approach to Improving Clinical Success.” Journal of Medicinal Chemistry 52: 6752–6756. 10.1021/jm901241e 19827778

[pce70775-bib-0036] Moreira, X. , W. K. Petry , K. A. Mooney , S. Rasmann , and L. Abdala‐Roberts . 2018. “Elevational Gradients in Plant Defences and Insect Herbivory: Recent Advances in the Field and Prospects for Future Research.” Ecography 41: 1485–1496.

[pce70775-bib-0037] Mullin, M. , J. G. Klutsch , J. A. Cale , et al. 2021. “Primary and Secondary Metabolite Profiles of Lodgepole Pine Trees Change With Elevation, but Not With Latitude.” Journal of Chemical Ecology 47: 280–293. 10.1007/s10886-021-01249-y 33651224

[pce70775-bib-0038] Nomoto, H. , R. Berrached , G. Glauser , M. Bueche , and S. Rasmann . 2025a. “Aridity Drives Community‐Wide Shifts Towards Phytochemical Underdispersion.” New Phytologist 248: 1058–1073. 10.1111/nph.70459 40851290

[pce70775-bib-0039] Nomoto, H. , P. Fernández‐Conradi , N. Kjelsberg , et al. 2025b. “Elevation Drives Intraspecific Metabolomic Differentiation in Natural and Experimental Populations.” Plant Biology 27: 773–784. 10.1111/plb.70025 40341698

[pce70775-bib-0080] Novák, P. , P. Neumann and J. Macas 2020. “Global Analysis of Repetitive DNA From Unassembled Sequence Reads Using RepeatExplorer2.” Nature Protocols 15: 3745–3776. 10.1038/s41596-020-0400-y 33097925

[pce70775-bib-0040] Obata, T. , and A. R. Fernie . 2012. “The Use of Metabolomics to Dissect Plant Responses to Abiotic Stresses.” Cellular and Molecular Life Sciences 69: 3225–3243. 10.1007/s00018-012-1091-5 PMC3437017 22885821

[pce70775-bib-0041] Oksanen, J. , F. Blanchet , M. Friendly , et al. 2019. vegan: Community Ecology Package. R package version 2.5‐6. https://CRAN.R-project.org/package=vegan.

[pce70775-bib-0042] Pandey, P. K. , J. Yu , N. Omranian , et al. 2019. “Plasticity in Metabolism Underpins Local Responses to Nitrogen in *Arabidopsisthaliana* Populations.” Plant Direct 3: e00186. 10.1002/pld3.186 PMC6884650 31799492

[pce70775-bib-0043] Pearse, I. S. , R. Paul , and P. J. Ode . 2018. “Variation in Plant Defense Suppresses Herbivore Performance.” Current Biology 28: 1981–1986.e2. 10.1016/j.cub.2018.04.070 29887306

[pce70775-bib-0044] Van de Peer, Y. , T.‐L. Ashman , P. S. Soltis , and D. E. Soltis . 2021. “Polyploidy: An Evolutionary and Ecological Force in Stressful Times.” Plant Cell 33: 11–26. 10.1093/plcell/koaa015 PMC8136868 33751096

[pce70775-bib-0045] Pellissier, L. , X. Moreira , H. Danner , et al. 2016. “The Simultaneous Inducibility of Phytochemicals Related to Plant Direct and Indirect Defences Against Herbivores Is Stronger at Low Elevation.” Journal of Ecology 104: 1116–1125.

[pce70775-bib-0046] Pellissier, L. , A. Roger , J. Bilat , and S. Rasmann . 2014. “High Elevation *Plantago lanceolata* Plants Are Less Resistant to Herbivory Than Their Low Elevation Conspecifics: Is It Just Temperature?” Ecography 37: 950–959.

[pce70775-bib-0047] Petrén, H. , T. G. Köllner , and R. R. Junker . 2023. “Quantifying Chemodiversity Considering Biochemical and Structural Properties of Compounds With the R Package Chemodiv.” New Phytologist 237: 2478–2492. 10.1111/nph.18685 36527232

[pce70775-bib-0048] Pittet, L. , P. Kosiński , N. D. Wagner , and E. Hörandl . 2025a. “Niche Expansion of Polyploid Cytotypes Shaped the Phylogeographical History of the *Salix retusa* Complex in the European Alpine System.” Annals of Botany 136: 903–917. 10.1093/aob/mcaf163 PMC12464947 40708498

[pce70775-bib-0049] Pittet, L. , P. Marinček , P. Kosiński , N. D. Wagner , and E. Hörandl . 2025b. “Hybrid Zones in the European Alps Impact the Phylogeography of Alpine Vicariant Willow Species (*Salix L.*).” Frontiers in Plant Science 16: 1507275. 10.3389/fpls.2025.1507275 PMC11966204 40182553

[pce70775-bib-0050] Ren, S. , J. Cui , L. Wang , Z. Lu , and B. Jin . 2026. “Plant Flavonoids: Biosynthesis, Regulation, and Roles in Biotic and Abiotic Stresses.” Plant, Cell & Environment 49 8: 5664–5684. 10.1111/pce.70540 PMC13353710 42046293

[pce70775-bib-0051] Renoult, S. A. , J. V. Leong , P. Mezzomo , et al. 2025. “Effects of Hybridization on Chemical Diversity and Plant–Insect Herbivore Interactions in *Salix alba *×* fragilis* .” Journal of Experimental Botany 76: eraf296. 10.1093/jxb/eraf296 40619829

[pce70775-bib-0052] Sam, K. , B. Koane , L. Sam , et al. 2020. “Insect Herbivory and Herbivores of *Ficus* Species Along a Rainforest Elevational Gradient in Papua New Guinea.” Biotropica 52: 263–276.

[pce70775-bib-0053] Schmid, R. , S. Heuckeroth , A. Korf , et al. 2023. “Integrative Analysis of Multimodal Mass Spectrometry Data in MZmine 3.” Nature Biotechnology 41: 447–449. 10.1038/s41587-023-01690-2 PMC10496610 36859716

[pce70775-bib-0054] Sedio, B. E. 2017. “Recent Breakthroughs in Metabolomics Promise to Reveal the Cryptic Chemical Traits That Mediate Plant Community Composition, Character Evolution and Lineage Diversification.” New Phytologist 214: 952–958. 10.1111/nph.14438 28134431

[pce70775-bib-0055] Sedio, B. E. , M. J. Spasojevic , J. A. Myers , et al. 2021. “Chemical Similarity of Co‐Occurring Trees Decreases With Precipitation and Temperature in North American Forests.” Frontiers in Ecology and Evolution 9: 679638.

[pce70775-bib-0056] Seshan, V. , and K. Whiting . 2023. “clinfun: Clinical Trial Design and Data Analysis Functions.” R package version 1.1.5. https://CRAN.R-project.org/package=clinfun.

[pce70775-bib-0057] Sun, Z. , K. Zhang , J. Peng , et al. 2026. “Strategies for Balancing Growth and Defence Against Biotic Stress in Legumes.” Plant, Cell & Environment 49: 4388–4402. 10.1111/pce.70070 40665866

[pce70775-bib-0058] Sundqvist, M. K. , N. J. Sanders , and D. A. Wardle . 2013. “Community and Ecosystem Responses to Elevational Gradients: Processes, Mechanisms, and Insights for Global Change.” Annual Review of Ecology, Evolution, and Systematics 44: 261–280.

[pce70775-bib-0059] Taiz L. , E. Zeiger , I. M. Møller and A. Murphy . 2014. Plant Physiology and Development. 6th ed. Sinauer Associates.

[pce70775-bib-0060] Tegelberg, R. , and R. Julkunen‐Tiitto . 2001. “Quantitative Changes in Secondary Metabolites of Dark‐Leaved Willow (*Salix myrsinifolia*) Exposed to Enhanced Ultraviolet‐B Radiation.” Physiologia Plantarum 113: 541–547.

[pce70775-bib-0061] Tossi, V. E. , L. J. Martínez Tosar , L. E. Laino , et al. 2022. “Impact of Polyploidy on Plant Tolerance to Abiotic and Biotic Stresses.” Frontiers in Plant Science 13: 869423. 10.3389/fpls.2022.869423 PMC9441891 36072313

[pce70775-bib-0062] de Villemereuil, P. , M. Mouterde , O. E. Gaggiotti , and I. Till‐Bottraud . 2018. “Patterns of Phenotypic Plasticity and Local Adaptation in the Wide Elevation Range of the Alpine Plant *Arabis alpina* .” Journal of Ecology 106: 1952–1971.

[pce70775-bib-0063] Volf, M. , J. Hrcek , R. Julkunen‐Tiitto , and V. Novotny . 2015. “To Each Its Own: Differential Response of Specialist and Generalist Herbivores to Plant Defence in Willows.” Journal of Animal Ecology 84: 1123–1132. 10.1111/1365-2656.12349 25649252

[pce70775-bib-0064] Volf, M. , J. E. Laitila , J. Kim , et al. 2020. “Compound Specific Trends of Chemical Defences in *Ficus* Along an Elevational Gradient Reflect a Complex Selective Landscape.” Journal of Chemical Ecology 46: 442–454. 10.1007/s10886-020-01173-7 32314119

[pce70775-bib-0065] Volf, M. , J. V. Leong , P. de Lima Ferreira , et al. 2023. “Contrasting Levels of β‐diversity and Underlying Phylogenetic Trends Indicate Different Paths to Chemical Diversity in Highland and Lowland Willow Species.” Ecology Letters 26: 1559–1571. 10.1111/ele.14273 37345539

[pce70775-bib-0066] Volf, M. , S. A. Renoult , S. Panthee , and N. M. van Dam . 2024. “Quantifying Various Aspects of Chemical Diversity in Hybrid Plants Can Help Understanding Ecological Consequences of Hybridization.” American Journal of Botany 111: e16283. 10.1002/ajb2.16283 38332482

[pce70775-bib-0067] Volf, M. , T. Volfová , E. Hörandl , et al. 2022. “Abiotic Stress Rather Than Biotic Interactions Drives Contrasting Trends in Chemical Richness and Variation in Alpine Willows.” Functional Ecology 36: 2701–2712.

[pce70775-bib-0068] Wagner, N. D. , L. He , and E. Hörandl . 2021. “The Evolutionary History, Diversity, and Ecology of Willows (*Salix* L.) in the European Alps.” Diversity 13: 146.

[pce70775-bib-0069] Wagner, N. D. , P. Marinček , L. Pittet , and E. Hörandl . 2023. “Insights into the Taxonomically Challenging Hexaploid Alpine Shrub Willows of *Salix* Sections Phylicifoliae and Nigricantes (Salicaceae).” Plants 12: 1144. 10.3390/plants12051144 PMC10005704 36904002

[pce70775-bib-0070] Walker, T. W. N. , J. M. Alexander , P. M. Allard , et al. 2022. “Functional Traits 2.0: The Power of the Metabolome for Ecology.” Journal of Ecology 110: 4–20.

[pce70775-bib-0071] Walker, T. W. N. , F. Schrodt , P.‐M. Allard , et al. 2023. “Leaf Metabolic Traits Reveal Hidden Dimensions of Plant Form and Function.” Science Advances 9: eadi4029. 10.1126/sciadv.adi4029 PMC10468135 37647404

[pce70775-bib-0072] Wetzel, W. C. , B. D. Inouye , P. G. Hahn , S. R. Whitehead , and N. Underwood . 2023. “Variability in Plant–Herbivore Interactions.” Annual Review of Ecology, Evolution, and Systematics 54: 451–474.

[pce70775-bib-0073] Whitehead, S. R. , E. Bass , A. Corrigan , A. Kessler , and K. Poveda . 2021. “Interaction Diversity Explains the Maintenance of Phytochemical Diversity.” Ecology Letters 24: 1205–1214. 10.1111/ele.13736 33783114

[pce70775-bib-0074] Willighagen, E. L. , J. W. Mayfield , J. Alvarsson , et al. 2017. “The Chemistry Development Kit (CDK) v2. 0: Atom Typing, Depiction, Molecular Formulas, and Substructure Searching.” Journal of Cheminformatics 9: 1–19. 10.1186/s13321-017-0220-4 PMC5461230 29086040

[pce70775-bib-0075] Wood, S. N. 2011. “Fast Stable Restricted Maximum Likelihood and Marginal Likelihood Estimation of Semiparametric Generalized Linear Models.” Journal of the Royal Statistical Society Series B: Statistical Methodology 73: 3–36.

[pce70775-bib-0076] Wu, X. , H. Chen , R. Ding , et al. 2025. “Integrating Transcriptome, Metabolome and Microbiome to Explore the Molecular Mechanism of Phenotypic Plasticity in *P. rotata* During Low‐Altitude Domestication.” Plant, Cell & Environment 48: 8449–8468. 10.1111/pce.70138 40836512

[pce70775-bib-0077] Xin, W. , L. Zhang , W. Zhang , et al. 2019. “An Integrated Analysis of the Rice Transcriptome and Metabolome Reveals Differential Regulation of Carbon and Nitrogen Metabolism in Response to Nitrogen Availability.” International Journal of Molecular Sciences 20: 2349. 10.3390/ijms20092349 PMC6539487 31083591

[pce70775-bib-0078] Zandalinas, S. I. , D. Balfagón , A. Gómez‐Cadenas , and R. Mittler . 2022. “Plant Responses to Climate Change: Metabolic Changes under Combined Abiotic Stresses.” Journal of Experimental Botany 73: 3339–3354. 10.1093/jxb/erac073 35192700

[pce70775-bib-0079] Zhao, C. , M. Wang , Y. Liu , et al. 2025. “mRNA‐lncRNA‐miRNA Co‐Regulates the Accumulation of Flavonoids in Ginkgo biloba in Response to UV‐B Stress.” Plant, Cell & Environment 48: 7095–7106. 10.1111/pce.70003 40518759

