# Supplementary material for: Climate Shapes Elevational Gradients in Concentration, Composition, and Functional Diversity of Willow Metabolomes
Source: Plant Cell Environ. 2026 Jul 31;49(11):8851–66. doi: 10.1111/pce.70775 (PMC13646653; doi:10.1111/pce.70775)
Supplement: Supplementary file 1 [file PCE-49-8851-s001.docx]

**Climate Shapes Elevational Gradients in Concentration, Composition, and Functional Diversity of Willow Metabolomes**

Appendix 1

**Molecular identification**

We extracted total genomic DNA (gDNA) from dried leaves of each studied individual using the MagAttract HMW DNA kit (Qiagen, USA) following the manufacturer's instructions. The quantity and quality of gDNA were verified using Qubit Fluorometer 3.0 (High Sensitivity Kit; Life Technologies) and on a 2% agarose gel. The extracted genomic DNA was used for library preparation and sequencing performed by Novogene (Cambridge, United Kingdom). A total amount of 1.0 μg DNA per sample was used as input material for preparation of the DNA libraries. The genomic DNA was sheared to 350 bp, and then the fragments were end-repaired, A-tailed, and ligated with the NEBNext adapter for Illumina sequencing. P5 and indexed P7 oligos were added to the resulting DNA fragments by PCR. The libraries were purified using the AMPure XP system (Beckman Coulter, USA) and then checked for size distribution using the Agilent 2100 Bioanalyzer (Agilent Technologies, USA) and quantified by real-time PCR (to meet the criteria of 3 nM). Whole genome shotgun sequencing was performed on a Novaseq 6000 platform producing 2x150bp paired end reads.

Ten individuals of *S. appendiculata*, 10 of *S. caprea*, 40 of *S. mielichhoferi* and 27 of *S. myrsinifolia* reliably identified based on their morphology were used in comparative repeat analysis using the RepeatExplorer2 pipeline following Novák *et al.* (2020). Raw Illumina reads were filtered and trimmed to 100 bp using cutadapt 1.15 (Martin, 2011) with options ‘--nextseq-trim=10 --length=100 --minimum-length=100’ supplied with a custom list of the Illumina TruSeq Universal Adapter and Indexed Adapters 1–27. The resulting filtered reads in fastq format were then pseudorandomly sampled, interleaved and converted to fasta format using seqtk (ver. 1.2; https://github.com/lh3/seqtk). We sampled and compared ca. 0.55x equivalent of genome size of each *S. appendiculata* and *S. caprea* and 0.3x equivalent of genome size of each *S. myrsinifolia* and *S. mielichhoferi*. Respective genome sizes, determined by flow cytometry, were 1C = 419 Mbp for *S. appendiculata*, 1C = 451 Mbp for *S. caprea*, 1C = 1,480 Mbp for *S. mielichhoferi* and 1C = 1,091 Mbp for *S. myrsinifolia* (Doležel and Bartoš, 2005). This fits reported diploid level for S*. appendiculata* and *S. caprea* (2n = 38) and hexaploid level (2n =114) for *S. mielichhoferi* and *S. myrsinifolia* (Wagner *et al.*, 2021). The read IDs of the resulting samples were tagged, concatenated, and analysed together using the Singularity container with RepeatExplorer2 with TAREAN (repex_tarean:0.3.12-7a7dc9e) with options ‘--automatic_filtering --mincl 0.001 --assembly_min 3 --taxon VIRIDIPLANTAE4.0’. We then used linear models with empirical Bayes moderation and Benjamini-Hochberg correction fitted in the ‛limma՚ package (Ritchie *et al.*, 2015) on R to identify the read clusters best separating the reliably identified *S. appendiculata* from *S. caprea* individuals and *S. myrsinifolia* individuals from *S. mielichhoferi* individuals. For the identification we used the 50 clusters best separating *S. appendiculata* from *S. caprea*, and 100 clusters best separating *S.* *mielichhoferi* from *S. myrsinifolia* that has a larger genome size than the first species pair.

Consensus sequences of these identified diagnostic repeat clusters were concatenated, TAREAN consensus dimer sequences were used where available for identified tandem repeats. The resulting reference was indexed and used for mapping of filtered (see above) Illumina sequencing data of all sampled individuals using bowtie2 (Langmead and Salzberg, 2012) with options '-N 1 -X 101'. We used samtools view 1.9. (Danecek *et al.*, 2021) with flag ‘-F 256’ to filter only primary alignments. The resulting bam files were sorted using samtools sort and used to calculate per base read depth using bedtools genomecov with options '-d -ibam' (Quinlan and Hall, 2010). The read depth was summed across the repeat clusters using bedtools groupby with option ‘-o sum’. Finally, we normalized the number of reads per diagnostic repeat clusters of each individual based on their respective total amount of filtered Illumina sequencing data used for mapping. We performed Principal Component Analysis (PCA) in Canoco 5 (ter Braak and Smilauer, 2012) to visualize how the individual samples clustered. We performed separate analyses for *S. appendiculata* and *S. caprea* and for *S. mielichhoferi* and *S. myrsinifolia*.

Appendix Fig. 1. PCA plot showing the clustering of studied willow individuals based on the standardized read numbers of diagnostic repeat clusters for *S. appendiculata* and *S. caprea*. Individuals are represented as circles or squares. Those reliably identified based on morphology and used to define diagnostic repeat clusters are shown in dark blue (*S. appendiculata*) and dark red (*S. caprea*). Other individuals with clear morphological traits of one of the two species are shown in light blue (*S. appendiculata*) and light red (*S. caprea*). Four individuals exhibiting intermediate morphology are shown in yellow. Two of these clustered within the *S. appendiculata* group and we treated them as such in our subsequent analyses. Individuals in the blue cluster were considered to be *S. appendiculata* and individuals in the red cluster *S. caprea*. The remaining three individuals, located outside these clusters, were tentatively identified as potential hybrids and excluded from further analyses.

In the case of *S. appendiculata* and *S. caprea*, the first two PCA axes explained 42.09% and 7.92% of the variation in read number for the diagnostic repeat clusters (Appendix Fig. 1). Individuals reliably identified based on morphology (that we used to select the diagnostic repeat clusters) clustered with others exhibiting typical morphology of the respective species. The only exception was individual ALP047, which appeared as an outlier within the *S. appendiculata* group. However, ALP047 displayed clear *S. appendiculata* morphology with no traits characteristic of *S. caprea* or related species, and it was distant from the *S. caprea* cluster. Therefore, we retained it as *S. appendiculata*. Four individuals exhibited intermediate morphology between *S. appendiculata* and *S. caprea*. Two of them grouped within the *S. appendiculata* cluster and were considered *S. appendiculata* for subsequent analyses. The remaining two individuals (ALP018 and ALP031), positioned between the *S. appendiculata* and *S. caprea* clusters, were tentatively identified as potential hybrids. This was also the case for ALP108, based on its position, despite its morphological identification as *S. appendiculata*. However, given their small number, it was not meaningful to treat potential hybrids as a separate category. Consequently, we excluded these three individuals from further analyses.

Appendix Fig. 2. PCA plot showing the clustering of studied willow individuals based on the standardized read numbers of diagnostic repeat clusters for *S. mielichhoferi* and *S. myrsinifolia*. Individuals are represented as circles. Those reliably identified based on morphology and used to define diagnostic repeat clusters are shown in purple (*S. mielichhoferi*) and green (*S. myrsinifolia*). Individuals with a clearly intermediate morphology are in yellow and individuals that could not be reliably assigned to any category based on their morphology are in grey. Individuals in the green cluster were considered to be *S. myrsinifolia* and individuals in the purple cluster were considered *S. mielichhoferi* in our following analyses.

In the case of *S. mielichhoferi* and *S. myrsinifolia*, the first two PCA axes explained 24.83% and 6.17% of the variation in read counts for the diagnostic repeat clusters (Appendix Fig. 2). Individuals with intermediate morphology, as well as some that could not be reliably assigned to a species based on morphology, were distributed across both clusters. These individuals were classified as either *S. mielichhoferi* or *S. myrsinifolia* in subsequent analyses according to their PCA placement.

**References**

**Danecek P, Bonfield JK, Liddle J, Marshall J, Ohan V, Pollard MO, Whitwham A, Keane T, McCarthy SA, Davies RM**. 2021. Twelve years of SAMtools and BCFtools. Gigascience **10**, giab008.

**Doležel J, Bartoš J**. 2005. Plant DNA flow cytometry and estimation of nuclear genome size. Annals Of Botany **95**, 99-110.

**Langmead B, Salzberg SL**. 2012. Fast gapped-read alignment with Bowtie 2. Nature Methods **9**, 357-359.

**Martin M**. 2011. Cutadapt removes adapter sequences from high-throughput sequencing reads. EMBnet. journal **17**, pp. 10-12.

**Novák P, Neumann P, Macas J**. 2020. Global analysis of repetitive DNA from unassembled sequence reads using RepeatExplorer2. Nature Protocols **15**, 3745-3776.

**Quinlan AR, Hall IM**. 2010. BEDTools: a flexible suite of utilities for comparing genomic features. Bioinformatics **26**, 841-842.

**Ritchie ME, Phipson B, Wu D, Hu Y, Law CW, Shi W, Smyth GK**. 2015. limma powers differential expression analyses for RNA-sequencing and microarray studies. Nucleic Acids Research **43**, e47-e47.

**ter Braak CJ, Smilauer P**. 2012. *Canoco Reference Manual and User’s Guide: Software for Ordination (version 5.0)*. Ithaca: Microcomputer power.

**Wagner ND, He L, Hörandl E**. 2021. The evolutionary history, diversity, and ecology of willows (*Salix* L.) in the European Alps. Diversity **13**, 146.
