# Supplementary material for: Climate Shapes Elevational Gradients in Concentration, Composition, and Functional Diversity of Willow Metabolomes
Source: Plant Cell Environ. 2026 Jul 31;49(11):8851–66. doi: 10.1111/pce.70775 (PMC13646653; doi:10.1111/pce.70775)
Supplement: Supplementary file 2 [file PCE-49-8851-s002.docx]

**Climate Shapes Elevational Gradients in Concentration, Composition, and Functional Diversity of Willow Metabolomes**

Supplementary 1

**Figure S1.** Variation between the studied plants as visualized with Principal Component Analysis (PCAs) based on the climatic conditions (A) and the soil parameters (B). The first unconstrained axis explained 81.4% of variation in the analysis based on climatic conditions and 48.0% of variation in the analysis based on soil parameters. Plant individuals appear as circles and climatic and soil parameters appear as arrows.

**Figure S2.** Correlation between elevation and climatic conditions (A), soil conditions (B), herbivory (C), variation in climatic conditions (D), variation in soil conditions (E) and variation in herbivory (F). The variation corresponds to the log-transformed variance of residuals from linear models including species and region as covariates. Relationships were fitted using Linear Models. Significant trends are shown with solid lines, while non-significant relationships are represented by dashed lines.

**Table S1.** Sites, their GPS coordinates, elevation, and the number of *Salix appendiculata*, *S. caprea*, *S. mielichhoferi* and *S. myrsinifolia* found. Numbers reported here refer to the number of individuals resulting from revised identifications based on molecular data.

| **Site** | **GPS coordinates** | **Elevation (m asl)** | ***S. appendiculata*** | ***S. caprea*** | **S. mielichhoferi** | **S. myrsinifolia** | **Total** |
| --- | --- | --- | --- | --- | --- | --- | --- |
| 5 | N47° 02.356' E12° 50.113' | 1159 | 5 | 4 | 1 | 3 | 13 |
| 6 | N47° 03.682' E12° 45.826' | 1958 | 0 | 0 | 4 | 0 | 4 |
| 8 | N47° 02.656' E12° 53.853' | 1652 | 0 | 0 | 4 | 1 | 5 |
| 10 | N47° 03.568' E12° 49.921' | 1765 | 5 | 0 | 2 | 0 | 7 |
| 20 | N47° 02.676' E12° 48.522' | 1377 | 2 | 0 | 2 | 0 | 4 |
| 21 | N47° 02.109' E12° 47.708' | 1609 | 4 | 0 | 8 | 0 | 12 |
| 22 | N47° 04.116' E12° 49.163' | 1822 | 0 | 0 | 5 | 0 | 5 |
| 23 | N47° 03.794' E12° 49.109' | 1778 | 5 | 2 | 2 | 0 | 9 |
| 24 | N47° 01.669' E12° 51.321' | 1039 | 10 | 0 | 0 | 5 | 15 |
| 26 | N46° 59.375' E12° 52.495' | 977 | 3 | 5 | 0 | 5 | 13 |
| 27 | N46° 58.275' E12° 53.535' | 971 | 3 | 5 | 0 | 4 | 12 |
| 29 | N47° 02.723' E12° 48.670' | 1250 | 0 | 0 | 6 | 0 | 6 |
| 35 | N47° 04.796' E12° 45.970' | 2199 | 0 | 0 | 5 | 0 | 5 |
| 36 | N47° 04.239' E12° 45.950' | 2003 | 3 | 0 | 5 | 0 | 8 |
| 37 | N47° 03.255' E12° 50.012' | 1798 | 0 | 0 | 1 | 0 | 1 |
| 38 | N47° 04.253' E12° 46.094' | 2103 | 5 | 0 | 5 | 0 | 10 |
| T2 | N47° 00.401' E12° 40.099' | 1398 | 0 | 0 | 3 | 2 | 5 |
| T4 | N47° 01.072' E12° 41.188' | 1817 | 0 | 0 | 4 | 1 | 5 |
| T7 | N47° 02.158' E12° 41.393' | 1963 | 0 | 0 | 9 | 0 | 9 |
| T8 | N46° 57.904' E12° 37.492' | 1065 | 0 | 0 | 0 | 4 | 4 |
| T9 | N46° 59.009' E12° 37.948' | 1178 | 0 | 0 | 0 | 5 | 5 |

**Table S2.** Studied plant individuals and the chemical traits measured with untargeted metabolomics. The measured chemical traits include concentration of metabolites (measured as the area under the peaks/mg), richness of metabolites (number of metabolites in a sample) and structural diversity (measured as functional Hill diversity of metabolites).

| **Tree individuals** | **Concentration all metabolites** | **Concentration flavonoids** | **Concentration salicinoids** | **Concentration primary metabolites** | **Richness all metabolites** | **Richness flavonoids** | **Richness salicinoids** | **Richness primary metabolites** | **Struct. α-div all metabolites** | **Struct. α-div flavonoids** | **Struct. α-div salicinoids** | **Struct. α-div primary metabolites** |
| --- | --- | --- | --- | --- | --- | --- | --- | --- | --- | --- | --- | --- |
| ALP001 | 101935621.9 | 21119389.0 | 104430.8 | 12602045.6 | 229 | 27 | 3 | 36 | 1245.60 | 22.87 | 0.60 | 92.83 |
| ALP002 | 62789988.9 | 13431988.3 | 60636.8 | 8738942.5 | 191 | 25 | 3 | 30 | 708.31 | 16.93 | 0.64 | 53.59 |
| ALP003 | 107804094.5 | 25908981.7 | 173158.1 | 12974849.8 | 249 | 33 | 5 | 34 | 2316.65 | 29.51 | 1.46 | 87.74 |
| ALP004 | 359689542.3 | 8665635.8 | 26081590.8 | 38782660.0 | 487 | 28 | 32 | 44 | 9413.26 | 25.87 | 4.34 | 200.14 |
| ALP005 | 77703475.0 | 18295220.0 | 87351.4 | 8762706.7 | 236 | 28 | 2 | 34 | 3006.15 | 29.40 | 0.18 | 138.83 |
| ALP006 | 88455154.7 | 18024145.9 | 78387.7 | 11264416.2 | 222 | 30 | 2 | 33 | 1280.57 | 30.20 | 0.18 | 84.14 |
| ALP010 | 351146384.7 | 8372589.1 | 38409302.3 | 21447586.7 | 485 | 30 | 33 | 39 | 7825.28 | 27.56 | 4.20 | 142.86 |
| ALP011 | 425357116.0 | 13849129.4 | 74293016.2 | 25208010.4 | 484 | 30 | 30 | 40 | 5650.95 | 30.54 | 2.23 | 86.61 |
| ALP012 | 460582016.4 | 10805889.2 | 94724375.1 | 27548421.0 | 470 | 29 | 27 | 37 | 4314.44 | 27.18 | 2.07 | 42.44 |
| ALP014 | 87903012.5 | 19441441.6 | 96295.2 | 11499707.8 | 250 | 25 | 3 | 36 | 1384.39 | 16.99 | 0.64 | 72.78 |
| ALP015 | 124408852.1 | 26229045.6 | 141846.0 | 16004683.8 | 241 | 23 | 4 | 31 | 1400.35 | 17.50 | 1.18 | 77.21 |
| ALP016 | 102984684.3 | 28525649.5 | 580607.0 | 13135175.5 | 269 | 25 | 8 | 38 | 2113.50 | 18.24 | 3.66 | 95.59 |
| ALP017 | 113700727.8 | 27672716.2 | 145021.3 | 14591602.9 | 241 | 32 | 4 | 31 | 1698.67 | 33.07 | 1.08 | 54.98 |
| ALP019 | 106348856.6 | 26121621.7 | 377463.0 | 13104356.6 | 250 | 28 | 5 | 34 | 2724.24 | 24.96 | 1.92 | 88.65 |
| ALP020 | 122993444.7 | 20024227.8 | 54026.3 | 15392473.7 | 226 | 28 | 1 | 33 | 1994.92 | 24.57 | 0.00 | 150.96 |
| ALP021 | 467026809.2 | 6881083.2 | 57674023.9 | 27270841.5 | 502 | 27 | 32 | 39 | 6383.86 | 26.26 | 3.64 | 102.00 |
| ALP022 | 306958967.4 | 9456458.3 | 31062736.9 | 20333190.4 | 456 | 25 | 28 | 36 | 7114.48 | 20.67 | 4.73 | 76.56 |
| ALP023 | 79282018.7 | 10962948.6 | 48794.9 | 13281083.5 | 214 | 21 | 1 | 32 | 2023.89 | 11.43 | 0.00 | 102.70 |
| ALP024 | 405985336.2 | 10712142.4 | 110804252.2 | 24318741.6 | 450 | 29 | 27 | 36 | 3444.40 | 35.69 | 1.61 | 48.49 |
| ALP025 | 364012219.9 | 8818504.3 | 71129093.8 | 27303989.0 | 474 | 26 | 30 | 32 | 3616.50 | 21.72 | 2.35 | 36.01 |
| ALP026 | 122648976.4 | 16927778.7 | 5047960.6 | 16193890.7 | 273 | 23 | 11 | 33 | 1814.55 | 16.97 | 1.27 | 107.11 |
| ALP027 | 100777978.4 | 21109995.2 | 383980.7 | 13727767.6 | 247 | 24 | 7 | 33 | 1715.90 | 16.13 | 2.09 | 83.32 |
| ALP028 | 421682655.6 | 12998230.6 | 98136479.3 | 28571875.9 | 449 | 25 | 26 | 32 | 3731.66 | 26.41 | 2.11 | 28.12 |
| ALP029 | 123979055.8 | 38070533.7 | 85438.0 | 14517227.9 | 229 | 28 | 3 | 30 | 1082.69 | 17.13 | 0.48 | 67.81 |
| ALP030 | 142821909.5 | 43161609.8 | 124690.6 | 16428497.6 | 239 | 30 | 4 | 30 | 1633.83 | 25.87 | 0.95 | 60.66 |
| ALP032 | 103905633.5 | 17157514.3 | 332680.9 | 12040933.4 | 262 | 23 | 7 | 35 | 2210.60 | 15.39 | 2.29 | 103.47 |
| ALP033 | 156087288.2 | 54282913.9 | 260021.2 | 15306143.2 | 257 | 27 | 4 | 36 | 1189.61 | 21.48 | 1.28 | 100.55 |
| ALP034 | 466406379.8 | 8995191.3 | 70205280.8 | 28073051.3 | 487 | 28 | 25 | 37 | 6163.65 | 26.56 | 2.73 | 92.59 |
| ALP035 | 125549786.7 | 24967293.3 | 78290.9 | 13599338.7 | 242 | 28 | 4 | 31 | 848.34 | 22.61 | 1.22 | 94.99 |
| ALP036 | 123852582.1 | 23050317.8 | 57536.8 | 15565679.9 | 229 | 21 | 2 | 35 | 834.14 | 9.71 | 0.18 | 71.62 |
| ALP037 | 589421734.0 | 4705567.8 | 194085352.7 | 36350457.9 | 473 | 25 | 29 | 39 | 2813.33 | 33.54 | 1.96 | 41.26 |
| ALP038 | 109278478.4 | 19280976.4 | 48083.6 | 15079885.5 | 237 | 22 | 4 | 35 | 1301.57 | 10.80 | 1.19 | 85.38 |
| ALP039 | 358020677.5 | 18552121.7 | 75213252.5 | 26981345.9 | 483 | 28 | 29 | 41 | 3472.17 | 19.05 | 1.84 | 66.59 |
| ALP040 | 105297620.6 | 18155568.9 | 3195597.0 | 12168210.5 | 268 | 22 | 12 | 37 | 2510.16 | 10.93 | 2.03 | 109.29 |
| ALP041 | 118172263.4 | 19871711.3 | 52576.9 | 18622566.8 | 249 | 26 | 3 | 35 | 1607.43 | 16.14 | 0.63 | 105.23 |
| ALP042 | 139433010.8 | 32955603.4 | 278172.1 | 15443368.9 | 270 | 29 | 6 | 38 | 1360.42 | 30.90 | 1.87 | 99.24 |
| ALP043 | 115291534.9 | 29054380.4 | 40022.1 | 11914019.3 | 241 | 27 | 2 | 35 | 1182.80 | 22.64 | 0.18 | 90.95 |
| ALP044 | 127349353.4 | 25828734.5 | 1074509.4 | 13384917.7 | 263 | 22 | 9 | 36 | 1108.97 | 9.29 | 1.87 | 79.52 |
| ALP045 | 460885374.9 | 5513041.1 | 107406750.5 | 36024823.9 | 454 | 25 | 27 | 39 | 3209.57 | 34.77 | 1.64 | 36.19 |
| ALP046 | 343091204.9 | 14582665.6 | 84029314.2 | 27546698.1 | 453 | 28 | 28 | 35 | 4045.41 | 23.27 | 2.16 | 47.27 |
| ALP047 | 551814057.3 | 7358236.2 | 181157613.9 | 35675819.7 | 476 | 21 | 30 | 35 | 2802.44 | 23.20 | 2.23 | 31.92 |
| ALP048 | 375034292.1 | 15740120.9 | 67293718.7 | 22748007.5 | 475 | 30 | 34 | 36 | 5950.58 | 31.54 | 2.45 | 69.31 |
| ALP050 | 74314002.9 | 11129440.8 | 23023.6 | 11253872.8 | 178 | 14 | 1 | 27 | 751.83 | 3.43 | 0.00 | 58.03 |
| ALP051 | 79182233.4 | 10869212.0 | 26081.6 | 13777976.6 | 159 | 13 | 1 | 26 | 487.20 | 5.06 | 0.00 | 32.33 |
| ALP052 | 378247283.9 | 2940169.2 | 85083133.6 | 22560576.4 | 332 | 10 | 24 | 25 | 2309.50 | 5.12 | 2.84 | 30.39 |
| ALP053 | 526611375.7 | 5821922.5 | 131292744.8 | 28488790.3 | 422 | 16 | 29 | 30 | 2952.26 | 12.46 | 2.64 | 28.19 |
| ALP054 | 124146494.5 | 20935373.4 | 1072652.3 | 13784203.5 | 265 | 28 | 9 | 35 | 1655.53 | 21.54 | 1.92 | 118.01 |
| ALP055 | 80856366.2 | 17079604.7 | 64991.8 | 10705851.1 | 210 | 23 | 5 | 31 | 1010.97 | 14.44 | 1.65 | 80.24 |
| ALP056 | 485707505.6 | 17490897.7 | 84817206.1 | 32879023.9 | 473 | 27 | 28 | 39 | 4723.64 | 19.33 | 2.42 | 35.67 |
| ALP057 | 106656329.0 | 24654726.0 | 97390.0 | 13707637.6 | 228 | 31 | 4 | 30 | 1379.30 | 24.92 | 0.98 | 58.88 |
| ALP058 | 107868875.6 | 22007158.0 | 219579.5 | 14353615.3 | 255 | 32 | 5 | 39 | 1561.25 | 40.66 | 1.50 | 93.75 |
| ALP059 | 134242452.4 | 22797728.3 | 2089539.8 | 14496342.6 | 277 | 31 | 15 | 34 | 2289.66 | 37.54 | 3.63 | 91.71 |
| ALP060 | 96056896.6 | 16550341.9 | 123925.9 | 10561333.3 | 248 | 27 | 5 | 34 | 1447.45 | 26.75 | 1.15 | 113.23 |
| ALP061 | 551895310.0 | 13053180.3 | 153375283.9 | 33831955.4 | 476 | 28 | 33 | 34 | 4067.00 | 27.79 | 5.24 | 24.19 |
| ALP062 | 644649806.1 | 15956682.9 | 197822554.5 | 40383927.5 | 485 | 24 | 38 | 39 | 4649.67 | 17.50 | 5.47 | 34.42 |
| ALP063 | 575094203.4 | 11037239.2 | 218870107.6 | 36364794.4 | 456 | 23 | 29 | 39 | 2187.41 | 16.53 | 2.79 | 26.60 |
| ALP064 | 540256338.8 | 44393343.1 | 116002342.9 | 42788453.5 | 489 | 29 | 33 | 34 | 3620.98 | 11.23 | 2.91 | 32.91 |
| ALP065 | 582122352.3 | 51544896.2 | 149236664.6 | 45979466.0 | 484 | 33 | 31 | 37 | 3332.19 | 12.44 | 3.50 | 22.52 |
| ALP066 | 676649809.4 | 18011301.0 | 112750134.9 | 37397729.6 | 524 | 28 | 34 | 38 | 4353.40 | 17.96 | 3.13 | 34.89 |
| ALP067 | 111023714.8 | 19267460.2 | 67637.1 | 13690605.7 | 242 | 24 | 4 | 33 | 1833.74 | 14.59 | 1.24 | 70.47 |
| ALP068 | 140199081.2 | 42054532.7 | 127610.0 | 14158813.1 | 255 | 24 | 4 | 31 | 1091.45 | 14.37 | 1.28 | 61.94 |
| ALP069 | 118890677.9 | 27898385.7 | 78983.2 | 16315147.1 | 263 | 28 | 3 | 34 | 2010.95 | 19.87 | 0.79 | 62.92 |
| ALP070 | 157852929.1 | 42582382.0 | 81048.0 | 15929923.1 | 258 | 32 | 4 | 32 | 1224.39 | 20.70 | 1.27 | 58.37 |
| ALP071 | 113898392.7 | 24442889.3 | 536859.1 | 14669781.4 | 280 | 26 | 7 | 35 | 2725.39 | 17.72 | 2.47 | 76.09 |
| ALP072 | 552195770.6 | 24488686.7 | 86312435.7 | 25159507.8 | 531 | 30 | 35 | 35 | 10453.57 | 20.46 | 13.82 | 60.92 |
| ALP073 | 512433396.3 | 9091777.1 | 158139703.1 | 36709504.1 | 496 | 20 | 34 | 36 | 4460.08 | 13.27 | 5.33 | 23.88 |
| ALP074 | 464994350.4 | 12471056.3 | 115868179.4 | 36484502.7 | 510 | 25 | 35 | 39 | 5428.47 | 15.76 | 3.88 | 27.50 |
| ALP075 | 634874470.7 | 8156229.2 | 206625264.6 | 40944791.9 | 477 | 21 | 34 | 37 | 3550.54 | 14.08 | 4.71 | 21.35 |
| ALP076 | 517532805.5 | 6399180.6 | 122570990.7 | 39449601.8 | 484 | 21 | 36 | 36 | 4080.14 | 11.41 | 5.66 | 49.32 |
| ALP077 | 120627601.3 | 25989539.2 | 309890.4 | 15670952.4 | 263 | 24 | 6 | 38 | 2322.08 | 16.48 | 1.56 | 69.04 |
| ALP078 | 136871932.4 | 12971395.0 | 215169.4 | 56028503.6 | 229 | 21 | 3 | 37 | 848.89 | 16.92 | 0.68 | 34.04 |
| ALP079 | 110833910.0 | 18761430.4 | 265453.8 | 15688042.1 | 252 | 26 | 6 | 38 | 1652.86 | 21.31 | 1.71 | 89.84 |
| ALP080 | 123817026.7 | 24036825.0 | 82780.8 | 16122185.0 | 252 | 23 | 5 | 34 | 969.02 | 13.59 | 1.60 | 83.71 |
| ALP081 | 470488680.7 | 13651530.8 | 128717756.4 | 42761915.8 | 485 | 29 | 34 | 38 | 3533.76 | 23.24 | 2.44 | 24.61 |
| ALP082 | 125360769.9 | 28337556.7 | 195834.3 | 18838602.3 | 261 | 28 | 6 | 36 | 2307.48 | 31.16 | 1.89 | 70.58 |
| ALP083 | 138113694.7 | 31051438.1 | 222751.1 | 18045798.3 | 261 | 24 | 5 | 37 | 1668.38 | 18.33 | 1.88 | 77.22 |
| ALP085 | 593099016.2 | 13981232.4 | 186194449.6 | 35326312.2 | 491 | 29 | 31 | 41 | 4338.40 | 28.16 | 2.85 | 69.77 |
| ALP086 | 496330573.7 | 12596737.8 | 74538110.6 | 30464717.5 | 518 | 31 | 38 | 38 | 8577.67 | 25.40 | 4.92 | 115.27 |
| ALP087 | 423559691.8 | 11574002.1 | 105569885.8 | 30636003.8 | 511 | 28 | 34 | 34 | 4559.77 | 24.93 | 2.22 | 46.97 |
| ALP088 | 422832711.1 | 24452635.8 | 97425412.5 | 33120699.0 | 493 | 30 | 34 | 40 | 6383.37 | 30.65 | 3.17 | 48.77 |
| ALP089 | 589001892.3 | 13285679.2 | 140082071.2 | 43870507.9 | 508 | 23 | 34 | 39 | 4136.83 | 16.55 | 2.59 | 42.42 |
| ALP090 | 469281904.5 | 13248398.2 | 127049863.3 | 33615032.7 | 484 | 29 | 29 | 39 | 3489.18 | 27.60 | 2.06 | 43.14 |
| ALP091 | 274243362.4 | 18025340.5 | 61622265.9 | 25325742.9 | 492 | 32 | 29 | 39 | 4682.28 | 33.86 | 2.06 | 42.15 |
| ALP092 | 373849925.1 | 10178660.1 | 59397277.2 | 17739550.6 | 489 | 26 | 37 | 38 | 7165.03 | 18.08 | 15.81 | 87.73 |
| ALP093 | 104532319.0 | 16677522.2 | 212480.8 | 13421636.4 | 234 | 24 | 4 | 34 | 1333.88 | 15.58 | 1.39 | 63.87 |
| ALP094 | 367513171.4 | 13565775.7 | 87914020.5 | 31060783.0 | 485 | 28 | 34 | 39 | 4369.32 | 22.25 | 2.51 | 46.89 |
| ALP095 | 123087201.4 | 14136431.7 | 11272872.8 | 12779760.0 | 413 | 31 | 29 | 36 | 4894.76 | 30.60 | 3.46 | 78.97 |
| ALP096 | 415123230.2 | 13120599.2 | 115469630.4 | 30222382.6 | 458 | 23 | 26 | 37 | 3262.67 | 23.32 | 1.85 | 38.58 |
| ALP097 | 86569524.0 | 19143016.0 | 470633.4 | 11248633.8 | 252 | 26 | 7 | 38 | 1985.74 | 24.42 | 2.01 | 78.69 |
| ALP099 | 331076590.6 | 8482399.0 | 90198248.0 | 28378152.6 | 487 | 25 | 31 | 43 | 3837.65 | 21.31 | 3.24 | 25.77 |
| ALP100 | 376060066.3 | 14781470.2 | 80762697.6 | 29376232.8 | 492 | 27 | 34 | 40 | 5887.85 | 24.62 | 5.26 | 50.96 |
| ALP101 | 92370793.5 | 14755504.6 | 142062.6 | 11132990.8 | 250 | 22 | 3 | 36 | 1359.36 | 16.61 | 0.52 | 83.71 |
| ALP102 | 605771277.7 | 14386308.5 | 206122704.7 | 36749932.8 | 487 | 24 | 34 | 38 | 3160.68 | 20.37 | 4.70 | 22.75 |
| ALP103 | 416023571.8 | 7220398.4 | 107964124.9 | 28666763.6 | 481 | 23 | 32 | 39 | 4248.24 | 20.03 | 5.20 | 53.29 |
| ALP104 | 112879612.4 | 15713099.7 | 1260570.4 | 11878041.6 | 271 | 23 | 11 | 37 | 1950.35 | 13.86 | 3.09 | 102.84 |
| ALP105 | 274880076.4 | 5124376.0 | 78730124.7 | 23305601.7 | 459 | 25 | 32 | 36 | 2748.84 | 18.78 | 1.56 | 27.97 |
| ALP106 | 98894696.3 | 23858954.6 | 33502.9 | 10185633.0 | 234 | 22 | 4 | 31 | 1398.17 | 13.93 | 1.04 | 73.57 |
| ALP107 | 436226244.3 | 10037057.9 | 147592868.7 | 33706966.6 | 480 | 22 | 32 | 36 | 2768.60 | 16.63 | 2.73 | 24.06 |
| ALP110 | 495556765.8 | 15556353.2 | 155870679.8 | 34121678.2 | 465 | 21 | 37 | 35 | 2601.82 | 14.14 | 3.22 | 22.64 |
| ALP111 | 567986157.2 | 15211061.2 | 184941532.6 | 40734401.2 | 497 | 28 | 31 | 37 | 2237.35 | 14.44 | 2.23 | 22.82 |
| ALP112 | 559172377.3 | 20172559.2 | 183065006.2 | 40416654.5 | 482 | 22 | 32 | 37 | 2902.72 | 10.20 | 2.70 | 28.59 |
| ALP113 | 593175244.2 | 21603634.6 | 173583647.9 | 37731035.0 | 498 | 28 | 34 | 39 | 4204.18 | 18.97 | 4.90 | 29.37 |
| ALP114 | 542894413.7 | 23081687.2 | 127552386.0 | 32944141.8 | 499 | 22 | 32 | 38 | 5217.13 | 11.58 | 7.58 | 48.09 |
| ALP115 | 428158376.0 | 10829858.7 | 74437696.2 | 23651366.2 | 501 | 24 | 37 | 38 | 6487.60 | 17.39 | 8.49 | 70.26 |
| ALP116 | 710836277.2 | 9105114.1 | 238434012.6 | 45367736.5 | 492 | 23 | 31 | 35 | 2503.14 | 14.78 | 3.08 | 24.87 |
| ALP117 | 694170863.5 | 17409582.8 | 227516363.6 | 42557865.5 | 478 | 20 | 30 | 37 | 2365.55 | 8.19 | 2.86 | 18.21 |
| ALP118 | 670123676.9 | 16018979.3 | 220080123.5 | 47046965.9 | 466 | 22 | 31 | 39 | 1730.48 | 11.37 | 2.58 | 19.61 |
| ALP119 | 134154447.0 | 27149428.4 | 320133.9 | 11265989.5 | 257 | 25 | 7 | 33 | 1124.94 | 16.67 | 3.65 | 79.05 |
| ALP120 | 101979694.8 | 21317896.6 | 799821.0 | 10275794.5 | 271 | 23 | 9 | 35 | 2190.88 | 18.03 | 2.27 | 81.55 |
| ALP121 | 106111917.1 | 24633124.2 | 16514.3 | 11715642.2 | 231 | 28 | 2 | 32 | 1365.29 | 33.86 | 0.00 | 54.78 |
| ALP122 | 97817888.7 | 16363206.0 | 74352.7 | 11788910.9 | 251 | 25 | 4 | 31 | 1796.76 | 19.37 | 1.59 | 57.06 |
| ALP123 | 493388319.1 | 10029648.8 | 109425782.2 | 28528097.2 | 503 | 24 | 33 | 35 | 5120.01 | 15.26 | 6.31 | 50.91 |
| ALP124 | 729792734.8 | 15996880.3 | 196373271.0 | 51493941.2 | 467 | 20 | 32 | 36 | 2373.70 | 10.17 | 2.88 | 18.48 |
| ALP125 | 671990685.2 | 13875095.0 | 232809330.7 | 46665114.3 | 464 | 21 | 36 | 36 | 2020.08 | 10.07 | 3.15 | 17.12 |
| ALP126 | 702783471.2 | 8062893.6 | 212453535.3 | 54326710.2 | 474 | 20 | 31 | 35 | 2052.89 | 11.33 | 2.53 | 18.57 |
| ALP127 | 579710012.0 | 13666836.7 | 145777363.5 | 39780669.1 | 461 | 19 | 39 | 36 | 3221.64 | 8.15 | 2.12 | 31.55 |
| ALP128 | 803705311.1 | 6913803.5 | 243623190.0 | 48766390.9 | 440 | 16 | 34 | 30 | 2107.15 | 5.50 | 3.24 | 18.04 |
| ALP129 | 423475035.3 | 7063438.8 | 158357503.5 | 21921073.7 | 383 | 13 | 35 | 23 | 1576.94 | 6.74 | 3.54 | 27.67 |
| ALP130 | 76010346.3 | 5442588.7 | 7438312.0 | 4558055.8 | 176 | 8 | 11 | 19 | 710.90 | 2.80 | 0.50 | 49.46 |
| ALP131 | 97558922.7 | 12391857.1 | 219790.0 | 10078582.4 | 202 | 12 | 5 | 25 | 498.51 | 4.60 | 1.41 | 40.76 |
| ALP132 | 440550052.4 | 12337615.2 | 121001554.3 | 19363198.7 | 372 | 13 | 32 | 20 | 2804.01 | 7.92 | 7.66 | 10.40 |
| ALP134 | 677519735.6 | 9838051.4 | 359531923.4 | 22798006.7 | 365 | 17 | 23 | 23 | 888.33 | 10.93 | 3.50 | 16.18 |
| ALP135 | 112543674.1 | 5727876.5 | 986181.2 | 18427591.7 | 200 | 10 | 5 | 24 | 720.38 | 2.23 | 0.99 | 25.14 |
| ALP136 | 659462302.5 | 11387811.3 | 303960653.9 | 21253226.1 | 380 | 14 | 22 | 22 | 1135.55 | 11.08 | 2.54 | 23.73 |
| ALP139 | 117526063.0 | 8864230.8 | 448229.8 | 11913533.5 | 207 | 13 | 5 | 22 | 1192.59 | 3.78 | 1.24 | 22.62 |
| ALP140 | 547429686.0 | 2203234.3 | 244040324.3 | 20082814.9 | 365 | 11 | 30 | 23 | 1303.40 | 7.89 | 2.99 | 27.48 |
| ALP141 | 97012240.8 | 15519231.6 | 12520.0 | 12388120.8 | 198 | 19 | 1 | 25 | 1310.31 | 8.37 | 0.00 | 45.94 |
| ALP142 | 100456708.3 | 15665592.7 | 4916.7 | 8639452.9 | 190 | 13 | 1 | 19 | 997.74 | 9.88 | 0.00 | 37.32 |
| ALP143 | 626036256.3 | 8726065.4 | 229666490.2 | 12590406.6 | 437 | 18 | 30 | 23 | 3211.60 | 15.73 | 4.03 | 33.64 |
| ALP144 | 559889261.4 | 35445732.2 | 249429725.9 | 11247209.8 | 389 | 17 | 27 | 18 | 2014.50 | 4.22 | 3.75 | 22.34 |
| ALP145 | 423683637.5 | 16216484.4 | 120285910.4 | 17695784.9 | 405 | 15 | 28 | 25 | 4712.56 | 7.00 | 7.06 | 27.29 |
| ALP146 | 496832185.6 | 20922855.4 | 133470085.6 | 20402316.3 | 404 | 19 | 21 | 23 | 3088.62 | 8.71 | 3.88 | 46.68 |
| ALP147 | 549512446.6 | 25568071.8 | 187686392.7 | 22940134.9 | 419 | 16 | 25 | 29 | 3849.56 | 5.92 | 3.79 | 80.87 |
| ALP148 | 577532067.4 | 26713614.9 | 267670991.4 | 11473631.4 | 404 | 16 | 27 | 18 | 1481.94 | 5.27 | 3.09 | 26.84 |
| ALP149 | 441119841.4 | 14061098.6 | 128977105.3 | 19703410.3 | 411 | 17 | 27 | 27 | 3504.06 | 7.37 | 3.87 | 42.96 |
| ALP150 | 402791608.0 | 18185706.9 | 107516301.2 | 14128494.9 | 434 | 21 | 31 | 30 | 5490.34 | 10.94 | 8.33 | 72.39 |
| ALP151 | 271796407.2 | 10044730.9 | 59835109.9 | 9043625.0 | 400 | 18 | 29 | 19 | 3414.48 | 15.00 | 4.48 | 24.29 |
| ALP152 | 325627628.1 | 5406036.9 | 40769366.5 | 17748914.7 | 377 | 14 | 21 | 26 | 4923.16 | 15.18 | 2.85 | 40.95 |
| ALP154 | 309662032.0 | 5496238.6 | 87519472.8 | 24822700.1 | 376 | 15 | 25 | 26 | 4895.61 | 4.44 | 3.95 | 41.02 |
| ALP155 | 351695265.2 | 9862431.8 | 68082993.0 | 21167193.1 | 412 | 20 | 30 | 28 | 3919.42 | 27.28 | 3.89 | 44.96 |
| ALP156 | 382155261.6 | 11794647.7 | 111385036.9 | 8446849.3 | 380 | 20 | 23 | 26 | 3406.17 | 21.32 | 2.48 | 84.69 |
| ALP157 | 261769826.8 | 6646845.1 | 53405383.1 | 17802160.3 | 354 | 12 | 19 | 25 | 3867.76 | 4.67 | 4.36 | 29.55 |
| ALP158 | 365469288.4 | 10704737.0 | 78038358.0 | 12393659.0 | 392 | 17 | 23 | 18 | 4691.04 | 16.03 | 1.85 | 43.64 |
| ALP159 | 186505864.0 | 14725168.7 | 17730303.9 | 9885056.2 | 371 | 23 | 18 | 20 | 7434.42 | 13.03 | 1.73 | 40.08 |
| ALP160 | 490867415.5 | 13076548.7 | 117201482.2 | 19321421.5 | 396 | 17 | 30 | 24 | 5478.88 | 11.65 | 6.40 | 104.53 |
| ALP161 | 424811664.0 | 26924928.2 | 167108882.6 | 11118367.5 | 387 | 13 | 21 | 22 | 2493.71 | 5.39 | 2.59 | 43.34 |
| ALP162 | 451619140.7 | 50687685.7 | 145850969.3 | 38351784.6 | 389 | 19 | 25 | 29 | 3130.33 | 5.08 | 2.33 | 51.27 |
| ALP163 | 607565823.6 | 38640936.5 | 215120726.6 | 20048606.3 | 417 | 21 | 29 | 23 | 3870.03 | 9.65 | 4.65 | 31.09 |
| ALP165 | 757539888.0 | 35469956.1 | 253624679.8 | 18236654.0 | 408 | 22 | 21 | 26 | 2609.21 | 10.05 | 2.08 | 35.37 |
| ALP166 | 602066044.7 | 20372003.5 | 237967852.6 | 17204047.1 | 419 | 17 | 26 | 22 | 2686.53 | 5.36 | 3.87 | 29.27 |
| ALP167 | 429213267.6 | 15180678.9 | 101888087.6 | 22653484.4 | 438 | 21 | 26 | 28 | 7296.20 | 18.37 | 6.23 | 54.67 |
| ALP168 | 348639883.0 | 33667723.5 | 82238315.4 | 16351291.3 | 396 | 20 | 21 | 21 | 3670.87 | 5.79 | 2.19 | 27.08 |
| ALP169 | 494965001.3 | 21326243.1 | 139294142.7 | 21211537.9 | 425 | 20 | 28 | 22 | 5980.33 | 10.71 | 8.40 | 43.53 |
| ALP170 | 441973909.8 | 11551218.5 | 104773405.4 | 11444522.0 | 405 | 13 | 22 | 26 | 5454.79 | 12.11 | 5.24 | 82.01 |
| ALP171 | 316526681.9 | 7753977.0 | 47290736.5 | 18119768.1 | 371 | 17 | 24 | 27 | 4542.62 | 20.08 | 4.22 | 63.34 |
| ALP172 | 587248973.7 | 8079535.0 | 239083322.7 | 18704342.7 | 415 | 22 | 25 | 21 | 2376.66 | 29.39 | 2.49 | 20.57 |
| ALP173 | 658088936.9 | 12180737.6 | 285065926.1 | 29737087.7 | 415 | 19 | 26 | 25 | 1925.23 | 9.15 | 2.06 | 44.27 |

**Table S3.** Measured chemical properties from R package *rcdk* 3.8.1 (Guha 2007) and their possible functional role in plants.

| **Molecular properties** | **Name** | **Description** | **Possible functional role** | **References** |
| --- | --- | --- | --- | --- |
| MW | Molecular weight | Molecular weight quantified in Daltons (Da). | It is suspected to be related to leaf longevity because peptides and lignans which have the greatest MW, function as long-term physical and storage structures. | (Liu, Luo & Zheng 2018; Walker *et al.* 2023) |
| nAtomP | Number of atoms in the largest conjugated π-system | Number of atoms in the largest aromatic, conjugated π-system. | The largest aromatic, conjugated π-system is high in pigments and light-absorbing molecules which protect plants from ultraviolet radiation. | (Tegelberg & Julkunen‐Tiitto 2001; Treutter 2006; Hoober, Eggink & Chen 2007; Maia *et al.* 2021) |
| ALogP | Hydrophobicity | Hydrophobicity is measured as a log-ratio of solubility in octanol versus water. | May be involved to plant-defense spectrum defined by unsaturated, aromatic, and non-polar metabolites versus saturated and polar metabolites. ALogP is negatively correlated with polarity and subsequently, desiccation resistance. | (Ghose & Crippen 1987; Hoekstra, Golovina & Buitink 2001; Walker *et al.* 2023) |
| TopoPSA | Topological surface area | Topological polar surface area, the sum of surface area of polar atoms in Ångstroms (Å) | Considered to contribute to desiccation resistance but is negatively correlated with passive transport through cell membranes. | (Palm *et al.* 1997; Ertl, Rohde & Selzer 2000; Hoekstra, Golovina & Buitink 2001) |
| Fsp3 | Ratio of carbon atoms with sp3 electron orbits | The fraction of carbon atoms with sp3 electron orbits (i.e. only double and triple bonds) to the total number of carbon atoms. | Fsp3 is positively correlated with melting point, solubility, and the likelihood of a compound to exhibit bioactivity in pharmaceutical assays. | (Lovering, Bikker & Humblet 2009) |

**Table S4.** Studied plants and their structural traits (all metabolites = AM, flavonoids = flav., salicinoids = sali., and primary metabolites = PM) including the molecular weight quantified in Daltons (MW). the number of atoms in the largest aromatic. conjugated π-system (nAtomP). the hydrophobicity is measured as a log-ratio of solubility in octanol versus water (ALogP). the topological polar surface area. the sum of surface area of polar atoms in Ångstroms (TopoPSA) and the fraction of carbon atoms with sp3 electron orbits (Fsp3).

| **Tree** | **MW** | | | | **nAtomP** | | | | **TopoPSA** | | | | **Fsp3** | | | | **ALogP** | | | |
| --- | --- | --- | --- | --- | --- | --- | --- | --- | --- | --- | --- | --- | --- | --- | --- | --- | --- | --- | --- | --- |
|  | **AM** | **flav.** | **sali.** | **PM** | **AM** | **flav.** | **sali.** | **PM** | **AM** | **flav.** | **sali.** | **PM** | **AM** | **flav.** | **sali.** | **PM** | **AM** | **flav.** | **sali.** | **PM** |
| ALP001 | 304.65 | 9.75 | 110.87 | 339.75 | 0.44 | 0.24 | 520.08 | 1.10 | 13.34 | 203.74 | 0.23 | 165.96 | 2.45 | 427.41 | 10.01 | 0.92 | 143.81 | 0.56 | 1.31 | -2.69 |
| ALP002 | 282.03 | 9.00 | 103.32 | 336.58 | 0.42 | 0.10 | 541.59 | 0.85 | 12.40 | 206.40 | 0.22 | 168.98 | 2.78 | 400.15 | 9.81 | 0.93 | 140.77 | 0.57 | 0.95 | -2.96 |
| ALP003 | 317.61 | 10.63 | 116.08 | 342.43 | 0.43 | 0.45 | 512.49 | 1.20 | 14.72 | 201.69 | 0.24 | 164.15 | 2.22 | 421.44 | 9.43 | 0.91 | 148.07 | 0.56 | 0.85 | -2.44 |
| ALP004 | 307.61 | 9.47 | 115.76 | 370.05 | 0.39 | 0.44 | 479.64 | 2.72 | 18.41 | 203.80 | 0.24 | 138.77 | 1.42 | 441.04 | 7.36 | 0.86 | 166.46 | 0.50 | -0.40 | -0.39 |
| ALP005 | 300.41 | 9.81 | 108.49 | 348.39 | 0.43 | 0.53 | 522.22 | 1.86 | 14.64 | 206.04 | 0.24 | 150.82 | 2.27 | 413.92 | 10.83 | 0.86 | 146.26 | 0.61 | 1.32 | -1.35 |
| ALP006 | 299.28 | 10.23 | 108.50 | 344.02 | 0.41 | 0.31 | 508.43 | 0.99 | 16.68 | 207.20 | 0.24 | 165.49 | 1.91 | 426.12 | 10.67 | 0.93 | 146.57 | 0.59 | 1.43 | -2.60 |
| ALP010 | 308.49 | 9.46 | 114.40 | 360.32 | 0.38 | 0.58 | 474.79 | 2.96 | 17.39 | 199.36 | 0.25 | 145.14 | 1.40 | 462.01 | 7.35 | 0.85 | 173.33 | 0.50 | -0.38 | -0.26 |
| ALP011 | 327.40 | 9.85 | 118.60 | 366.33 | 0.36 | 0.77 | 479.99 | 4.02 | 19.14 | 202.70 | 0.26 | 156.93 | 1.27 | 488.88 | 7.16 | 0.81 | 182.58 | 0.49 | -0.41 | -0.35 |
| ALP012 | 342.16 | 9.88 | 120.15 | 361.01 | 0.36 | 0.96 | 491.32 | 4.63 | 15.66 | 198.72 | 0.24 | 153.52 | 1.88 | 497.39 | 7.09 | 0.79 | 184.99 | 0.49 | -0.39 | -0.05 |
| ALP014 | 254.74 | 8.06 | 97.57 | 333.86 | 0.39 | 0.07 | 534.23 | 1.02 | 11.46 | 209.00 | 0.24 | 167.57 | 2.56 | 409.90 | 9.94 | 0.92 | 142.11 | 0.57 | 1.09 | -2.88 |
| ALP015 | 244.70 | 8.23 | 94.80 | 332.91 | 0.39 | 0.05 | 526.95 | 1.33 | 12.57 | 207.36 | 0.25 | 156.70 | 2.34 | 396.14 | 9.34 | 0.90 | 138.45 | 0.55 | 0.81 | -2.04 |
| ALP016 | 261.30 | 8.13 | 99.32 | 324.22 | 0.40 | 0.16 | 529.97 | 1.28 | 11.91 | 206.48 | 0.24 | 157.28 | 2.51 | 416.53 | 8.21 | 0.90 | 150.10 | 0.51 | 0.18 | -2.37 |
| ALP017 | 304.74 | 9.57 | 113.92 | 338.46 | 0.46 | 0.25 | 520.18 | 0.81 | 14.79 | 206.92 | 0.24 | 172.85 | 2.21 | 379.89 | 9.10 | 0.94 | 136.10 | 0.56 | 0.57 | -3.23 |
| ALP019 | 313.53 | 10.24 | 118.46 | 333.49 | 0.44 | 0.43 | 525.08 | 1.44 | 14.09 | 204.74 | 0.22 | 158.04 | 2.50 | 395.48 | 8.05 | 0.89 | 146.04 | 0.53 | 0.01 | -2.17 |
| ALP020 | 296.07 | 10.10 | 106.17 | 358.49 | 0.41 | 0.38 | 523.31 | 2.89 | 14.89 | 202.77 | 0.25 | 147.59 | 2.19 | 400.42 | 11.00 | 0.81 | 145.91 | 0.63 | 1.20 | -0.89 |
| ALP021 | 329.59 | 9.98 | 122.98 | 357.08 | 0.40 | 0.56 | 499.73 | 3.61 | 16.90 | 206.18 | 0.26 | 153.63 | 1.49 | 452.77 | 7.25 | 0.83 | 170.69 | 0.49 | -0.45 | -0.48 |
| ALP022 | 317.03 | 9.62 | 117.63 | 356.44 | 0.38 | 0.56 | 469.27 | 2.88 | 14.43 | 185.39 | 0.24 | 152.79 | 2.02 | 455.46 | 7.45 | 0.85 | 170.21 | 0.49 | -0.35 | -0.79 |
| ALP023 | 255.58 | 7.43 | 99.95 | 351.12 | 0.45 | -0.02 | 533.67 | 2.11 | 10.05 | 208.70 | 0.21 | 156.36 | 2.84 | 400.42 | 11.00 | 0.85 | 145.91 | 0.63 | 1.20 | -1.66 |
| ALP024 | 339.26 | 10.27 | 117.75 | 368.98 | 0.35 | 1.09 | 487.65 | 4.75 | 18.25 | 200.10 | 0.25 | 160.97 | 1.54 | 525.50 | 7.20 | 0.77 | 193.94 | 0.48 | -0.31 | -0.15 |
| ALP025 | 353.35 | 9.78 | 123.32 | 371.66 | 0.36 | 1.06 | 494.43 | 5.62 | 12.21 | 189.71 | 0.23 | 158.38 | 2.44 | 468.61 | 7.13 | 0.73 | 175.60 | 0.49 | -0.44 | 0.46 |
| ALP026 | 276.75 | 7.95 | 102.48 | 335.04 | 0.41 | 0.26 | 543.41 | 2.40 | 11.84 | 210.44 | 0.24 | 154.62 | 2.63 | 420.22 | 7.16 | 0.84 | 160.79 | 0.50 | -0.56 | -1.54 |
| ALP027 | 250.80 | 8.07 | 94.96 | 328.43 | 0.40 | 0.09 | 529.77 | 1.44 | 13.07 | 208.90 | 0.25 | 160.74 | 2.26 | 423.14 | 8.01 | 0.90 | 155.75 | 0.51 | 0.00 | -2.50 |
| ALP028 | 341.98 | 10.09 | 117.22 | 362.85 | 0.34 | 1.13 | 467.23 | 4.90 | 15.40 | 185.24 | 0.22 | 158.97 | 2.07 | 508.61 | 7.28 | 0.78 | 187.95 | 0.48 | -0.31 | -0.14 |
| ALP029 | 296.55 | 9.51 | 109.71 | 349.89 | 0.40 | 0.11 | 542.84 | 1.12 | 14.14 | 210.87 | 0.27 | 162.53 | 2.15 | 454.06 | 9.98 | 0.91 | 157.25 | 0.57 | 1.16 | -2.25 |
| ALP030 | 293.95 | 9.48 | 108.66 | 346.21 | 0.39 | 0.19 | 541.14 | 1.16 | 14.83 | 213.50 | 0.27 | 164.83 | 2.04 | 418.78 | 9.64 | 0.91 | 141.55 | 0.55 | 1.13 | -2.54 |
| ALP032 | 245.28 | 8.05 | 91.44 | 325.89 | 0.40 | 0.27 | 533.99 | 1.84 | 10.93 | 208.74 | 0.23 | 151.17 | 2.66 | 431.35 | 8.91 | 0.87 | 156.25 | 0.52 | 0.40 | -1.85 |
| ALP033 | 267.50 | 9.37 | 100.25 | 325.12 | 0.36 | 0.15 | 520.92 | 1.63 | 14.61 | 206.67 | 0.25 | 154.18 | 2.14 | 418.18 | 8.03 | 0.90 | 152.07 | 0.52 | 0.10 | -2.00 |
| ALP034 | 323.96 | 9.72 | 119.71 | 368.26 | 0.38 | 0.67 | 486.02 | 4.59 | 13.57 | 189.01 | 0.24 | 153.13 | 2.16 | 472.63 | 7.15 | 0.78 | 177.31 | 0.49 | -0.44 | 0.19 |
| ALP035 | 231.94 | 8.36 | 85.28 | 329.15 | 0.36 | -0.02 | 529.27 | 1.82 | 11.61 | 206.47 | 0.22 | 153.84 | 2.65 | 405.11 | 8.71 | 0.87 | 138.60 | 0.52 | 0.70 | -2.03 |
| ALP036 | 232.22 | 7.80 | 88.25 | 327.19 | 0.39 | -0.10 | 545.52 | 1.27 | 9.91 | 211.13 | 0.21 | 158.36 | 2.97 | 470.82 | 10.11 | 0.90 | 147.71 | 0.53 | 1.83 | -2.43 |
| ALP037 | 339.39 | 10.14 | 117.28 | 371.86 | 0.36 | 1.03 | 487.41 | 5.41 | 18.65 | 199.80 | 0.28 | 154.29 | 1.28 | 526.03 | 7.10 | 0.75 | 194.48 | 0.49 | -0.35 | 0.51 |
| ALP038 | 243.24 | 7.75 | 92.43 | 338.19 | 0.40 | 0.05 | 545.16 | 1.22 | 10.33 | 213.20 | 0.22 | 161.93 | 2.82 | 475.43 | 8.75 | 0.91 | 165.45 | 0.52 | 0.69 | -2.40 |
| ALP039 | 318.95 | 9.74 | 110.95 | 360.78 | 0.36 | 0.84 | 470.61 | 4.31 | 12.39 | 183.02 | 0.23 | 155.58 | 2.23 | 508.31 | 7.09 | 0.79 | 188.54 | 0.49 | -0.38 | -0.20 |
| ALP040 | 270.27 | 8.26 | 99.72 | 337.17 | 0.41 | 0.49 | 529.58 | 2.18 | 10.38 | 205.74 | 0.22 | 151.24 | 2.76 | 445.17 | 7.80 | 0.85 | 164.03 | 0.47 | -0.18 | -1.43 |
| ALP041 | 245.52 | 7.92 | 92.70 | 345.35 | 0.39 | 0.05 | 517.51 | 1.99 | 11.42 | 203.78 | 0.23 | 152.77 | 2.48 | 405.48 | 9.95 | 0.86 | 141.80 | 0.58 | 1.04 | -1.46 |
| ALP042 | 246.65 | 8.33 | 94.36 | 331.35 | 0.39 | 0.04 | 511.56 | 1.26 | 13.15 | 203.61 | 0.23 | 159.77 | 2.34 | 420.99 | 8.00 | 0.91 | 155.58 | 0.50 | -0.06 | -2.35 |
| ALP043 | 241.63 | 8.29 | 91.17 | 328.38 | 0.38 | 0.06 | 519.56 | 1.49 | 11.62 | 203.76 | 0.23 | 157.31 | 2.55 | 448.44 | 10.39 | 0.89 | 147.14 | 0.56 | 1.63 | -2.18 |
| ALP044 | 239.59 | 8.01 | 90.57 | 326.64 | 0.39 | 0.07 | 526.97 | 1.28 | 9.78 | 203.45 | 0.21 | 160.73 | 2.92 | 430.31 | 7.57 | 0.90 | 161.60 | 0.50 | -0.32 | -2.49 |
| ALP045 | 338.79 | 9.86 | 116.03 | 375.50 | 0.34 | 1.11 | 470.51 | 5.90 | 18.39 | 189.88 | 0.25 | 158.73 | 1.55 | 499.23 | 7.18 | 0.72 | 185.75 | 0.49 | -0.38 | 0.63 |
| ALP046 | 341.62 | 9.90 | 119.08 | 375.67 | 0.38 | 1.05 | 483.27 | 5.07 | 12.42 | 188.31 | 0.23 | 154.95 | 2.31 | 502.88 | 7.13 | 0.75 | 186.74 | 0.49 | -0.37 | 0.49 |
| ALP047 | 353.12 | 10.65 | 119.77 | 386.19 | 0.35 | 1.24 | 495.08 | 6.04 | 14.10 | 192.13 | 0.27 | 162.98 | 1.82 | 523.17 | 7.13 | 0.71 | 193.39 | 0.48 | -0.34 | 0.69 |
| ALP048 | 320.83 | 9.99 | 116.57 | 366.43 | 0.36 | 0.73 | 488.91 | 4.40 | 16.46 | 201.52 | 0.24 | 155.61 | 1.67 | 504.19 | 7.17 | 0.79 | 187.18 | 0.49 | -0.36 | -0.09 |
| ALP050 | 254.97 | 7.81 | 87.85 | 349.68 | 0.39 | 0.43 | 431.38 | 1.33 | 10.28 | 163.05 | 0.20 | 152.68 | 2.57 | 400.42 | 11.00 | 0.87 | 145.91 | 0.63 | 1.20 | -1.49 |
| ALP051 | 242.25 | 7.73 | 88.05 | 337.65 | 0.40 | 0.02 | 445.89 | 1.13 | 11.48 | 169.16 | 0.23 | 155.65 | 2.30 | 400.42 | 11.00 | 0.90 | 145.91 | 0.63 | 1.20 | -2.00 |
| ALP052 | 325.83 | 10.00 | 117.23 | 374.48 | 0.36 | 0.72 | 412.49 | 5.86 | 18.51 | 161.98 | 0.23 | 162.71 | 1.50 | 491.96 | 7.30 | 0.70 | 182.83 | 0.48 | -0.35 | 0.38 |
| ALP053 | 330.31 | 10.46 | 117.62 | 386.41 | 0.33 | 0.88 | 493.99 | 6.16 | 18.69 | 191.22 | 0.32 | 163.47 | 1.13 | 511.75 | 7.24 | 0.69 | 189.13 | 0.48 | -0.31 | 0.76 |
| ALP054 | 284.49 | 9.69 | 103.24 | 358.74 | 0.37 | 0.21 | 519.00 | 1.68 | 16.75 | 204.54 | 0.31 | 161.31 | 1.47 | 422.14 | 7.62 | 0.88 | 157.75 | 0.50 | -0.25 | -1.68 |
| ALP055 | 251.26 | 8.33 | 91.72 | 350.96 | 0.38 | 0.24 | 528.31 | 1.60 | 10.58 | 204.86 | 0.22 | 159.22 | 2.79 | 416.98 | 8.35 | 0.88 | 142.24 | 0.51 | 0.59 | -1.86 |
| ALP056 | 338.26 | 10.08 | 122.06 | 381.26 | 0.36 | 0.88 | 470.31 | 5.90 | 19.27 | 201.75 | 0.25 | 162.62 | 1.29 | 470.21 | 7.18 | 0.73 | 176.53 | 0.49 | -0.44 | 0.53 |
| ALP057 | 319.82 | 10.26 | 113.01 | 349.34 | 0.44 | 0.43 | 517.89 | 1.13 | 13.57 | 201.06 | 0.23 | 164.89 | 2.42 | 436.21 | 9.51 | 0.92 | 145.94 | 0.54 | 1.17 | -2.38 |
| ALP058 | 299.85 | 9.89 | 107.87 | 341.72 | 0.43 | 0.40 | 509.35 | 1.22 | 15.35 | 202.49 | 0.26 | 166.41 | 1.92 | 415.17 | 7.71 | 0.92 | 153.33 | 0.51 | -0.11 | -2.61 |
| ALP059 | 301.41 | 10.12 | 109.66 | 345.86 | 0.40 | 0.35 | 522.15 | 1.28 | 15.85 | 206.38 | 0.26 | 165.47 | 2.00 | 451.53 | 7.92 | 0.91 | 159.60 | 0.45 | 0.24 | -2.38 |
| ALP060 | 283.13 | 9.86 | 101.49 | 347.94 | 0.39 | 0.40 | 513.22 | 1.64 | 14.34 | 199.98 | 0.24 | 155.58 | 2.24 | 440.30 | 8.33 | 0.88 | 160.48 | 0.53 | 0.21 | -1.61 |
| ALP061 | 360.57 | 9.39 | 128.54 | 390.28 | 0.38 | 0.73 | 485.85 | 6.94 | 18.06 | 202.39 | 0.27 | 163.52 | 1.28 | 494.43 | 7.85 | 0.67 | 174.79 | 0.45 | 0.24 | 1.13 |
| ALP062 | 353.88 | 10.49 | 123.17 | 389.32 | 0.36 | 1.10 | 461.31 | 6.60 | 20.66 | 200.00 | 0.25 | 163.17 | 1.07 | 504.41 | 7.24 | 0.69 | 185.86 | 0.48 | -0.28 | 0.97 |
| ALP063 | 354.54 | 10.49 | 122.03 | 384.98 | 0.36 | 1.20 | 466.60 | 6.80 | 21.17 | 204.58 | 0.25 | 160.34 | 0.99 | 522.21 | 7.14 | 0.68 | 192.72 | 0.48 | -0.33 | 1.11 |
| ALP064 | 353.51 | 9.75 | 121.04 | 397.97 | 0.35 | 1.12 | 500.34 | 7.22 | 19.67 | 203.23 | 0.31 | 159.41 | 1.12 | 476.19 | 7.24 | 0.65 | 177.47 | 0.49 | -0.37 | 1.67 |
| ALP065 | 365.91 | 9.81 | 125.50 | 391.27 | 0.36 | 1.16 | 499.86 | 7.16 | 20.10 | 204.09 | 0.31 | 161.00 | 1.05 | 486.04 | 7.31 | 0.66 | 179.84 | 0.48 | -0.30 | 1.38 |
| ALP066 | 335.56 | 9.72 | 122.96 | 391.91 | 0.40 | 0.75 | 474.14 | 6.09 | 20.81 | 203.25 | 0.27 | 168.95 | 0.99 | 462.04 | 7.16 | 0.72 | 174.06 | 0.49 | -0.47 | 0.43 |
| ALP067 | 261.42 | 9.19 | 94.87 | 346.06 | 0.35 | 0.48 | 515.20 | 1.20 | 14.94 | 205.55 | 0.28 | 164.87 | 1.79 | 409.14 | 7.83 | 0.91 | 151.05 | 0.52 | -0.07 | -2.40 |
| ALP068 | 265.99 | 9.15 | 99.30 | 339.93 | 0.36 | 0.15 | 485.71 | 1.03 | 12.87 | 190.19 | 0.24 | 166.98 | 2.23 | 410.40 | 7.85 | 0.92 | 135.28 | 0.47 | 0.58 | -2.73 |
| ALP069 | 254.46 | 8.15 | 94.75 | 339.30 | 0.42 | 0.36 | 503.65 | 1.42 | 13.16 | 199.18 | 0.25 | 160.29 | 2.15 | 343.54 | 7.42 | 0.89 | 125.99 | 0.51 | -0.20 | -2.22 |
| ALP070 | 245.41 | 8.37 | 91.68 | 342.31 | 0.38 | 0.20 | 492.87 | 1.27 | 12.70 | 193.34 | 0.24 | 166.13 | 2.22 | 376.20 | 8.74 | 0.91 | 134.28 | 0.54 | 0.45 | -2.49 |
| ALP071 | 281.41 | 9.28 | 103.49 | 346.59 | 0.37 | 0.61 | 522.26 | 1.32 | 14.15 | 207.52 | 0.27 | 164.34 | 1.99 | 426.90 | 7.76 | 0.90 | 158.61 | 0.48 | -0.23 | -2.34 |
| ALP072 | 334.33 | 10.12 | 125.32 | 382.51 | 0.38 | 0.57 | 465.64 | 4.38 | 19.92 | 198.80 | 0.25 | 170.64 | 1.20 | 455.66 | 7.99 | 0.79 | 163.64 | 0.45 | 0.11 | -0.65 |
| ALP073 | 360.05 | 9.99 | 127.36 | 386.00 | 0.38 | 0.90 | 507.73 | 6.18 | 21.17 | 216.83 | 0.31 | 167.13 | 0.70 | 503.89 | 7.48 | 0.71 | 182.61 | 0.47 | -0.07 | 0.47 |
| ALP074 | 349.56 | 9.96 | 123.41 | 385.44 | 0.38 | 0.99 | 498.98 | 5.92 | 21.09 | 212.97 | 0.30 | 167.60 | 0.80 | 492.49 | 7.29 | 0.72 | 182.03 | 0.48 | -0.29 | 0.32 |
| ALP075 | 365.61 | 10.14 | 126.40 | 393.70 | 0.37 | 1.09 | 491.88 | 7.36 | 20.90 | 207.22 | 0.30 | 166.00 | 0.84 | 504.93 | 7.46 | 0.66 | 183.70 | 0.47 | -0.11 | 1.21 |
| ALP076 | 346.44 | 9.60 | 128.17 | 390.31 | 0.41 | 0.61 | 513.12 | 5.93 | 20.79 | 217.32 | 0.33 | 166.37 | 0.69 | 469.18 | 7.44 | 0.72 | 172.04 | 0.47 | -0.17 | 0.46 |
| ALP077 | 266.62 | 8.01 | 100.06 | 339.91 | 0.44 | 0.43 | 514.67 | 1.19 | 11.95 | 199.09 | 0.23 | 167.10 | 2.50 | 412.51 | 7.27 | 0.91 | 154.32 | 0.50 | -0.37 | -2.68 |
| ALP078 | 317.52 | 7.67 | 100.87 | 328.99 | 0.47 | 1.44 | 536.14 | 2.57 | 12.68 | 208.00 | 0.25 | 65.15 | 2.40 | 413.39 | 7.11 | 0.94 | 156.35 | 0.50 | -0.48 | 3.78 |
| ALP079 | 287.37 | 8.65 | 101.65 | 365.40 | 0.40 | 0.46 | 513.49 | 1.81 | 12.02 | 200.29 | 0.22 | 161.38 | 2.54 | 434.10 | 7.87 | 0.87 | 161.61 | 0.49 | -0.19 | -1.61 |
| ALP080 | 248.88 | 7.97 | 91.72 | 354.56 | 0.39 | 0.15 | 535.56 | 1.28 | 12.10 | 204.86 | 0.23 | 168.35 | 2.70 | 402.23 | 7.92 | 0.91 | 149.16 | 0.52 | -0.05 | -2.44 |
| ALP081 | 359.15 | 10.10 | 124.20 | 386.00 | 0.38 | 1.18 | 479.35 | 6.20 | 20.67 | 206.06 | 0.27 | 166.63 | 0.96 | 489.61 | 7.11 | 0.71 | 182.79 | 0.49 | -0.42 | 0.53 |
| ALP082 | 281.79 | 8.96 | 105.69 | 348.53 | 0.40 | 0.45 | 501.42 | 1.22 | 14.15 | 199.17 | 0.25 | 170.75 | 2.09 | 406.70 | 7.50 | 0.92 | 149.78 | 0.51 | -0.17 | -2.70 |
| ALP083 | 261.37 | 8.59 | 98.81 | 344.14 | 0.37 | 0.18 | 500.26 | 1.21 | 13.83 | 195.57 | 0.26 | 166.87 | 2.04 | 417.94 | 7.98 | 0.92 | 146.69 | 0.50 | 0.29 | -2.54 |
| ALP085 | 338.58 | 10.72 | 116.03 | 372.11 | 0.35 | 1.12 | 467.83 | 5.22 | 18.53 | 188.64 | 0.28 | 152.60 | 1.27 | 532.13 | 7.41 | 0.74 | 195.40 | 0.48 | -0.22 | 0.57 |
| ALP086 | 331.15 | 10.12 | 120.44 | 385.55 | 0.39 | 0.72 | 466.36 | 4.55 | 16.43 | 190.69 | 0.27 | 156.36 | 1.43 | 478.82 | 7.42 | 0.77 | 177.98 | 0.48 | -0.31 | 0.37 |
| ALP087 | 337.93 | 10.13 | 118.24 | 380.61 | 0.36 | 1.01 | 476.24 | 5.58 | 17.51 | 197.00 | 0.27 | 160.79 | 1.32 | 505.24 | 7.21 | 0.73 | 187.50 | 0.49 | -0.35 | 0.51 |
| ALP088 | 347.09 | 10.42 | 120.01 | 368.09 | 0.36 | 1.10 | 471.38 | 4.80 | 16.92 | 193.12 | 0.26 | 160.85 | 1.47 | 509.91 | 7.31 | 0.77 | 188.08 | 0.48 | -0.28 | -0.14 |
| ALP089 | 344.77 | 10.08 | 121.56 | 385.06 | 0.37 | 0.98 | 450.13 | 6.35 | 20.49 | 194.09 | 0.24 | 160.28 | 1.17 | 489.36 | 7.22 | 0.70 | 182.33 | 0.49 | -0.38 | 0.93 |
| ALP090 | 349.16 | 10.14 | 120.27 | 390.70 | 0.36 | 1.12 | 475.95 | 6.69 | 16.83 | 194.23 | 0.26 | 162.30 | 1.51 | 495.47 | 7.19 | 0.68 | 184.38 | 0.49 | -0.38 | 1.09 |
| ALP091 | 356.94 | 10.16 | 120.70 | 381.48 | 0.37 | 1.26 | 476.50 | 5.40 | 16.86 | 194.58 | 0.26 | 162.78 | 1.56 | 487.43 | 7.16 | 0.73 | 181.79 | 0.49 | -0.39 | 0.33 |
| ALP092 | 328.33 | 9.49 | 125.55 | 382.48 | 0.42 | 0.38 | 490.22 | 4.93 | 20.87 | 210.95 | 0.28 | 165.83 | 0.87 | 439.04 | 7.88 | 0.76 | 159.34 | 0.46 | 0.00 | -0.17 |
| ALP093 | 249.53 | 8.65 | 92.90 | 335.17 | 0.35 | 0.11 | 518.06 | 1.94 | 12.79 | 200.35 | 0.25 | 161.53 | 2.34 | 394.48 | 7.25 | 0.87 | 146.58 | 0.50 | -0.32 | -2.41 |
| ALP094 | 351.42 | 10.09 | 125.66 | 387.04 | 0.39 | 0.91 | 481.67 | 5.54 | 19.52 | 201.18 | 0.29 | 165.10 | 1.04 | 488.73 | 7.17 | 0.73 | 182.24 | 0.49 | -0.39 | 0.32 |
| ALP095 | 296.87 | 9.15 | 106.29 | 356.82 | 0.39 | 0.70 | 489.23 | 3.00 | 13.64 | 194.78 | 0.23 | 162.91 | 2.15 | 488.59 | 7.32 | 0.83 | 179.49 | 0.48 | -0.23 | -1.37 |
| ALP096 | 346.81 | 10.21 | 120.53 | 391.62 | 0.37 | 1.06 | 477.47 | 6.22 | 17.25 | 197.42 | 0.26 | 163.49 | 1.43 | 501.29 | 7.16 | 0.70 | 186.50 | 0.49 | -0.38 | 0.85 |
| ALP097 | 273.92 | 8.89 | 100.00 | 341.95 | 0.40 | 0.56 | 503.84 | 1.47 | 14.83 | 202.09 | 0.26 | 166.50 | 1.88 | 420.53 | 7.46 | 0.91 | 155.95 | 0.49 | -0.27 | -2.54 |
| ALP099 | 355.12 | 9.89 | 122.79 | 382.58 | 0.37 | 1.05 | 493.73 | 5.69 | 17.97 | 201.85 | 0.30 | 169.17 | 1.17 | 495.25 | 7.23 | 0.73 | 182.81 | 0.48 | -0.29 | 0.09 |
| ALP100 | 350.24 | 9.72 | 127.07 | 381.78 | 0.41 | 0.70 | 492.08 | 5.06 | 19.16 | 206.40 | 0.29 | 165.57 | 1.10 | 481.77 | 7.32 | 0.76 | 178.14 | 0.48 | -0.28 | 0.00 |
| ALP101 | 256.10 | 8.36 | 93.18 | 340.77 | 0.39 | 0.36 | 529.58 | 1.44 | 12.43 | 207.75 | 0.25 | 164.45 | 2.32 | 406.19 | 7.32 | 0.90 | 156.50 | 0.52 | -0.52 | -2.45 |
| ALP102 | 367.56 | 10.02 | 127.94 | 389.94 | 0.38 | 0.93 | 483.47 | 6.94 | 20.45 | 203.76 | 0.28 | 165.60 | 0.96 | 510.74 | 7.54 | 0.68 | 184.55 | 0.47 | -0.02 | 1.01 |
| ALP103 | 340.20 | 9.55 | 121.73 | 387.08 | 0.38 | 0.68 | 511.02 | 6.01 | 20.35 | 213.95 | 0.32 | 162.26 | 0.79 | 487.92 | 7.59 | 0.71 | 175.90 | 0.46 | 0.01 | 0.67 |
| ALP104 | 250.12 | 8.40 | 91.53 | 346.43 | 0.38 | 0.40 | 505.73 | 1.94 | 11.38 | 196.42 | 0.24 | 159.60 | 2.43 | 435.40 | 7.88 | 0.87 | 161.48 | 0.48 | -0.19 | -1.90 |
| ALP105 | 350.03 | 10.08 | 119.34 | 375.57 | 0.37 | 1.16 | 473.48 | 5.83 | 20.21 | 201.22 | 0.27 | 164.49 | 1.11 | 503.23 | 7.14 | 0.72 | 187.08 | 0.49 | -0.38 | 0.24 |
| ALP106 | 253.79 | 8.28 | 90.70 | 351.48 | 0.39 | 0.44 | 490.03 | 1.15 | 12.56 | 192.56 | 0.25 | 163.68 | 2.14 | 387.52 | 8.27 | 0.91 | 148.37 | 0.55 | -0.11 | -2.15 |
| ALP107 | 377.56 | 10.19 | 127.90 | 387.45 | 0.38 | 1.28 | 495.61 | 6.97 | 21.22 | 214.04 | 0.29 | 163.47 | 0.82 | 504.30 | 7.31 | 0.68 | 184.68 | 0.48 | -0.20 | 1.07 |
| ALP110 | 361.15 | 9.87 | 123.94 | 393.86 | 0.36 | 1.07 | 491.02 | 7.33 | 21.30 | 208.91 | 0.30 | 165.36 | 0.84 | 502.35 | 7.48 | 0.66 | 182.25 | 0.47 | -0.08 | 1.23 |
| ALP111 | 367.15 | 10.33 | 124.48 | 398.36 | 0.36 | 1.30 | 481.12 | 7.92 | 21.37 | 206.80 | 0.28 | 164.29 | 0.90 | 499.83 | 7.21 | 0.63 | 184.80 | 0.48 | -0.31 | 1.68 |
| ALP112 | 372.53 | 10.41 | 129.11 | 393.70 | 0.38 | 1.18 | 509.03 | 7.40 | 21.42 | 214.77 | 0.31 | 163.48 | 0.82 | 500.11 | 7.14 | 0.65 | 185.64 | 0.49 | -0.36 | 1.36 |
| ALP113 | 366.19 | 9.87 | 129.72 | 395.37 | 0.39 | 0.91 | 508.31 | 7.23 | 21.01 | 214.31 | 0.31 | 167.30 | 0.83 | 494.01 | 7.45 | 0.67 | 179.29 | 0.47 | -0.08 | 1.09 |
| ALP114 | 349.80 | 9.90 | 128.52 | 390.42 | 0.41 | 0.64 | 479.85 | 5.98 | 21.29 | 201.13 | 0.29 | 167.97 | 0.94 | 477.53 | 7.59 | 0.72 | 173.41 | 0.46 | -0.06 | 0.37 |
| ALP115 | 332.57 | 9.53 | 125.21 | 391.77 | 0.41 | 0.48 | 493.36 | 5.74 | 20.58 | 209.28 | 0.30 | 169.45 | 0.91 | 460.56 | 7.61 | 0.73 | 167.62 | 0.47 | -0.07 | 0.14 |
| ALP116 | 364.07 | 10.09 | 125.46 | 398.58 | 0.38 | 1.09 | 499.07 | 8.00 | 21.09 | 212.70 | 0.29 | 164.18 | 0.84 | 504.28 | 7.28 | 0.63 | 185.36 | 0.48 | -0.24 | 1.70 |
| ALP117 | 374.20 | 10.22 | 127.68 | 399.43 | 0.37 | 1.17 | 482.88 | 8.13 | 21.30 | 211.11 | 0.27 | 164.93 | 0.86 | 506.63 | 7.34 | 0.63 | 185.29 | 0.47 | -0.18 | 1.74 |
| ALP118 | 371.25 | 9.81 | 127.00 | 400.25 | 0.37 | 1.17 | 483.72 | 8.34 | 21.27 | 206.21 | 0.28 | 164.64 | 0.92 | 499.44 | 7.43 | 0.62 | 181.68 | 0.47 | -0.11 | 1.89 |
| ALP119 | 241.75 | 8.93 | 84.38 | 339.32 | 0.33 | 0.50 | 497.72 | 1.55 | 12.04 | 193.71 | 0.23 | 156.06 | 2.38 | 423.51 | 7.78 | 0.88 | 154.81 | 0.50 | -0.02 | -1.96 |
| ALP120 | 267.56 | 9.17 | 94.47 | 337.92 | 0.36 | 0.67 | 519.16 | 1.75 | 14.88 | 204.18 | 0.27 | 157.95 | 1.99 | 435.59 | 7.75 | 0.88 | 163.47 | 0.48 | -0.30 | -2.04 |
| ALP121 | 267.98 | 9.67 | 96.62 | 341.33 | 0.34 | 0.45 | 494.56 | 2.06 | 15.11 | 199.06 | 0.25 | 162.23 | 1.92 | 286.28 | 7.00 | 0.86 | 119.61 | 0.54 | -0.95 | -2.28 |
| ALP122 | 262.98 | 8.73 | 96.19 | 341.48 | 0.39 | 0.45 | 515.79 | 1.52 | 14.16 | 203.56 | 0.26 | 166.23 | 2.00 | 383.79 | 7.36 | 0.89 | 140.44 | 0.50 | -0.20 | -2.59 |
| ALP123 | 342.09 | 9.52 | 126.24 | 388.38 | 0.41 | 0.58 | 516.59 | 6.52 | 21.04 | 213.24 | 0.32 | 161.35 | 0.91 | 478.31 | 7.51 | 0.69 | 174.06 | 0.47 | -0.09 | 0.93 |
| ALP124 | 360.21 | 10.03 | 124.37 | 403.24 | 0.36 | 1.14 | 502.86 | 8.17 | 21.35 | 212.76 | 0.31 | 166.78 | 0.82 | 485.36 | 7.26 | 0.62 | 180.06 | 0.48 | -0.32 | 1.76 |
| ALP125 | 367.14 | 9.96 | 124.49 | 400.51 | 0.38 | 1.22 | 512.45 | 8.07 | 21.19 | 216.82 | 0.32 | 166.05 | 0.68 | 503.44 | 7.38 | 0.63 | 184.53 | 0.47 | -0.19 | 1.69 |
| ALP126 | 371.49 | 10.17 | 123.58 | 402.59 | 0.35 | 1.44 | 458.42 | 8.41 | 21.04 | 194.37 | 0.25 | 166.17 | 1.09 | 490.43 | 7.36 | 0.62 | 180.28 | 0.48 | -0.22 | 1.84 |
| ALP127 | 346.78 | 10.17 | 120.26 | 398.80 | 0.36 | 1.12 | 483.80 | 7.72 | 21.46 | 210.21 | 0.28 | 166.75 | 0.88 | 488.33 | 7.20 | 0.64 | 182.40 | 0.49 | -0.40 | 1.39 |
| ALP128 | 364.30 | 10.26 | 127.85 | 407.71 | 0.38 | 1.05 | 542.37 | 8.51 | 20.85 | 214.16 | 0.36 | 168.92 | 0.80 | 496.85 | 7.26 | 0.61 | 183.91 | 0.48 | -0.31 | 1.85 |
| ALP129 | 371.58 | 10.72 | 128.80 | 415.05 | 0.37 | 1.13 | 546.93 | 9.33 | 21.15 | 224.60 | 0.37 | 157.11 | 0.61 | 507.83 | 7.28 | 0.56 | 186.50 | 0.48 | -0.24 | 3.09 |
| ALP130 | 245.66 | 9.11 | 82.82 | 326.33 | 0.32 | 0.61 | 378.96 | 5.04 | 9.78 | 145.42 | 0.19 | 108.98 | 2.28 | 478.64 | 8.87 | 0.65 | 172.62 | 0.44 | 0.22 | 1.27 |
| ALP131 | 214.84 | 7.55 | 85.01 | 349.22 | 0.36 | -0.25 | 452.16 | 1.66 | 11.29 | 173.98 | 0.23 | 165.10 | 2.23 | 383.67 | 8.63 | 0.89 | 144.27 | 0.49 | -0.08 | -2.32 |
| ALP132 | 318.46 | 9.87 | 114.74 | 354.41 | 0.39 | 0.81 | 437.36 | 2.70 | 18.28 | 170.31 | 0.22 | 159.30 | 1.64 | 530.51 | 8.05 | 0.96 | 186.51 | 0.45 | 0.31 | -1.83 |
| ALP134 | 372.42 | 10.06 | 125.39 | 420.31 | 0.42 | 1.40 | 494.03 | 4.07 | 19.56 | 207.99 | 0.30 | 174.09 | 0.87 | 451.90 | 8.09 | 0.87 | 164.89 | 0.46 | -0.06 | -0.61 |
| ALP135 | 267.41 | 9.39 | 105.51 | 358.92 | 0.42 | -0.26 | 359.90 | 3.07 | 9.66 | 138.00 | 0.19 | 155.01 | 2.25 | 411.70 | 7.06 | 0.91 | 158.75 | 0.50 | -0.63 | -1.35 |
| ALP136 | 361.23 | 10.54 | 127.73 | 358.39 | 0.43 | 1.31 | 454.04 | 3.07 | 20.23 | 192.75 | 0.23 | 156.35 | 1.30 | 437.69 | 7.05 | 0.93 | 166.91 | 0.50 | -0.58 | -1.41 |
| ALP139 | 264.20 | 9.49 | 109.13 | 334.61 | 0.39 | 0.05 | 363.28 | 1.03 | 10.08 | 139.50 | 0.19 | 157.54 | 2.22 | 389.15 | 7.00 | 0.90 | 151.91 | 0.51 | -0.71 | -2.23 |
| ALP140 | 367.27 | 10.40 | 128.61 | 358.43 | 0.43 | 1.36 | 486.73 | 2.70 | 14.53 | 181.76 | 0.24 | 155.18 | 1.82 | 435.28 | 7.04 | 0.92 | 166.04 | 0.50 | -0.58 | -1.32 |
| ALP141 | 307.85 | 9.89 | 115.32 | 335.90 | 0.39 | 0.72 | 453.31 | 1.32 | 13.23 | 185.44 | 0.33 | 152.83 | 0.73 | 424.40 | 7.00 | 0.90 | 162.98 | 0.50 | -0.63 | -1.82 |
| ALP142 | 294.30 | 10.08 | 113.18 | 358.12 | 0.34 | 0.45 | 465.02 | 1.00 | 14.56 | 179.89 | 0.30 | 166.35 | 1.31 | 424.40 | 7.00 | 0.91 | 162.98 | 0.50 | -0.63 | -2.25 |
| ALP143 | 351.15 | 11.34 | 127.21 | 335.85 | 0.39 | 1.36 | 511.47 | 1.64 | 19.78 | 206.49 | 0.28 | 156.09 | 1.24 | 423.89 | 7.07 | 0.85 | 162.35 | 0.50 | -0.59 | -1.93 |
| ALP144 | 375.95 | 11.00 | 127.73 | 347.67 | 0.39 | 1.29 | 537.52 | 0.79 | 10.42 | 203.34 | 0.41 | 171.35 | 0.23 | 463.31 | 7.12 | 0.93 | 173.71 | 0.49 | -0.44 | -2.81 |
| ALP145 | 325.48 | 10.87 | 121.31 | 356.06 | 0.41 | 0.90 | 510.26 | 2.69 | 15.72 | 199.10 | 0.29 | 155.51 | 1.35 | 467.67 | 7.57 | 0.93 | 169.74 | 0.47 | -0.06 | -1.44 |
| ALP146 | 352.02 | 10.22 | 127.39 | 358.94 | 0.44 | 1.08 | 483.57 | 3.02 | 12.07 | 182.92 | 0.36 | 158.86 | 0.59 | 439.32 | 7.18 | 0.91 | 165.63 | 0.49 | -0.45 | -1.46 |
| ALP147 | 345.51 | 11.54 | 121.19 | 282.89 | 0.37 | 1.40 | 531.30 | 1.21 | 11.16 | 201.61 | 0.39 | 105.30 | 0.30 | 439.64 | 7.05 | 0.91 | 167.36 | 0.50 | -0.56 | 0.71 |
| ALP148 | 375.45 | 10.08 | 127.16 | 349.53 | 0.41 | 1.25 | 520.34 | 0.84 | 11.47 | 199.82 | 0.39 | 170.27 | 0.31 | 454.10 | 7.18 | 0.93 | 170.09 | 0.49 | -0.41 | -2.71 |
| ALP149 | 332.59 | 11.78 | 123.31 | 351.63 | 0.39 | 1.24 | 525.28 | 2.79 | 13.07 | 203.73 | 0.38 | 153.20 | 0.40 | 392.60 | 7.14 | 0.93 | 152.09 | 0.51 | -0.63 | -1.42 |
| ALP150 | 337.88 | 11.74 | 125.83 | 320.41 | 0.38 | 1.23 | 455.37 | 2.48 | 11.81 | 166.82 | 0.35 | 146.20 | 0.82 | 389.54 | 7.51 | 0.82 | 145.72 | 0.48 | -0.27 | -1.51 |
| ALP151 | 333.92 | 10.43 | 123.55 | 339.03 | 0.41 | 0.91 | 440.09 | 0.77 | 18.04 | 186.92 | 0.24 | 173.01 | 1.12 | 437.57 | 7.16 | 0.94 | 165.46 | 0.49 | -0.48 | -3.17 |
| ALP152 | 296.83 | 10.22 | 115.95 | 347.45 | 0.38 | 0.74 | 392.87 | 3.22 | 19.82 | 170.59 | 0.16 | 144.02 | 1.64 | 423.13 | 7.94 | 0.89 | 151.14 | 0.43 | 0.16 | -0.76 |
| ALP154 | 347.95 | 11.10 | 125.60 | 369.91 | 0.37 | 1.15 | 523.65 | 6.00 | 12.17 | 192.41 | 0.28 | 151.88 | 1.85 | 454.31 | 7.12 | 0.75 | 171.47 | 0.50 | -0.49 | 0.67 |
| ALP155 | 349.48 | 9.98 | 127.36 | 379.26 | 0.41 | 0.83 | 491.09 | 6.33 | 19.38 | 196.24 | 0.22 | 162.53 | 1.75 | 468.90 | 7.20 | 0.67 | 175.31 | 0.49 | -0.40 | 0.64 |
| ALP156 | 339.74 | 11.48 | 124.83 | 321.78 | 0.39 | 1.43 | 465.23 | 2.93 | 17.39 | 181.86 | 0.23 | 143.91 | 1.87 | 385.42 | 7.10 | 0.76 | 150.31 | 0.51 | -0.67 | -1.11 |
| ALP157 | 278.60 | 8.91 | 100.69 | 344.72 | 0.44 | 0.69 | 487.56 | 2.85 | 18.24 | 207.36 | 0.31 | 150.28 | 0.68 | 553.39 | 7.43 | 0.93 | 201.54 | 0.47 | -0.14 | -1.33 |
| ALP158 | 328.61 | 11.86 | 124.84 | 353.02 | 0.38 | 1.25 | 442.50 | 1.14 | 21.33 | 188.87 | 0.22 | 136.10 | 1.33 | 361.48 | 7.08 | 0.93 | 142.99 | 0.52 | -0.74 | -0.98 |
| ALP159 | 299.10 | 10.13 | 107.41 | 344.67 | 0.37 | 0.67 | 523.37 | 1.05 | 15.41 | 205.09 | 0.30 | 173.13 | 1.13 | 546.28 | 7.15 | 0.95 | 199.61 | 0.48 | -0.24 | -3.00 |
| ALP160 | 315.99 | 11.95 | 120.44 | 309.26 | 0.38 | 1.22 | 500.19 | 3.65 | 13.40 | 194.46 | 0.35 | 125.84 | 0.55 | 377.42 | 7.24 | 0.82 | 146.91 | 0.51 | -0.63 | 0.01 |
| ALP161 | 360.68 | 10.94 | 121.46 | 338.80 | 0.38 | 1.41 | 515.69 | 1.63 | 11.33 | 198.01 | 0.39 | 169.47 | 0.25 | 460.56 | 7.04 | 0.90 | 173.90 | 0.50 | -0.52 | -2.77 |
| ALP162 | 376.52 | 12.10 | 124.38 | 347.74 | 0.36 | 1.32 | 533.55 | 4.21 | 10.39 | 201.60 | 0.43 | 129.55 | 0.09 | 521.38 | 7.11 | 0.84 | 193.07 | 0.49 | -0.36 | 1.04 |
| ALP163 | 348.73 | 11.51 | 121.52 | 352.26 | 0.37 | 1.29 | 525.15 | 2.83 | 9.88 | 196.05 | 0.40 | 154.00 | 0.46 | 449.48 | 7.13 | 0.94 | 170.01 | 0.50 | -0.50 | -1.47 |
| ALP165 | 340.39 | 9.87 | 122.72 | 357.45 | 0.40 | 0.83 | 519.01 | 3.17 | 12.61 | 199.13 | 0.37 | 161.06 | 0.51 | 449.97 | 7.06 | 0.91 | 170.54 | 0.50 | -0.54 | -1.62 |
| ALP166 | 360.15 | 10.94 | 127.68 | 351.87 | 0.41 | 1.32 | 506.45 | 3.21 | 10.71 | 195.06 | 0.39 | 159.61 | 0.27 | 433.20 | 7.05 | 0.90 | 165.03 | 0.50 | -0.57 | -1.61 |
| ALP167 | 321.01 | 11.21 | 120.01 | 328.61 | 0.38 | 1.08 | 420.76 | 2.48 | 16.67 | 175.90 | 0.23 | 137.63 | 1.34 | 391.53 | 7.25 | 0.94 | 149.95 | 0.50 | -0.51 | -0.63 |
| ALP168 | 325.26 | 10.88 | 117.96 | 355.99 | 0.40 | 0.40 | 525.37 | 2.81 | 11.30 | 201.43 | 0.40 | 158.85 | 0.17 | 545.15 | 7.18 | 0.94 | 199.97 | 0.48 | -0.26 | -1.59 |
| ALP169 | 343.83 | 11.39 | 129.59 | 357.36 | 0.41 | 0.96 | 483.68 | 3.04 | 12.66 | 183.20 | 0.35 | 153.08 | 0.66 | 412.40 | 7.41 | 0.93 | 154.33 | 0.48 | -0.32 | -1.12 |
| ALP170 | 333.49 | 12.04 | 126.72 | 347.38 | 0.37 | 1.14 | 430.35 | 1.60 | 20.58 | 185.23 | 0.21 | 155.31 | 1.36 | 396.84 | 7.16 | 0.88 | 152.57 | 0.50 | -0.57 | -1.61 |
| ALP171 | 307.08 | 9.70 | 121.27 | 360.94 | 0.44 | 0.26 | 454.91 | 3.25 | 19.93 | 192.54 | 0.20 | 161.61 | 1.62 | 434.67 | 7.16 | 0.93 | 165.03 | 0.50 | -0.50 | -1.57 |
| ALP172 | 351.43 | 10.45 | 122.89 | 324.60 | 0.40 | 1.32 | 465.05 | 2.36 | 16.07 | 184.59 | 0.27 | 137.36 | 1.36 | 474.35 | 7.05 | 0.95 | 178.20 | 0.49 | -0.48 | -0.88 |
| ALP173 | 355.31 | 9.72 | 125.02 | 339.42 | 0.42 | 1.04 | 469.04 | 2.05 | 18.63 | 196.91 | 0.29 | 130.44 | 0.97 | 463.41 | 7.08 | 0.97 | 174.73 | 0.49 | -0.49 | -0.42 |

**Table S5.** Extracted climatic variable from CHELSA database and their detail (CHELSA V2.1 Technical Specifications, https://chelsa-climate.org).

| **Full name** | **Code** | **Unit** | **Scale** | **Offset** | **Explanation** |
| --- | --- | --- | --- | --- | --- |
| annual precipitation amount | bio12 | kg m^-2^ year^-1^ | 0.1 | 0 | Accumulated precipitation amount over 1 year |
| annual range of air temperature | bio7 | °C | 0.1 | 0 | The difference between the Maximum Temperature of Warmest month and the Minimum Temperature of Coldest month |
| frost change frequency | fcf | count | - | - | Number of events in which tmin or tmax go above, or below 0°C |
| growing season length TREELIM | gsl | number of days | - | - | Length of the growing season |
| mean annual air temperature | bio1 | °C | 0.1 | -273.15 | mean annual daily mean air temperatures averaged over 1 year |
| mean monthly climate moisture index | cmi_mean | kg m^-2^ month^-1^ | 0.1 | 0 | Average monthly climate moisture index over 1 year |
| number of growing degree days | ngd0 | number of days | - | - | Number of days at which tas > 0°C |
| precipitation seasonality | bio15 | kg m^-2^ | 0.1 | 0 | The Coefficient of Variation is the standard deviation of the monthly precipitation estimates expressed as a percentage of the mean of those estimates (i.e. the annual mean) |
| snow cover day | scd | count | - | - | Number of days with snowcover calculated using the snowpack model implementation in from TREELIM (https://doi.org/10.1007/s00035-014 0124-0) |

**Table S6.** The climatic variables including snow cover days, number of growing degree days, growing season length, frost change frequency, mean monthly climate, moisture index, precipitation seasonality, annual precipitation amount; annual range of air temperature and mean annual air temperature as extracted from the CHELSA database.

| **Tree** | **Snow cover days** | **number of growing degree days** | **growing season length TREELIM** | **frost change frequency** | **mean monthly climate moisture index** | **Precipitation seasonality** | **annual precipitation amount** | **annual range of air temperature** | **mean annual air temperature** |
| --- | --- | --- | --- | --- | --- | --- | --- | --- | --- |
| ALP001 | 139 | 269 | 216 | 133 | 13.7 | 27.8 | 1104.3 | 28.1 | 6.65 |
| ALP002 | 139 | 269 | 216 | 133 | 13.7 | 27.8 | 1104.3 | 28.1 | 6.65 |
| ALP003 | 139 | 269 | 216 | 133 | 13.7 | 27.8 | 1104.3 | 28.1 | 6.65 |
| ALP004 | 139 | 269 | 216 | 133 | 13.7 | 27.8 | 1104.3 | 28.1 | 6.65 |
| ALP005 | 139 | 269 | 216 | 133 | 13.7 | 27.8 | 1104.3 | 28.1 | 6.65 |
| ALP006 | 139 | 269 | 216 | 133 | 13.7 | 27.8 | 1104.3 | 28.1 | 6.65 |
| ALP010 | 139 | 269 | 216 | 133 | 13.7 | 27.8 | 1104.3 | 28.1 | 6.65 |
| ALP011 | 139 | 269 | 216 | 133 | 13.7 | 27.8 | 1104.3 | 28.1 | 6.65 |
| ALP012 | 139 | 269 | 216 | 133 | 13.7 | 27.8 | 1104.3 | 28.1 | 6.65 |
| ALP014 | 148 | 261 | 204 | 118 | 25.7 | 27.8 | 1175.2 | 28.1 | 5.95 |
| ALP015 | 148 | 261 | 204 | 118 | 25.7 | 27.8 | 1175.2 | 28.1 | 5.95 |
| ALP016 | 148 | 261 | 204 | 118 | 25.7 | 27.8 | 1175.2 | 28.1 | 5.95 |
| ALP017 | 146 | 262 | 206 | 122 | 21.9 | 28.3 | 1169.5 | 28 | 6.15 |
| ALP019 | 146 | 262 | 206 | 122 | 21.9 | 28.3 | 1169.5 | 28 | 6.15 |
| ALP020 | 146 | 262 | 206 | 122 | 21.9 | 28.3 | 1169.5 | 28 | 6.15 |
| ALP021 | 146 | 262 | 206 | 122 | 21.9 | 28.3 | 1169.5 | 28 | 6.15 |
| ALP022 | 146 | 262 | 206 | 122 | 21.9 | 28.3 | 1169.5 | 28 | 6.15 |
| ALP023 | 146 | 262 | 206 | 122 | 21.9 | 28.3 | 1169.5 | 28 | 6.15 |
| ALP024 | 146 | 262 | 206 | 122 | 21.9 | 28.3 | 1169.5 | 28 | 6.15 |
| ALP025 | 146 | 262 | 206 | 122 | 21.9 | 28.3 | 1169.5 | 28 | 6.15 |
| ALP026 | 146 | 262 | 206 | 122 | 21.9 | 28.3 | 1169.5 | 28 | 6.15 |
| ALP027 | 146 | 262 | 206 | 122 | 21.9 | 28.3 | 1169.5 | 28 | 6.15 |
| ALP028 | 146 | 262 | 206 | 122 | 21.9 | 28.3 | 1169.5 | 28 | 6.15 |
| ALP029 | 146 | 262 | 206 | 122 | 21.9 | 28.3 | 1169.5 | 28 | 6.15 |
| **Tree** | **Snow cover days** | **number of growing degree days** | **growing season length TREELIM** | **frost change frequency** | **mean monthly climate moisture index** | **Precipitation seasonality** | **annual precipitation amount** | **annual range of air temperature** | **mean annual air temperature** |
| ALP030 | 146 | 262 | 206 | 122 | 21.9 | 28.3 | 1169.5 | 28 | 6.15 |
| ALP032 | 158 | 256 | 192 | 114 | 29.1 | 29.5 | 1235.5 | 27.8 | 5.55 |
| ALP033 | 158 | 256 | 192 | 114 | 29.1 | 29.5 | 1235.5 | 27.8 | 5.55 |
| ALP034 | 158 | 256 | 192 | 114 | 29.1 | 29.5 | 1235.5 | 27.8 | 5.55 |
| ALP035 | 158 | 256 | 192 | 114 | 29.1 | 29.5 | 1235.5 | 27.8 | 5.55 |
| ALP036 | 158 | 256 | 192 | 114 | 29.1 | 29.5 | 1235.5 | 27.8 | 5.55 |
| ALP037 | 158 | 256 | 192 | 114 | 29.1 | 29.5 | 1235.5 | 27.8 | 5.55 |
| ALP038 | 158 | 256 | 192 | 114 | 29.1 | 29.5 | 1235.5 | 27.8 | 5.55 |
| ALP039 | 158 | 256 | 192 | 114 | 29.1 | 29.5 | 1235.5 | 27.8 | 5.55 |
| ALP040 | 158 | 256 | 192 | 114 | 29.1 | 29.5 | 1235.5 | 27.8 | 5.55 |
| ALP041 | 158 | 256 | 192 | 114 | 29.1 | 29.5 | 1235.5 | 27.8 | 5.55 |
| ALP042 | 158 | 256 | 192 | 114 | 29.1 | 29.5 | 1235.5 | 27.8 | 5.55 |
| ALP043 | 158 | 256 | 192 | 114 | 29.1 | 29.5 | 1235.5 | 27.8 | 5.55 |
| ALP044 | 158 | 256 | 192 | 114 | 29.1 | 29.5 | 1235.5 | 27.8 | 5.55 |
| ALP045 | 158 | 256 | 192 | 114 | 29.1 | 29.5 | 1235.5 | 27.8 | 5.55 |
| ALP046 | 158 | 256 | 192 | 114 | 29.1 | 29.5 | 1235.5 | 27.8 | 5.55 |
| ALP047 | 185 | 243 | 175 | 107 | 40 | 29.9 | 1341.3 | 27.7 | 4.65 |
| ALP048 | 185 | 243 | 175 | 107 | 40 | 29.9 | 1341.3 | 27.7 | 4.65 |
| ALP050 | 185 | 243 | 175 | 107 | 40 | 29.9 | 1341.3 | 27.7 | 4.65 |
| ALP051 | 185 | 243 | 175 | 107 | 40 | 29.9 | 1341.3 | 27.7 | 4.65 |
| ALP052 | 185 | 243 | 175 | 107 | 40 | 29.9 | 1341.3 | 27.7 | 4.65 |
| ALP053 | 185 | 243 | 175 | 107 | 40 | 29.9 | 1341.3 | 27.7 | 4.65 |
| ALP054 | 185 | 243 | 175 | 107 | 40 | 29.9 | 1341.3 | 27.7 | 4.65 |
| ALP055 | 185 | 243 | 175 | 107 | 40 | 29.9 | 1341.3 | 27.7 | 4.65 |
| ALP056 | 185 | 243 | 175 | 107 | 40 | 29.9 | 1341.3 | 27.7 | 4.65 |
| ALP057 | 185 | 243 | 175 | 107 | 40 | 29.9 | 1341.3 | 27.7 | 4.65 |
| ALP058 | 185 | 243 | 175 | 107 | 40 | 29.9 | 1341.3 | 27.7 | 4.65 |
| **Tree** | **Snow cover days** | **number of growing degree days** | **growing season length TREELIM** | **frost change frequency** | **mean monthly climate moisture index** | **Precipitation seasonality** | **annual precipitation amount** | **annual range of air temperature** | **mean annual air temperature** |
| ALP059 | 185 | 243 | 175 | 107 | 40 | 29.9 | 1341.3 | 27.7 | 4.65 |
| ALP060 | 185 | 243 | 175 | 107 | 40 | 29.9 | 1341.3 | 27.7 | 4.65 |
| ALP061 | 254 | 186 | 107 | 110 | 67.8 | 30.1 | 1627.8 | 27.8 | 0.15 |
| ALP062 | 254 | 186 | 107 | 110 | 67.8 | 30.1 | 1627.8 | 27.8 | 0.15 |
| ALP063 | 254 | 186 | 107 | 110 | 67.8 | 30.1 | 1627.8 | 27.8 | 0.15 |
| ALP064 | 254 | 186 | 107 | 110 | 67.8 | 30.1 | 1627.8 | 27.8 | 0.15 |
| ALP065 | 254 | 186 | 107 | 110 | 67.8 | 30.1 | 1627.8 | 27.8 | 0.15 |
| ALP066 | 253 | 188 | 108 | 109 | 68 | 30.6 | 1697.8 | 27.7 | 0.35 |
| ALP067 | 253 | 188 | 108 | 109 | 68 | 30.6 | 1697.8 | 27.7 | 0.35 |
| ALP068 | 253 | 188 | 108 | 109 | 68 | 30.6 | 1697.8 | 27.7 | 0.35 |
| ALP069 | 253 | 188 | 108 | 109 | 68 | 30.6 | 1697.8 | 27.7 | 0.35 |
| ALP070 | 253 | 188 | 108 | 109 | 68 | 30.6 | 1697.8 | 27.7 | 0.35 |
| ALP071 | 253 | 188 | 108 | 109 | 68 | 30.6 | 1697.8 | 27.7 | 0.35 |
| ALP072 | 253 | 188 | 108 | 109 | 68 | 30.6 | 1697.8 | 27.7 | 0.35 |
| ALP073 | 257 | 185 | 103 | 109 | 76.2 | 31 | 1789.5 | 27.7 | 0.05 |
| ALP074 | 257 | 185 | 103 | 109 | 76.2 | 31 | 1789.5 | 27.7 | 0.05 |
| ALP075 | 257 | 185 | 103 | 109 | 76.2 | 31 | 1789.5 | 27.7 | 0.05 |
| ALP076 | 257 | 185 | 103 | 109 | 76.2 | 31 | 1789.5 | 27.7 | 0.05 |
| ALP077 | 257 | 185 | 103 | 109 | 76.2 | 31 | 1789.5 | 27.7 | 0.05 |
| ALP078 | 237 | 198 | 121 | 107 | 65.5 | 30.7 | 1684.8 | 27.7 | 1.15 |
| ALP079 | 237 | 198 | 121 | 107 | 65.5 | 30.7 | 1684.8 | 27.7 | 1.15 |
| ALP080 | 237 | 198 | 121 | 107 | 65.5 | 30.7 | 1684.8 | 27.7 | 1.15 |
| ALP081 | 237 | 198 | 121 | 107 | 65.5 | 30.7 | 1684.8 | 27.7 | 1.15 |
| ALP082 | 237 | 198 | 121 | 107 | 65.5 | 30.7 | 1684.8 | 27.7 | 1.15 |
| ALP083 | 237 | 198 | 121 | 107 | 65.5 | 30.7 | 1684.8 | 27.7 | 1.15 |
| ALP085 | 190 | 241 | 170 | 107 | 54.6 | 30.4 | 1487.5 | 27.7 | 4.45 |
| ALP086 | 190 | 241 | 170 | 107 | 54.6 | 30.4 | 1487.5 | 27.7 | 4.45 |
| **Tree** | **Snow cover days** | **number of growing degree days** | **growing season length TREELIM** | **frost change frequency** | **mean monthly climate moisture index** | **Precipitation seasonality** | **annual precipitation amount** | **annual range of air temperature** | **mean annual air temperature** |
| ALP087 | 190 | 241 | 170 | 107 | 54.6 | 30.4 | 1487.5 | 27.7 | 4.45 |
| ALP088 | 190 | 241 | 170 | 107 | 54.6 | 30.4 | 1487.5 | 27.7 | 4.45 |
| ALP089 | 190 | 241 | 170 | 107 | 54.6 | 30.4 | 1487.5 | 27.7 | 4.45 |
| ALP090 | 190 | 241 | 170 | 107 | 54.6 | 30.4 | 1487.5 | 27.7 | 4.45 |
| ALP091 | 235 | 200 | 125 | 106 | 89.7 | 30.7 | 1789.3 | 27.7 | 1.35 |
| ALP092 | 235 | 200 | 125 | 106 | 89.7 | 30.7 | 1789.3 | 27.7 | 1.35 |
| ALP093 | 235 | 200 | 125 | 106 | 89.7 | 30.7 | 1789.3 | 27.7 | 1.35 |
| ALP094 | 235 | 200 | 125 | 106 | 89.7 | 30.7 | 1789.3 | 27.7 | 1.35 |
| ALP095 | 235 | 200 | 125 | 106 | 89.7 | 30.7 | 1789.3 | 27.7 | 1.35 |
| ALP096 | 235 | 200 | 125 | 106 | 89.7 | 30.7 | 1789.3 | 27.7 | 1.35 |
| ALP097 | 235 | 200 | 125 | 106 | 89.7 | 30.7 | 1789.3 | 27.7 | 1.35 |
| ALP099 | 231 | 204 | 130 | 106 | 77.7 | 30.7 | 1743.9 | 27.7 | 1.65 |
| ALP100 | 231 | 204 | 130 | 106 | 77.7 | 30.7 | 1743.9 | 27.7 | 1.65 |
| ALP101 | 231 | 204 | 130 | 106 | 77.7 | 30.7 | 1743.9 | 27.7 | 1.65 |
| ALP102 | 231 | 204 | 130 | 106 | 77.7 | 30.7 | 1743.9 | 27.7 | 1.65 |
| ALP103 | 231 | 204 | 130 | 106 | 77.7 | 30.7 | 1743.9 | 27.7 | 1.65 |
| ALP104 | 208 | 225 | 153 | 105 | 62.9 | 30.6 | 1591.0 | 27.6 | 3.25 |
| ALP105 | 208 | 225 | 153 | 105 | 62.9 | 30.6 | 1591.0 | 27.6 | 3.25 |
| ALP106 | 190 | 241 | 170 | 107 | 54.6 | 30.4 | 1487.5 | 27.7 | 4.45 |
| ALP107 | 190 | 241 | 170 | 107 | 54.6 | 30.4 | 1487.5 | 27.7 | 4.45 |
| ALP110 | 267 | 179 | 93 | 111 | 103.9 | 32.4 | 2063.5 | 27.6 | -0.45 |
| ALP111 | 267 | 179 | 93 | 111 | 103.9 | 32.4 | 2063.5 | 27.6 | -0.45 |
| ALP112 | 267 | 179 | 93 | 111 | 103.9 | 32.4 | 2063.5 | 27.6 | -0.45 |
| ALP113 | 267 | 179 | 93 | 111 | 103.9 | 32.4 | 2063.5 | 27.6 | -0.45 |
| ALP114 | 267 | 179 | 93 | 111 | 103.9 | 32.4 | 2063.5 | 27.6 | -0.45 |
| ALP115 | 264 | 181 | 96 | 110 | 108.3 | 32.3 | 2067.4 | 27.7 | -0.15 |
| ALP116 | 264 | 181 | 96 | 110 | 108.3 | 32.3 | 2067.4 | 27.7 | -0.15 |
| **Tree** | **Snow cover days** | **number of growing degree days** | **growing season length TREELIM** | **frost change frequency** | **mean monthly climate moisture index** | **Precipitation seasonality** | **annual precipitation amount** | **annual range of air temperature** | **mean annual air temperature** |
| ALP117 | 264 | 181 | 96 | 110 | 108.3 | 32.3 | 2067.4 | 27.7 | -0.15 |
| ALP118 | 264 | 181 | 96 | 110 | 108.3 | 32.3 | 2067.4 | 27.7 | -0.15 |
| ALP119 | 267 | 179 | 93 | 111 | 103.9 | 32.4 | 2063.5 | 27.6 | -0.45 |
| ALP120 | 267 | 179 | 93 | 111 | 103.9 | 32.4 | 2063.5 | 27.6 | -0.45 |
| ALP121 | 267 | 179 | 93 | 111 | 103.9 | 32.4 | 2063.5 | 27.6 | -0.45 |
| ALP122 | 275 | 172 | 85 | 114 | 102.1 | 32.2 | 2047.3 | 27.7 | -0.95 |
| ALP123 | 275 | 172 | 85 | 114 | 102.1 | 32.2 | 2047.3 | 27.7 | -0.95 |
| ALP124 | 297 | 159 | 62 | 119 | 108.2 | 32.5 | 2118.2 | 27.7 | -2.05 |
| ALP125 | 297 | 159 | 62 | 119 | 108.2 | 32.5 | 2118.2 | 27.7 | -2.05 |
| ALP126 | 297 | 159 | 62 | 119 | 108.2 | 32.5 | 2118.2 | 27.7 | -2.05 |
| ALP127 | 297 | 159 | 62 | 119 | 108.2 | 32.5 | 2118.2 | 27.7 | -2.05 |
| ALP128 | 297 | 159 | 62 | 119 | 108.2 | 32.5 | 2118.2 | 27.7 | -2.05 |
| ALP129 | 275 | 172 | 85 | 114 | 102.1 | 32.2 | 2047.3 | 27.7 | -0.95 |
| ALP130 | 275 | 172 | 85 | 114 | 102.1 | 32.2 | 2047.3 | 27.7 | -0.95 |
| ALP131 | 275 | 172 | 85 | 114 | 102.1 | 32.2 | 2047.3 | 27.7 | -0.95 |
| ALP132 | 275 | 172 | 85 | 114 | 102.1 | 32.2 | 2047.3 | 27.7 | -0.95 |
| ALP134 | 275 | 172 | 85 | 114 | 102.1 | 32.2 | 2047.3 | 27.7 | -0.95 |
| ALP135 | 275 | 172 | 85 | 114 | 102.1 | 32.2 | 2047.3 | 27.7 | -0.95 |
| ALP136 | 275 | 172 | 85 | 114 | 102.1 | 32.2 | 2047.3 | 27.7 | -0.95 |
| ALP139 | 275 | 172 | 85 | 114 | 102.1 | 32.2 | 2047.3 | 27.7 | -0.95 |
| ALP140 | 254 | 187 | 106 | 109 | 72.2 | 30.8 | 1750.4 | 27.7 | 0.25 |
| ALP141 | 254 | 187 | 106 | 109 | 72.2 | 30.8 | 1750.4 | 27.7 | 0.25 |
| ALP142 | 254 | 187 | 106 | 109 | 72.2 | 30.8 | 1750.4 | 27.7 | 0.25 |
| ALP143 | 244 | 190 | 112 | 108 | 57.6 | 30.3 | 1599.0 | 27.8 | 0.55 |
| ALP144 | 271 | 173 | 88 | 112 | 86.4 | 32.9 | 1859.7 | 27.6 | -0.95 |
| ALP145 | 271 | 173 | 88 | 112 | 86.4 | 32.9 | 1859.7 | 27.6 | -0.95 |
| ALP146 | 271 | 173 | 88 | 112 | 86.4 | 32.9 | 1859.7 | 27.6 | -0.95 |
| **Tree** | **Snow cover days** | **number of growing degree days** | **growing season length TREELIM** | **frost change frequency** | **mean monthly climate moisture index** | **Precipitation seasonality** | **annual precipitation amount** | **annual range of air temperature** | **mean annual air temperature** |
| ALP147 | 271 | 173 | 88 | 112 | 86.4 | 32.9 | 1859.7 | 27.6 | -0.95 |
| ALP148 | 271 | 173 | 88 | 112 | 86.4 | 32.9 | 1859.7 | 27.6 | -0.95 |
| ALP149 | 271 | 173 | 88 | 112 | 86.4 | 32.9 | 1859.7 | 27.6 | -0.95 |
| ALP150 | 271 | 173 | 88 | 112 | 86.4 | 32.9 | 1859.7 | 27.6 | -0.95 |
| ALP151 | 271 | 173 | 88 | 112 | 86.4 | 32.9 | 1859.7 | 27.6 | -0.95 |
| ALP152 | 271 | 173 | 88 | 112 | 86.4 | 32.9 | 1859.7 | 27.6 | -0.95 |
| ALP154 | 151 | 255 | 198 | 113 | 16.3 | 33.3 | 1057.6 | 27.8 | 5.55 |
| ALP155 | 151 | 255 | 198 | 113 | 16.3 | 33.3 | 1057.6 | 27.8 | 5.55 |
| ALP156 | 189 | 234 | 172 | 104 | 34.2 | 32.9 | 1150.7 | 27.9 | 3.95 |
| ALP157 | 189 | 234 | 172 | 104 | 34.2 | 32.9 | 1150.7 | 27.9 | 3.95 |
| ALP158 | 173 | 248 | 187 | 108 | 36.9 | 32.9 | 1260.0 | 27.7 | 5.05 |
| ALP159 | 192 | 233 | 168 | 104 | 38.8 | 33.3 | 1304.8 | 27.7 | 3.85 |
| ALP160 | 192 | 233 | 168 | 104 | 38.8 | 33.3 | 1304.8 | 27.7 | 3.85 |
| ALP161 | 192 | 233 | 168 | 104 | 38.8 | 33.3 | 1304.8 | 27.7 | 3.85 |
| ALP162 | 179 | 243 | 181 | 105 | 33.1 | 33.2 | 1228.2 | 27.7 | 4.55 |
| ALP163 | 218 | 212 | 143 | 103 | 58.3 | 32.2 | 1454.8 | 27.6 | 2.35 |
| ALP165 | 218 | 212 | 143 | 103 | 58.3 | 32.2 | 1454.8 | 27.6 | 2.35 |
| ALP166 | 218 | 212 | 143 | 103 | 58.3 | 32.2 | 1454.8 | 27.6 | 2.35 |
| ALP167 | 218 | 212 | 143 | 103 | 58.3 | 32.2 | 1454.8 | 27.6 | 2.35 |
| ALP168 | 218 | 212 | 143 | 103 | 58.3 | 32.2 | 1454.8 | 27.6 | 2.35 |
| ALP169 | 250 | 190 | 111 | 107 | 72.3 | 32.3 | 1696.3 | 27.6 | 0.55 |
| ALP170 | 250 | 190 | 111 | 107 | 72.3 | 32.3 | 1696.3 | 27.6 | 0.55 |
| ALP171 | 250 | 190 | 111 | 107 | 72.3 | 32.3 | 1696.3 | 27.6 | 0.55 |
| ALP172 | 250 | 190 | 111 | 107 | 72.3 | 32.3 | 1696.3 | 27.6 | 0.55 |
| ALP173 | 250 | 190 | 111 | 107 | 72.3 | 32.3 | 1696.3 | 27.6 | 0.55 |

**Table S7.** The soil variables including total phosphorus, available phosphorus, total nitrogen, total carbon, soil pH and conductivity as measured using the combined soil sample for each plant individual.

| **Tree** | **Total P (ppm)** | **Available P (ppm)** | **Total N (%)** | **Total C (%)** | **pH** | **Conductivity (uS)** |
| --- | --- | --- | --- | --- | --- | --- |
| ALP001 | 197 | 7.7 | 0.2 | 4.1 | 7.3 | 226.2 |
| ALP002 | 590 | 8.2 | 0.2 | 3.4 | 7.32 | 230.9 |
| ALP003 | 786 | 10.1 | 0.2 | 2.9 | 7.42 | 247.6 |
| ALP004 | 774 | 12 | 0.3 | 3.6 | 7.2 | 268 |
| ALP005 | 727 | 19.3 | 0.3 | 4.4 | 7.11 | 235.8 |
| ALP006 | 197 | 13.8 | 0.3 | 4.1 | 7.2 | 238.8 |
| ALP010 | 537 | 7.9 | 0.2 | 3 | 7.17 | 216 |
| ALP011 | 219 | 11.4 | 0.2 | 3.3 | 7.08 | 219.8 |
| ALP012 | 208 | 39.6 | 0.3 | 4.4 | 7.07 | 251.9 |
| ALP014 | 282 | 6.7 | 0.2 | 3.3 | 7.24 | 212.2 |
| ALP015 | 470 | 37 | 0.5 | 8.2 | 7.04 | 378 |
| ALP016 | 329 | 12.2 | 0.3 | 5.2 | 7.13 | 262.2 |
| ALP017 | 306 | 36.9 | 0.3 | 4.8 | 7.2 | 245.2 |
| ALP019 | 337 | 11.8 | 0.3 | 6 | 7.09 | 362.1 |
| ALP020 | 201 | 18.5 | 0.4 | 5.9 | 7 | 266.7 |
| ALP021 | 338 | 0.5 | 0.1 | 2.6 | 6.64 | 171.4 |
| ALP022 | 271 | 0.3 | 0.6 | 7.6 | 6.78 | 142.6 |
| ALP023 | 702 | 31.1 | 0.7 | 8.5 | 6.78 | 347.6 |
| ALP024 | 103 | 73 | 0.1 | 2.7 | 7.08 | 351.9 |
| ALP025 | 179 | 0.5 | 0.1 | 2.6 | 7.01 | 139.9 |
| ALP026 | 609 | 0.4 | 1.5 | 23.9 | 6.96 | 151.1 |
| ALP027 | 802 | 62.8 | 0.2 | 4.8 | 7 | 531.4 |
| ALP028 | 449 | 14.1 | 1.3 | 18.6 | 6.98 | 243.7 |
| ALP029 | 475 | 49.1 | 0.5 | 7.8 | 7.23 | 379.4 |
| ALP030 | 454 | 14.1 | 1 | 12.8 | 7.58 | 337.7 |
| ALP032 | 479 | 19.6 | 0.2 | 4.3 | 7.3 | 312.7 |
| **Tree** | **Total P (ppm)** | **Available P (ppm)** | **Total N (%)** | **Total C (%)** | **pH** | **Conductivity (uS)** |
| ALP033 | 490 | 6.4 | 0.3 | 6.3 | 7.48 | 220.8 |
| ALP034 | 315 | 14.3 | 0.5 | 8 | 7.31 | 384 |
| ALP035 | 1027 | 0 | 0.1 | 2.8 | 7.47 | 124 |
| ALP036 | 1008 | 0.8 | 0.1 | 3.3 | 7.39 | 161.7 |
| ALP037 | 808 | 2.3 | 0.1 | 2.7 | 7.44 | 190.9 |
| ALP038 | 787 | 36 | 2.2 | 33.5 | 7.18 | 590.2 |
| ALP039 | 670 | 0.9 | 0.1 | 3.6 | 7.27 | 164.4 |
| ALP040 | 635 | 32.8 | 2.4 | 37.4 | 6.93 | 494.1 |
| ALP041 | 770 | 44.3 | 2 | 28.1 | 6.75 | 440.1 |
| ALP042 | 439 | 20.1 | 1.4 | 26.6 | 6.75 | 347 |
| ALP043 | 538 | 62.1 | 1.4 | 26.6 | 6.74 | 410.7 |
| ALP044 | 856 | 75.4 | 1.5 | 24.2 | 7.14 | 483.8 |
| ALP045 | 364 | 4.3 | 0.2 | 4.2 | 7.28 | 225.2 |
| ALP046 | 649 | 0.4 | 0.1 | 2.8 | 7.32 | 146.7 |
| ALP047 | 339 | 16.1 | 0.3 | 5.2 | 7.14 | 254.8 |
| ALP048 | 393 | 19.4 | 0.2 | 4.7 | 7.16 | 267.4 |
| ALP050 | 566 | 39.7 | 0.8 | 12.8 | 7.28 | 408.9 |
| ALP051 | 350 | 99 | 1.7 | 26.1 | 7.21 | 465.9 |
| ALP052 | 283 | 0.4 | 0.1 | 2.5 | 7.43 | 130.7 |
| ALP053 | 474 | 66.7 | 1 | 14.7 | 7.15 | 398.6 |
| ALP054 | 554 | 45.8 | 0.5 | 9.9 | 7.42 | 414.7 |
| ALP055 | 642 | 36 | 1 | 17.1 | 7.25 | 359.3 |
| ALP056 | 208 | 7.7 | 0.2 | 4.9 | 7.29 | 227.6 |
| ALP057 | 620 | 46.3 | 0.9 | 13.6 | 7.17 | 501.9 |
| ALP058 | 923 | 73.9 | 0.8 | 14.6 | 7.13 | 332.1 |
| ALP059 | 138 | 138.4 | 1 | 16.1 | 7.2 | 476.5 |
| ALP060 | 227 | 37.2 | 0.7 | 10.4 | 7.38 | 503.3 |
| ALP061 | 720 | 56.9 | 1.4 | 21.3 | 6.89 | 389.8 |
| ALP062 | 35 | 110.9 | 1.2 | 20.5 | 6.19 | 360.7 |
| **Tree** | **Total P (ppm)** | **Available P (ppm)** | **Total N (%)** | **Total C (%)** | **pH** | **Conductivity (uS)** |
| ALP063 | 317 | 0.1 | 0 | 1.9 | 7 | 101.9 |
| ALP064 | 476 | 37.1 | 1 | 15.8 | 6.58 | 235 |
| ALP065 | 424 | 10.7 | 0.3 | 5.5 | 6.75 | 237.7 |
| ALP066 | 918 | 2.4 | 0.9 | 13.5 | 6.83 | 241 |
| ALP067 | 546 | 16.8 | 0.6 | 9.4 | 7.26 | 396.6 |
| ALP068 | 1036 | 30.9 | 0.9 | 12.5 | 6.78 | 354.7 |
| ALP069 | 265 | 20.5 | 0.8 | 14.8 | 6.99 | 531.4 |
| ALP070 | 440 | 12.8 | 0.5 | 6.9 | 6.7 | 294.1 |
| ALP071 | 148 | 61.8 | 0.6 | 11.1 | 7.29 | 564.1 |
| ALP072 | 731 | 4 | 0.2 | 5.1 | 7.17 | 214.9 |
| ALP073 | 870 | 28.9 | 0.4 | 6 | 6.13 | 134.4 |
| ALP074 | 855 | 49.7 | 1 | 13.3 | 6.97 | 471.3 |
| ALP075 | 135 | 27.7 | 0.8 | 13.3 | 5.75 | 183 |
| ALP076 | 283 | 13 | 0.5 | 7 | 7.1 | 326.3 |
| ALP077 | 131 | 64.4 | 0.9 | 13 | 6.59 | 332.8 |
| ALP078 | 182 | 29.7 | 0.5 | 8.2 | 7.04 | 378.8 |
| ALP079 | 484 | 19.1 | 0.3 | 5 | 7.07 | 268.8 |
| ALP080 | 133 | 23.9 | 0.4 | 6.4 | 7.31 | 324.3 |
| ALP081 | 187 | 45.6 | 0.5 | 7.1 | 7.35 | 401.7 |
| ALP082 | 230 | 33.7 | 0.5 | 6.4 | 7.45 | 344.1 |
| ALP083 | 222 | 35.5 | 0.3 | 3.8 | 7.32 | 239 |
| ALP085 | 239 | 5 | 0.6 | 9.5 | 7.6 | 475.8 |
| ALP086 | 106 | 10.6 | 0.2 | 5.4 | 7.52 | 257.2 |
| ALP087 | 589 | 2.6 | 0.1 | 5.2 | 7.22 | 207.1 |
| ALP088 | 513 | 23 | 0.4 | 10.8 | 7.58 | 379.4 |
| ALP089 | 294 | 8.2 | 0.2 | 6.8 | 7.4 | 238.7 |
| ALP090 | 191 | 16.6 | 0.2 | 7.5 | 7.29 | 289.6 |
| ALP091 | 308 | 12.4 | 0.4 | 7.5 | 7.36 | 329.3 |
| ALP092 | 308 | 12.4 | 0.4 | 7.5 | 7.36 | 329.3 |
| **Tree** | **Total P (ppm)** | **Available P (ppm)** | **Total N (%)** | **Total C (%)** | **pH** | **Conductivity (uS)** |
| ALP093 | 221 | 9.2 | 0.3 | 3.7 | 7.35 | 296.4 |
| ALP094 | 221 | 9.2 | 0.3 | 3.7 | 7.35 | 296.4 |
| ALP095 | 333 | 2.9 | 0.2 | 3.9 | 7.42 | 214.7 |
| ALP096 | 148 | 3 | 0.2 | 2.4 | 7.35 | 173.9 |
| ALP097 | 378 | 16.2 | 0.4 | 8.2 | 7.29 | 459.4 |
| ALP099 | 285 | 16.2 | 0.3 | 3.7 | 7.29 | 335.3 |
| ALP100 | 329 | 18.6 | 0.4 | 6.4 | 7.55 | 397.2 |
| ALP101 | 502 | 12.3 | 0.5 | 5.8 | 7.48 | 367.8 |
| ALP102 | 329 | 22.7 | 0.8 | 9.1 | 7.36 | 546.2 |
| ALP103 | 252 | 78.9 | 1.9 | 29.5 | 7.25 | 482.8 |
| ALP104 | 176 | 4.9 | 0.3 | 6.8 | 7.43 | 279 |
| ALP105 | 71 | 13.1 | 0.3 | 12.1 | 7.35 | 331.5 |
| ALP106 | 487 | 11.9 | 0.5 | 11.7 | 7.64 | 398.9 |
| ALP107 | 203 | 0.8 | 0.1 | 6.6 | 7.71 | 236.3 |
| ALP110 | 460 | 23.6 | 0.5 | 7.9 | 6.22 | 445.2 |
| ALP111 | 499 | 21 | 0.6 | 7.9 | 7.03 | 306.6 |
| ALP112 | 431 | 24.2 | 0.6 | 8 | 6.94 | 330.3 |
| ALP113 | 560 | 37.7 | 0.9 | 12.3 | 5.92 | 304.6 |
| ALP114 | 832 | 18.4 | 0.5 | 6.1 | 6.52 | 227.6 |
| ALP115 | 163 | 19.4 | 0.1 | 1.7 | 7.09 | 195 |
| ALP116 | 273 | 2.3 | 0.1 | 1.8 | 7.23 | 129.2 |
| ALP117 | 118 | 2.6 | 0.1 | 1.5 | 7.34 | 141.6 |
| ALP118 | 369 | 4.9 | 0.2 | 3.4 | 7.3 | 200.1 |
| ALP119 | 377 | 0.7 | 0 | 6.5 | 7.54 | 200.5 |
| ALP120 | 392 | 2.7 | 0.1 | 6.2 | 7.46 | 258.7 |
| ALP121 | 185 | 28.6 | 0.5 | 8.8 | 6.65 | 357.1 |
| ALP122 | 312 | 33.5 | 0.8 | 15.3 | 7.18 | 314.2 |
| ALP123 | 289 | 17.3 | 0.5 | 8.3 | 7.59 | 385.6 |
| ALP124 | 316 | 23.8 | 0.6 | 10.3 | 7.5 | 361.5 |
| **Tree** | **Total P (ppm)** | **Available P (ppm)** | **Total N (%)** | **Total C (%)** | **pH** | **Conductivity (uS)** |
| ALP125 | 251 | 7.8 | 0.3 | 6.9 | 7.64 | 294 |
| ALP126 | 376 | 0.6 | 0.1 | 7 | 7.56 | 239.2 |
| ALP127 | 655 | 12.5 | 0.4 | 8.3 | 7.38 | 505.2 |
| ALP128 | 413 | 0.8 | 0.2 | 7.9 | 7.5 | 245.9 |
| ALP129 | 679 | 22.4 | 0.4 | 7.2 | 7.41 | 290.9 |
| ALP130 | 127 | 12.9 | 0.4 | 6.9 | 7.5 | 393.9 |
| ALP131 | 199 | 26.8 | 0.5 | 10.6 | 7.52 | 446.3 |
| ALP132 | 311 | 11 | 0.3 | 4.5 | 7.35 | 284.1 |
| ALP134 | 180 | 15.7 | 0.4 | 7 | 7.26 | 351.2 |
| ALP135 | 617 | 18.1 | 0.4 | 6.6 | 7.04 | 303.1 |
| ALP136 | 418 | 5.4 | 0.3 | 4.3 | 7.21 | 219.1 |
| ALP139 | 260 | 20.8 | 0.3 | 8.1 | 7.31 | 349.3 |
| ALP140 | 254 | 7.4 | 0.3 | 11.2 | 7.54 | 345.7 |
| ALP141 | 80 | 5.4 | 0.3 | 10.7 | 7.34 | 256.2 |
| ALP142 | 343 | 5.8 | 0.4 | 12.1 | 7.38 | 343.6 |
| ALP143 | 280 | 12.7 | 0.2 | 6.9 | 7.57 | 304.3 |
| ALP144 | 311 | 10.8 | 0.2 | 8.6 | 7.44 | 280 |
| ALP145 | 399 | 2.8 | 0.2 | 8.8 | 7.32 | 317.2 |
| ALP146 | 256 | 5.6 | 0.4 | 11.3 | 7.31 | 372.5 |
| ALP147 | 381 | 0.7 | 0.2 | 9.2 | 7.13 | 206.2 |
| ALP148 | 217 | 0.9 | 0.2 | 9.8 | 7.13 | 248.9 |
| ALP149 | 220 | 0.4 | 0.1 | 9.9 | 7.26 | 188.1 |
| ALP150 | 452 | 1.7 | 0.3 | 11.1 | 7.27 | 285.8 |
| ALP151 | 140 | 1.5 | 0.7 | 11.9 | 7.11 | 274.8 |
| ALP152 | 414 | 0.8 | 0.4 | 9.4 | 7.36 | 209 |
| ALP154 | 337 | 29.5 | 0.6 | 8.6 | 6.32 | 177.9 |
| ALP155 | 499 | 54.4 | 0.5 | 3.4 | 6.93 | 145.5 |
| ALP156 | 504 | 31.7 | 1.1 | 15.4 | 7.16 | 517.5 |
| ALP157 | 773 | 5.2 | 0.4 | 4.8 | 7.13 | 216 |
| **Tree** | **Total P (ppm)** | **Available P (ppm)** | **Total N (%)** | **Total C (%)** | **pH** | **Conductivity (uS)** |
| ALP158 | 842 | 33.3 | 0.7 | 11.2 | 7.56 | 585.8 |
| ALP159 | 732 | 193.1 | 0.4 | 6.5 | 7.45 | 262.3 |
| ALP160 | 447 | 63.9 | 0.5 | 8.4 | 7.16 | 303.1 |
| ALP161 | 164 | 5.2 | 0.2 | 4.4 | 7.41 | 219.4 |
| ALP162 | 407 | 28.5 | 0.5 | 7.9 | 7.3 | 313.4 |
| ALP163 | 126 | 0.7 | 0 | 5.3 | 7.06 | 203.7 |
| ALP165 | 170 | 1.7 | 0.1 | 3.4 | 7.31 | 189.3 |
| ALP166 | 153 | 0.2 | 0 | 2.6 | 7.43 | 135.6 |
| ALP167 | 130 | 27.9 | 0.4 | 7.4 | 7.06 | 400.2 |
| ALP168 | 60 | 3.7 | 0.1 | 7.8 | 7.05 | 227.5 |
| ALP169 | 433 | 23.8 | 0.3 | 5 | 7.33 | 275.6 |
| ALP170 | 259 | 5.2 | 0.1 | 3.2 | 7.25 | 247.4 |
| ALP171 | 317 | 2.3 | 0.1 | 3.6 | 7.43 | 289.1 |
| ALP172 | 172 | 18.3 | 0.3 | 6 | 7.51 | 552.1 |
| ALP173 | 326 | 62 | 0.8 | 11.3 | 7.44 | 574 |

**Table S8.** Studied plant individuals, their identification based on molecular data, the region and elevation they were sampled at, their score on the first axis of PCAs separating them based on climate and soil data, and the percentage of herbivory they have suffered.

| **Tree individuals** | **Molecular ID** | **Valley** | **Elevation** | **Climate PCA1** | **Soil PCA1** | **Herbivory (%)** |
| --- | --- | --- | --- | --- | --- | --- |
| ALP001 | *S. caprea* | Mölltal | 971.16 | 1.8937 | -0.9061 | 0.9409 |
| ALP002 | *S. caprea* | Mölltal | 970.47 | 1.8937 | -0.7406 | 1.3569 |
| ALP003 | *S. caprea* | Mölltal | 970.47 | 1.8937 | -0.6536 | 1.3739 |
| ALP004 | *S. myrsinifolia* | Mölltal | 970.47 | 1.8937 | -0.404 | 0.7657 |
| ALP005 | *S. caprea* | Mölltal | 970.47 | 1.8937 | -0.359 | 1.5098 |
| ALP006 | *S. caprea* | Mölltal | 970.47 | 1.8937 | -0.7108 | 0.7212 |
| ALP010 | *S. myrsinifolia* | Mölltal | 967.34 | 1.8937 | -0.765 | 0.9670 |
| ALP011 | *S. myrsinifolia* | Mölltal | 967.34 | 1.8937 | -0.8333 | 1.5782 |
| ALP012 | *S. myrsinifolia* | Mölltal | 969.69 | 1.8937 | -0.3846 | 2.3592 |
| ALP014 | *S. appendiculata* | Mölltal | 973.13 | 1.5342 | -0.9209 | 2.3689 |
| ALP015 | *S. appendiculata* | Mölltal | 976.48 | 1.5342 | 0.3495 | 3.6294 |
| ALP016 | *S. appendiculata* | Mölltal | 973.13 | 1.5342 | -0.5276 | 2.8036 |
| ALP017 | *S. caprea* | Mölltal | 970.84 | 1.5051 | -0.4041 | 1.1849 |
| ALP019 | *S. caprea* | Mölltal | 973.20 | 1.5051 | -0.2543 | 0.6444 |
| ALP020 | *S. caprea* | Mölltal | 973.20 | 1.5051 | -0.3649 | 2.2675 |
| ALP021 | *S. myrsinifolia* | Mölltal | 973.20 | 1.5051 | -0.9214 | 1.9092 |
| ALP022 | *S. myrsinifolia* | Mölltal | 973.20 | 1.5051 | -0.4419 | 6.4510 |
| ALP023 | *S. appendiculata* | Mölltal | 973.20 | 1.5051 | 0.6107 | 5.3351 |
| ALP024 | *S. myrsinifolia* | Mölltal | 973.20 | 1.5051 | -0.1636 | 3.3844 |
| ALP025 | *S. myrsinifolia* | Mölltal | 973.20 | 1.5051 | -1.2082 | 3.0557 |
| ALP026 | *S. appendiculata* | Mölltal | 973.20 | 1.5051 | 1.1736 | 4.5293 |
| ALP027 | *S. appendiculata* | Mölltal | 975.75 | 1.5051 | 0.6928 | 4.9085 |
| ALP028 | *S. myrsinifolia* | Mölltal | 973.20 | 1.5051 | 0.994 | 1.6446 |
| ALP029 | *S. caprea* | Mölltal | 1032.71 | 1.5051 | 0.3702 | 0.8636 |
| ALP030 | *S. caprea* | Mölltal | 975.75 | 1.5051 | 0.4572 | 0.4088 |
| ALP032 | *S. appendiculata* | Mölltal | 1037.43 | 0.9973 | -0.4635 | 3.0990 |
| ALP033 | *S. appendiculata* | Mölltal | 1037.43 | 0.9973 | -0.6675 | 2.5061 |
| **Tree individuals** | **Molecular ID** | **Region** | **Elevation** | **Climate PCA1** | **Soil PCA1** | **Herbivory (%)** |
| ALP034 | *S. myrsinifolia* | Mölltal | 1037.43 | 0.9973 | -0.0264 | 2.6588 |
| ALP035 | *S. appendiculata* | Mölltal | 1037.43 | 0.9973 | -0.9941 | 1.6428 |
| ALP036 | *S. appendiculata* | Mölltal | 1037.43 | 0.9973 | -0.8589 | 1.6643 |
| ALP037 | *S. myrsinifolia* | Mölltal | 1037.43 | 0.9973 | -0.9291 | 2.0234 |
| ALP038 | *S. appendiculata* | Mölltal | 1037.43 | 0.9973 | 3.4623 | 3.0797 |
| ALP039 | *S. myrsinifolia* | Mölltal | 1037.43 | 0.9973 | -0.9581 | 4.3248 |
| ALP040 | *S. appendiculata* | Mölltal | 1039.74 | 0.9973 | 3.5908 | 4.9688 |
| ALP041 | *S. appendiculata* | Mölltal | 1039.74 | 0.9973 | 2.9409 | 3.1215 |
| ALP042 | *S. appendiculata* | Mölltal | 1039.74 | 0.9973 | 1.8293 | 3.8617 |
| ALP043 | *S. appendiculata* | Mölltal | 1039.74 | 0.9973 | 2.3921 | 3.2209 |
| ALP044 | *S. appendiculata* | Mölltal | 1039.74 | 0.9973 | 2.6309 | 4.2175 |
| ALP045 | *S. myrsinifolia* | Mölltal | 1039.74 | 0.9973 | -0.8437 | 7.0565 |
| ALP046 | *S. myrsinifolia* | Mölltal | 1039.74 | 0.9973 | -1.0703 | 0.9406 |
| ALP047 | *S. appendiculata* | Mölltal | 1158.25 | 0.5134 | -0.5082 | 4.7235 |
| ALP048 | *S. myrsinifolia* | Mölltal | 1158.25 | 0.5134 | -0.5336 | 5.4972 |
| ALP050 | *S. appendiculata* | Mölltal | 1158.25 | 0.5134 | 0.855 | 0.8103 |
| ALP051 | *S. appendiculata* | Mölltal | 1158.25 | 0.5134 | 2.7721 | 3.8981 |
| ALP052 | *S. myrsinifolia* | Mölltal | 1158.25 | 0.5134 | -1.3417 | 2.5387 |
| ALP053 | *S. mielichhoferi* | Mölltal | 1158.25 | 0.5134 | 1.3221 | 2.3100 |
| ALP054 | *S. appendiculata* | Mölltal | 1158.25 | 0.5134 | 0.4898 | 4.5966 |
| ALP055 | *S. appendiculata* | Mölltal | 1158.25 | 0.5134 | 1.1295 | 5.8318 |
| ALP056 | *S. myrsinifolia* | Mölltal | 1158.25 | 0.5134 | -0.854 | 1.6544 |
| ALP057 | *S. caprea* | Mölltal | 1158.25 | 0.5134 | 1.3004 | 4.6084 |
| ALP058 | *S. caprea* | Mölltal | 1162.07 | 0.5134 | 1.3118 | 5.3135 |
| ALP059 | *S. caprea* | Mölltal | 1162.07 | 0.5134 | 2.0115 | 3.3696 |
| ALP060 | *S. caprea* | Mölltal | 1162.07 | 0.5134 | 0.6385 | 6.5241 |
| ALP061 | *S. mielichhoferi* | Mölltal | 1637.14 | -0.4375 | 2.0692 | 1.1684 |
| ALP062 | *S. mielichhoferi* | Mölltal | 1636.52 | -0.4375 | 2.2184 | 0.8905 |
| ALP063 | *S. myrsinifolia* | Mölltal | 1661.30 | -0.4375 | -1.334 | 5.4380 |
| ALP064 | *S. mielichhoferi* | Mölltal | 1662.29 | -0.4375 | 0.9748 | 2.8062 |
| **Tree individuals** | **Molecular ID** | **Region** | **Elevation** | **Climate PCA1** | **Soil PCA1** | **Herbivory (%)** |
| ALP065 | *S. mielichhoferi* | Mölltal | 1662.29 | -0.4375 | -0.389 | 3.4605 |
| ALP066 | *S. mielichhoferi* | Mölltal | 1764.73 | -0.5681 | 0.6133 | 5.4776 |
| ALP067 | *S. appendiculata* | Mölltal | 1765.74 | -0.5681 | 0.3017 | 6.5883 |
| ALP068 | *S. appendiculata* | Mölltal | 1764.58 | -0.5681 | 1.1404 | 4.8166 |
| ALP069 | *S. appendiculata* | Mölltal | 1763.04 | -0.5681 | 1.0138 | 10.1196 |
| ALP070 | *S. appendiculata* | Mölltal | 1765.38 | -0.5681 | 0.0014 | 5.4045 |
| ALP071 | *S. appendiculata* | Mölltal | 1764.70 | -0.5681 | 0.9425 | 5.4266 |
| ALP072 | *S. mielichhoferi* | Mölltal | 1764.70 | -0.5681 | -0.6018 | 2.6927 |
| ALP073 | *S. mielichhoferi* | Mölltal | 1822.19 | -0.7194 | 0.1013 | 5.8030 |
| ALP074 | *S. mielichhoferi* | Mölltal | 1822.81 | -0.7194 | 1.5161 | 4.6631 |
| ALP075 | *S. mielichhoferi* | Mölltal | 1821.30 | -0.7194 | 0.6473 | 6.1666 |
| ALP076 | *S. mielichhoferi* | Mölltal | 1821.61 | -0.7194 | -0.15 | 4.2620 |
| ALP077 | *S. mielichhoferi* | Mölltal | 1821.61 | -0.7194 | 1.0403 | 3.9137 |
| ALP078 | *S. appendiculata* | Mölltal | 1756.60 | -0.4233 | 0.145 | 6.1987 |
| ALP079 | *S. appendiculata* | Mölltal | 1752.79 | -0.4233 | -0.3633 | 5.8317 |
| ALP080 | *S. appendiculata* | Mölltal | 1752.79 | -0.4233 | -0.3172 | 5.1959 |
| ALP081 | *S. mielichhoferi* | Mölltal | 1752.79 | -0.4233 | 0.166 | 2.2861 |
| ALP082 | *S. appendiculata* | Mölltal | 1752.79 | -0.4233 | -0.1164 | 2.3301 |
| ALP083 | *S. appendiculata* | Mölltal | 1752.79 | -0.4233 | -0.5665 | 2.9330 |
| ALP085 | *S. mielichhoferi* | Mölltal | 1237.55 | 0.3112 | 0.0956 | 1.3224 |
| ALP086 | *S. mielichhoferi* | Mölltal | 1237.55 | 0.3112 | -0.876 | 1.6459 |
| ALP087 | *S. mielichhoferi* | Mölltal | 1237.55 | 0.3112 | -0.7911 | 1.7506 |
| ALP088 | *S. mielichhoferi* | Mölltal | 1237.55 | 0.3112 | 0.0993 | 2.6683 |
| ALP089 | *S. mielichhoferi* | Mölltal | 1237.55 | 0.3112 | -0.7299 | 1.4470 |
| ALP090 | *S. mielichhoferi* | Mölltal | 1237.55 | 0.3112 | -0.5188 | 1.3340 |
| ALP091 | *S. mielichhoferi* | Mölltal | 1623.56 | -0.5471 | -0.2859 | 3.1169 |
| ALP092 | *S. mielichhoferi* | Mölltal | 1620.73 | -0.5471 | -0.2859 | 5.1615 |
| ALP093 | *S. appendiculata* | Mölltal | 1621.36 | -0.5471 | -0.6906 | 5.6416 |
| ALP094 | *S. mielichhoferi* | Mölltal | 1588.45 | -0.5471 | -0.6906 | 2.8033 |
| ALP095 | *S. appendiculata* | Mölltal | 1591.90 | -0.5471 | -0.9621 | 9.2890 |
| **Tree individuals** | **Molecular ID** | **Region** | **Elevation** | **Climate PCA1** | **Soil PCA1** | **Herbivory (%)** |
| ALP096 | *S. mielichhoferi* | Mölltal | 1626.09 | -0.5471 | -1.1895 | 4.8897 |
| ALP097 | *S. appendiculata* | Mölltal | 1623.74 | -0.5471 | 0.1273 | 9.5597 |
| ALP099 | *S. mielichhoferi* | Mölltal | 1605.92 | -0.422 | -0.4897 | 7.8553 |
| ALP100 | *S. mielichhoferi* | Mölltal | 1605.92 | -0.422 | -0.1993 | 3.7116 |
| ALP101 | *S. appendiculata* | Mölltal | 1605.92 | -0.422 | -0.1613 | 7.8756 |
| ALP102 | *S. mielichhoferi* | Mölltal | 1605.92 | -0.422 | 0.673 | 1.8429 |
| ALP103 | *S. mielichhoferi* | Mölltal | 1595.74 | -0.422 | 2.8897 | 3.7965 |
| ALP104 | *S. appendiculata* | Mölltal | 1436.18 | -0.0952 | -0.6644 | 6.8524 |
| ALP105 | *S. mielichhoferi* | Mölltal | 1436.18 | -0.0952 | -0.2346 | 4.3804 |
| ALP106 | *S. appendiculata* | Mölltal | 1396.91 | 0.3112 | 0.1292 | 2.2354 |
| ALP107 | *S. mielichhoferi* | Mölltal | 1364.06 | 0.3112 | -1.0486 | 5.7627 |
| ALP110 | *S. mielichhoferi* | Mölltal | 2008.26 | -1.2084 | 0.6677 | 2.7975 |
| ALP111 | *S. mielichhoferi* | Mölltal | 2008.26 | -1.2084 | 0.1315 | 3.5268 |
| ALP112 | *S. mielichhoferi* | Mölltal | 2008.26 | -1.2084 | 0.217 | 4.3959 |
| ALP113 | *S. mielichhoferi* | Mölltal | 2005.39 | -1.2084 | 1.1721 | 2.5577 |
| ALP114 | *S. mielichhoferi* | Mölltal | 2005.39 | -1.2084 | 0.1269 | 4.8899 |
| ALP115 | *S. mielichhoferi* | Mölltal | 1972.38 | -1.1151 | -1.0039 | 4.1208 |
| ALP116 | *S. mielichhoferi* | Mölltal | 1951.78 | -1.1151 | -1.2924 | 9.0135 |
| ALP117 | *S. mielichhoferi* | Mölltal | 1951.78 | -1.1151 | -1.3955 | 5.0583 |
| ALP118 | *S. mielichhoferi* | Mölltal | 1954.87 | -1.1151 | -0.9381 | 4.6536 |
| ALP119 | *S. appendiculata* | Mölltal | 1988.04 | -1.2084 | -1.0589 | 8.1328 |
| ALP120 | *S. appendiculata* | Mölltal | 1988.04 | -1.2084 | -0.8154 | 13.1700 |
| ALP121 | *S. appendiculata* | Mölltal | 2015.50 | -1.2084 | 0.2667 | 2.8822 |
| ALP122 | *S. appendiculata* | Mölltal | 2070.45 | -1.1646 | 0.6311 | 8.8233 |
| ALP123 | *S. mielichhoferi* | Mölltal | 2082.58 | -1.1646 | -0.1 | 6.9658 |
| ALP124 | *S. mielichhoferi* | Mölltal | 2208.59 | -1.4157 | 0.128 | 4.0234 |
| ALP125 | *S. mielichhoferi* | Mölltal | 2208.59 | -1.4157 | -0.6435 | 9.2015 |
| ALP126 | *S. mielichhoferi* | Mölltal | 2208.59 | -1.4157 | -0.8823 | 4.7045 |
| ALP127 | *S. mielichhoferi* | Mölltal | 2208.59 | -1.4157 | 0.3018 | 4.8585 |
| ALP128 | *S. mielichhoferi* | Mölltal | 2162.81 | -1.4157 | -0.7041 | 3.3085 |
| **Tree individuals** | **Molecular ID** | **Region** | **Elevation** | **Climate PCA1** | **Soil PCA1** | **Herbivory (%)** |
| ALP129 | *S. mielichhoferi* | Mölltal | 2108.98 | -1.1646 | -0.1326 | 5.2819 |
| ALP130 | *S. appendiculata* | Mölltal | 2108.67 | -1.1646 | -0.3124 | 6.9562 |
| ALP131 | *S. appendiculata* | Mölltal | 2109.08 | -1.1646 | 0.2132 | 11.6860 |
| ALP132 | *S. mielichhoferi* | Mölltal | 2109.08 | -1.1646 | -0.6174 | 3.3656 |
| ALP134 | *S. mielichhoferi* | Mölltal | 2107.10 | -1.1646 | -0.2591 | 5.6647 |
| ALP135 | *S. appendiculata* | Mölltal | 2107.85 | -1.1646 | -0.0646 | 3.5501 |
| ALP136 | *S. mielichhoferi* | Mölltal | 2108.05 | -1.1646 | -0.7131 | 4.0848 |
| ALP139 | *S. appendiculata* | Mölltal | 2108.52 | -1.1646 | -0.219 | 15.5013 |
| ALP140 | *S. mielichhoferi* | Mölltal | 1838.38 | -0.6384 | -0.2807 | 4.3429 |
| ALP141 | *S. caprea* | Mölltal | 1838.38 | -0.6384 | -0.5286 | 5.3032 |
| ALP142 | *S. caprea* | Mölltal | 1838.38 | -0.6384 | -0.0741 | 3.5026 |
| ALP143 | *S. mielichhoferi* | Mölltal | 1891.98 | -0.3407 | -0.6132 | 3.4922 |
| ALP144 | *S. mielichhoferi* | Ködnitz | 1958.42 | -1.1476 | -0.5339 | 2.6317 |
| ALP145 | *S. mielichhoferi* | Ködnitz | 1958.42 | -1.1476 | -0.4248 | 2.3356 |
| ALP146 | *S. mielichhoferi* | Ködnitz | 1959.97 | -1.1476 | -0.0691 | 2.1357 |
| ALP147 | *S. mielichhoferi* | Ködnitz | 1962.53 | -1.1476 | -0.6027 | 3.1647 |
| ALP148 | *S. mielichhoferi* | Ködnitz | 1962.13 | -1.1476 | -0.5585 | 1.3099 |
| ALP149 | *S. mielichhoferi* | Ködnitz | 1966.48 | -1.1476 | -0.8144 | 0.9074 |
| ALP150 | *S. mielichhoferi* | Ködnitz | 1966.48 | -1.1476 | -0.2673 | 1.8902 |
| ALP151 | *S. mielichhoferi* | Ködnitz | 1966.48 | -1.1476 | -0.0416 | 3.6606 |
| ALP152 | *S. mielichhoferi* | Ködnitz | 1967.13 | -1.1476 | -0.5041 | 2.7169 |
| ALP154 | *S. myrsinifolia* | Ködnitz | 1064.58 | 0.8776 | 0.1494 | 3.3530 |
| ALP155 | *S. myrsinifolia* | Ködnitz | 1064.58 | 0.8776 | -0.1874 | 4.2390 |
| ALP156 | *S. myrsinifolia* | Ködnitz | 1063.47 | 0.4424 | 1.3941 | 1.4391 |
| ALP157 | *S. myrsinifolia* | Ködnitz | 1063.47 | 0.4424 | -0.4157 | 3.6101 |
| ALP158 | *S. myrsinifolia* | Ködnitz | 1176.73 | 0.4628 | 1.0584 | 1.7302 |
| ALP159 | *S. myrsinifolia* | Ködnitz | 1176.73 | 0.1469 | 1.29 | 1.3345 |
| ALP160 | *S. myrsinifolia* | Ködnitz | 1178.19 | 0.1469 | 0.3772 | 3.0865 |
| ALP161 | *S. myrsinifolia* | Ködnitz | 1178.19 | 0.1469 | -0.9859 | 1.3064 |
| ALP162 | *S. myrsinifolia* | Ködnitz | 1178.19 | 0.3634 | -0.0108 | 2.5510 |
| **Tree individuals** | **Molecular ID** | **Region** | **Elevation** | **Climate PCA1** | **Soil PCA1** | **Herbivory (%)** |
| ALP163 | *S. myrsinifolia* | Ködnitz | 1410.57 | -0.3025 | -1.0538 | 3.6307 |
| ALP165 | *S. myrsinifolia* | Ködnitz | 1385.15 | -0.3025 | -1.1678 | 2.2677 |
| ALP166 | *S. mielichhoferi* | Ködnitz | 1399.31 | -0.3025 | -1.4679 | 1.2732 |
| ALP167 | *S. mielichhoferi* | Ködnitz | 1396.17 | -0.3025 | 0.0266 | 3.0829 |
| ALP168 | *S. mielichhoferi* | Ködnitz | 1388.94 | -0.3025 | -0.8033 | 2.5196 |
| ALP169 | *S. mielichhoferi* | Ködnitz | 1771.95 | -0.7723 | -0.4302 | 3.3241 |
| ALP170 | *S. mielichhoferi* | Ködnitz | 1774.60 | -0.7723 | -0.9536 | 11.2134 |
| ALP171 | *S. mielichhoferi* | Ködnitz | 1774.60 | -0.7723 | -0.9078 | 4.8978 |
| ALP172 | *S. mielichhoferi* | Ködnitz | 1881.64 | -0.7723 | -0.0222 | 5.7483 |
| ALP173 | *S. mielichhoferi* | Ködnitz | 1882.29 | -0.7723 | 1.1591 | 4.8295 |

**Table S9.** Results of LMs and GAMs testing for β-diversity variations among plants of same altitude bands along elevation.

| **Dataset** | **Measured β-diversity** | **Dataset** | **LM** | **LM** | **LM corrected** | **GAM** | **GAM** | **GAM corrected** |
| --- | --- | --- | --- | --- | --- | --- | --- | --- |
|  |  |  | **F-value** | **p-value** | **p-value** | **F-value** | **p-value** | **p-value** |
| Intraspecific | simple | all metabolites | 14.18 | <0.001 | <0.001 | 6.11 | <0.001 | <0.001 |
| Intraspecific | simple | flavonoids | 16.22 | <0.001 | <0.001 | 7.13 | <0.001 | <0.001 |
| Intraspecific | simple | salicinoids | 7.04 | 0.009 | 0.013 | 4.64 | <0.001 | <0.001 |
| Intraspecific | simple | primary metabolites | 21.13 | <0.001 | <0.001 | 9.80 | <0.001 | <0.001 |
| Intraspecific | functional | all metabolites | 1.96 | 0.164 | 0.196 | 1.96 | 0.164 | 0.164 |
| Intraspecific | functional | flavonoids | 13.57 | <0.001 | <0.001 | 7.10 | <0.001 | <0.001 |
| Intraspecific | functional | salicinoids | 3.55 | 0.061 | 0.082 | 2.62 | 0.020 | 0.022 |
| Intraspecific | functional | primary metabolites | 21.27 | <0.001 | <0.001 | 6.75 | <0.001 | <0.001 |
| Intraspecific | structural | all metabolites | 8.39 | 0.004 | 0.007 | 4.24 | <0.001 | <0.001 |
| Intraspecific | structural | flavonoids | 0.00 | 0.962 | 0.962 | 5.56 | <0.001 | <0.001 |
| Intraspecific | structural | salicinoids | 0.66 | 0.419 | 0.457 | 3.92 | <0.001 | <0.001 |
| Intraspecific | structural | primary metabolites | 22.84 | <0.001 | <0.001 | 8.64 | <0.001 | <0.001 |
| Whole dataset | simple | all metabolites | 51.87 | <0.001 | <0.001 | 22.66 | <0.001 | <0.001 |
| Whole dataset | simple | flavonoids | 70.11 | <0.001 | <0.001 | 23.13 | <0.001 | <0.001 |
| Whole dataset | simple | salicinoids | 15.73 | <0.001 | <0.001 | 11.24 | <0.001 | <0.001 |
| Whole dataset | simple | primary metabolites | 47.20 | <0.001 | <0.001 | 14.95 | <0.001 | <0.001 |
| Whole dataset | functional | all metabolites | 20.28 | <0.001 | <0.001 | 7.57 | <0.001 | <0.001 |
| Whole dataset | functional | flavonoids | 31.31 | <0.001 | <0.001 | 17.86 | <0.001 | <0.001 |
| Whole dataset | functional | salicinoids | 1.35 | 0.247 | 0.247 | 3.04 | 0.007 | 0.007 |
| Whole dataset | functional | primary metabolites | 26.32 | <0.001 | <0.001 | 7.99 | <0.001 | <0.001 |
| Whole dataset | structural | all metabolites | 29.14 | <0.001 | <0.001 | 12.66 | <0.001 | <0.001 |
| Whole dataset | structural | flavonoids | 60.39 | <0.001 | <0.001 | 18.43 | <0.001 | <0.001 |
| Whole dataset | structural | salicinoids | 2.54 | 0.113 | 0.123 | 5.72 | <0.001 | <0.001 |
| Whole dataset | structural | primary metabolites | 52.00 | <0.001 | <0.001 | 14.48 | <0.001 | <0.001 |

**Table S10.** Results of ANCOVAs testing for β-diversity variations between the first and fourth quartiles of plants based on their climate PCA1, soil PCA1 and herbivory values.

| **Predictor** | **Measured β-diversity** | **Dataset** | **F-value** | **p-value** | **corrected p-value** |
| --- | --- | --- | --- | --- | --- |
| herbivory | simple | all metabolites | 0.20 | 0.658 | 0.702 |
| herbivory | simple | flavonoids | 3.06 | 0.084 | 0.112 |
| herbivory | simple | salicinoids | 4.31 | 0.041 | 0.060 |
| herbivory | simple | primary metabolites | 0.07 | 0.794 | 0.811 |
| herbivory | functional | all metabolites | 6.95 | 0.010 | 0.017 |
| herbivory | functional | flavonoids | 1.04 | 0.311 | 0.373 |
| herbivory | functional | salicinoids | 1.45 | 0.232 | 0.286 |
| herbivory | functional | primary metabolites | 5.02 | 0.028 | 0.043 |
| herbivory | structural | all metabolites | 0.44 | 0.507 | 0.553 |
| herbivory | structural | flavonoids | 0.04 | 0.845 | 0.845 |
| herbivory | structural | salicinoids | 13.90 | <0.001 | <0.001 |
| herbivory | structural | primary metabolites | 2.31 | 0.133 | 0.173 |
| soil | simple | all metabolites | 4.50 | 0.037 | 0.056 |
| soil | simple | flavonoids | 3.69 | 0.059 | 0.083 |
| soil | simple | salicinoids | 11.58 | 0.001 | 0.002 |
| soil | simple | primary metabolites | 21.36 | <0.001 | <0.001 |
| soil | functional | all metabolites | 3.16 | 0.080 | 0.110 |
| soil | functional | flavonoids | 0.84 | 0.361 | 0.411 |
| soil | functional | salicinoids | 9.92 | 0.002 | 0.004 |
| soil | functional | primary metabolites | 9.68 | 0.003 | 0.005 |
| soil | structural | all metabolites | 0.82 | 0.368 | 0.411 |
| soil | structural | flavonoids | 1.77 | 0.187 | 0.236 |
| soil | structural | salicinoids | 8.00 | 0.006 | 0.010 |
| soil | structural | primary metabolites | 5.21 | 0.025 | 0.040 |
| climate | simple | all metabolites | 456.52 | <0.001 | <0.001 |
| climate | simple | flavonoids | 241.59 | <0.001 | <0.001 |
| climate | simple | salicinoids | 99.81 | <0.001 | <0.001 |
| climate | simple | primary metabolites | 172.46 | <0.001 | <0.001 |
| climate | functional | all metabolites | 86.37 | <0.001 | <0.001 |
| climate | functional | flavonoids | 61.27 | <0.001 | <0.001 |
| climate | functional | salicinoids | 18.03 | <0.001 | <0.001 |
| climate | functional | primary metabolites | 15.63 | <0.001 | <0.001 |
| climate | structural | all metabolites | 320.10 | <0.001 | <0.001 |
| climate | structural | flavonoids | 186.76 | <0.001 | <0.001 |
| climate | structural | salicinoids | 0.97 | 0.328 | 0.384 |
| climate | structural | primary metabolites | 216.74 | <0.001 | <0.001 |
